# Subtropical gyre expansion causes Southern Ocean salinification contrary to freshening predictions

**DOI:** 10.1038/s41467-026-75775-2

**Published:** 2026-07-22

**Authors:** Lisan Yu, John M. Toole

**Affiliations:** Woods Hole Oceanographic Institution, Woods Hole, MA USA

**Keywords:** Physical oceanography, Attribution

## Abstract

The “salty-gets-saltier, fresh-gets-fresher” paradigm predicts that an intensifying hydrological cycle should freshen the climatologically fresh Southern Ocean. Here we show the opposite: sea surface salinity increased at ~0.03 decade^−^^1^ across 40–50°S during 2004–2024, most coherently in the Pacific and Atlantic sectors. We attribute this salinification to the poleward expansion of the southern subtropical gyres, which advects saline subtropical water into latitudes of steepest meridional salinity gradient. The Subtropical and Subantarctic Fronts, tracked via the 35 and 34 isohalines, migrate poleward at unequal rates (–0.46° and –0.18° decade^−1^), narrowing the frontal corridor and sharpening the cross-frontal gradient. A mixed-layer budget shows that horizontal advection dominates the salinity trend, roughly tripling the opposing contribution from surface freshwater flux. Forced by poleward-intensifying westerlies and a positive Southern Annular Mode trend, this circulation-driven salinification demonstrates that ocean dynamics can override freshwater forcing, cautioning against interpreting salinity trends as direct fingerprints of the hydrological cycle.

## Abstract (web summary)

This study shows that the Southern Ocean is becoming saltier rather than fresher, as the poleward expansion of subtropical gyres transports high-salinity waters into higher latitudes, demonstrating that ocean circulation can outweigh surface freshwater forcing in shaping regional salinity patterns, with important implications for ocean ventilation, nutrient cycling, and Earth’s carbon and heat budgets.

## Introduction

The South Pacific, South Atlantic, and South Indian subtropical gyres that dominate midlatitude circulation in the Southern Hemisphere are connected by interbasin pathways into a loosely coupled hemispheric “supergyre”^1–4^. Spanning roughly 20°S to 40–50°S, the individual gyre in each basin is characterized by persistent anticyclonic surface flow driven by the wind stress curl beneath subtropical high-pressure systems. Within their cores, enhanced evaporation, suppressed precipitation, and Ekman convergence produce pronounced sea surface salinity (SSS) maxima^5–7^, making these among the saltiest surface waters on Earth^8^. Along the southern gyre flanks, between the Subtropical Front (STF; approximately 35–45°S) and the Subantarctic Front (SAF; approximately 45–55°S), the influence of the Antarctic Circumpolar Current (ACC) sharpens meridional temperature and salinity gradients and ventilates the thermocline through mode and intermediate water formation, particularly Subantarctic Mode Water^9^. We refer to this transitional corridor as the Subantarctic Zone (SAZ)^10,11^.

Since the early 1990s, the subtropical gyres have intensified and expanded poleward, consistent with a positive trend in the Southern Annular Mode (SAM) and associated changes in wind stress curl^12–17^. Strengthened, poleward-displaced westerlies have modified Ekman transport across the Southern Ocean and displaced the zero wind-stress-curl latitude southward^13,18^. Satellite altimetry reveals large positive sea surface height (SSH) trends in the western subtropical basins, particularly the South Pacific, where steric height anomalies derived from Argo density profiles extend to at least 2000 m depth and account for more than half of the altimetric SSH trend^14,19–21^, indicating that the rise is largely steric-driven rather than mass-driven. Thermocline deepening and poleward boundary displacement are most pronounced in the South Pacific and South Atlantic, while the Indian sector shows greater variability^21–24^. In the Pacific and Atlantic, poleward-intensifying westerlies displace the zero wind-stress-curl line and the ACC’s northern fronts coherently southward, effectively expanding the gyres from their equatorward and poleward flanks^25^. In the Indian Ocean, the Agulhas Return Current (ARC) introduces additional complexity, as its retroflection geometry drives interannual-to-decadal meridional variability in STF position that can partially obscure the underlying secular expansion signal. Variations in ARC retroflection also regulate the magnitude of Agulhas leakage into the South Atlantic^26,27^, linking Indian Ocean boundary current dynamics to salinity variability across the broader subtropical system.

These circulation changes co-occur with widespread upper-ocean warming of approximately 0.1–0.2 °C decade^−1^ centered on 30–50°S^28,29^, amplified in western boundary current extensions, particularly the East Australian Current (EAC) and the Brazil Current^15,21,30^. Surface salinity changes in this region have proven more enigmatic. The prevailing “salty-gets-saltier, fresh-gets-fresher” paradigm, in which wet regions freshen and dry regions salinify under an intensifying hydrological cycle^31,32^, predicts net freshening poleward of ~40°S, where climatological precipitation exceeds evaporation. Early hydrographic syntheses, constrained by sparse pre-Argo sampling, appeared broadly consistent with this expectation^33,34^. But recent high-resolution observations, including those presented here, reveal a more complex picture and raise a fundamental question: can dynamic ocean circulation changes overwhelm the surface freshwater signature of an intensifying water cycle?

Here we present observational evidence that they can. Using two decades of Argo observations combined with satellite altimetry, winds, and evaporation-minus-precipitation data, we show that the climatologically fresh South Pacific and South Atlantic poleward of 35°S has salinified during 2004–2024, with peak trends of ~0.03 decade^−1^ at 40–50°S. We attribute this unexpected salinification to poleward migration of the subtropical gyres, which extends the subtropical high-salinity zone into higher latitudes and produces the strongest salinity trends where the meridional salinity gradient is steepest. A process-based mixed-layer salinity budget quantifies the relative contributions of horizontal advection, entrainment, and net surface freshwater fluxes; we further assess how ENSO and the SAM modulate gyre displacement on interannual to decadal timescales. These results demonstrate that ocean dynamical processes can dominate surface freshwater forcing in shaping regional salinity patterns, with implications for mode water formation, ocean ventilation, upper-ocean nutrient cycling, and the Southern Ocean’s role in regulating Earth’s carbon and heat budgets.

## Results

### Surface salinity trends and associated vertical structure

**Fig. 1:**
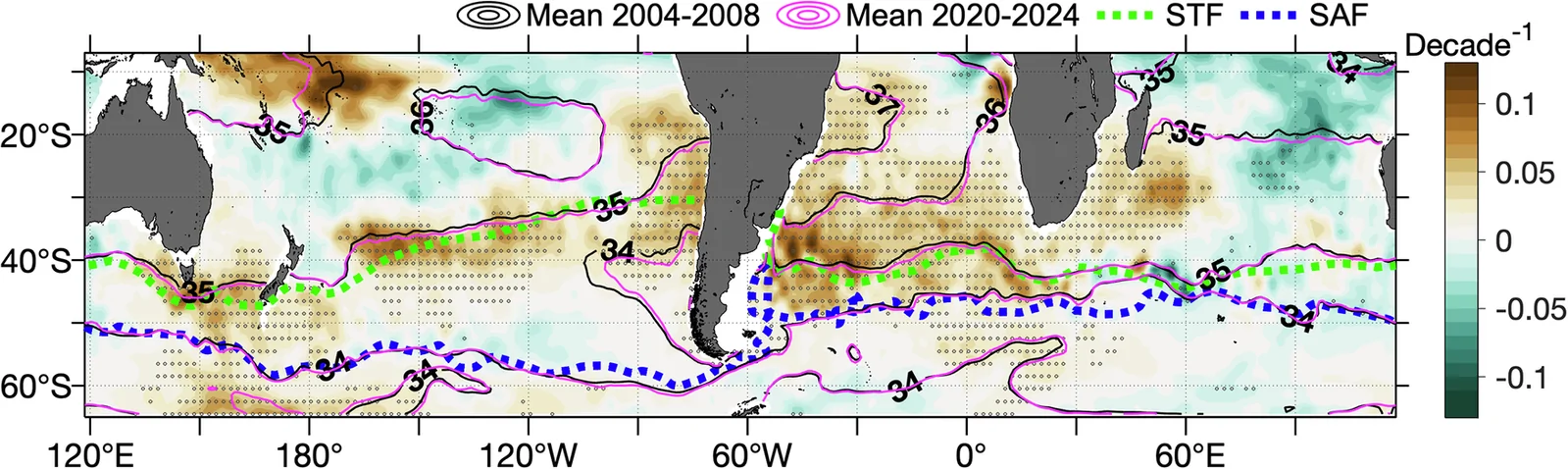
Southern Ocean surface salinity trends and frontal displacement (2004–2024). Shading shows linear trends in sea surface salinity (SSS; decade^−1^), stippled where significant at *p* < 0.05 (see “Methods”). Black and magenta lines show the mean position of the S(34) and S(35) isolines for 2004–2008 and 2020–2024, respectively, which serve as proxies for the Subantarctic Front (SAF) and Subtropical Front (STF). Climatological positions of the STF (green dotted) and SAF (blue dotted) are shown for reference. A coherent band of salinification extends along the southern flanks of the subtropical gyres and through the Subantarctic Zone, with the poleward displacement of S(35) and S(34) most pronounced in the Pacific and Atlantic sectors; patterns are more heterogeneous in the Indian sector.

Over 2004–2024, SSS increased across broad regions of the Southern Ocean (Fig. 1), contradicting the freshening expected under the prevailing “salty-gets-saltier, fresh-gets-fresher” paradigm. The salinification forms a coherent zonal belt of positive trends between the STF and SAF^35^ across the South Pacific, South Atlantic, and western Indian Ocean. Estimated trends over this 21-year period are typically 0.02–0.06 decade^−1^, with local maxima exceeding 0.10 decade^−1^ and diminishing equatorward of the STF and poleward of the SAF. The salinifying belt coincides with a poleward migration of surface isohalines, most clearly S(35) and S(34), whose 2020–2024 mean latitudes lie significantly poleward of their 2004–2008 positions. Because S(35) closely tracks the STF and S(34) closely tracks the SAF, we use them as surface-isohaline proxies for these fronts, defining SAZ width as their meridional separation. With a global time-mean SSS of approximately 34.9, S(35) marks the transition from high-salinity subtropical to low-salinity subantarctic surface waters.

Basin contrasts are pronounced. In the South Pacific (120°E–85°W), salinification of approximately 0.04 decade^−1^ peaks near and just poleward of the STF around 40–50°S. An independent 30-year transect south of Australia (~140°E) corroborates this pattern, documenting sustained summer SSS increases of roughly 0.05 decade^−1^ north of the SAF and a poleward displacement of the STF^36^. In the South Atlantic (85°W–20°E), positive trends span the basin with maxima near the STF. In the South Indian Ocean (20°E –120°E), trend estimates are weaker and patchier, with alternating regions of salinification and freshening and only isolated maxima near the STF. The zonal-mean latitude of S(35) (Supplementary Fig. 1) shows a persistent poleward displacement in the Pacific and Atlantic during 2004–2024 but a comparatively small displacement in the Indian sector. Consistent with this surface contrast, the Indian sector also lacks the coherent subsurface salinification at 40–50°S and the penetration of the subtropical high-salinity layer to ~500 m seen in the Pacific and Atlantic (Supplementary Fig. 2). Two factors contribute to this basin contrast: the wind-stress curl trend in the southern Indian Ocean is weaker and spatially less organized than in the other basins^37,38^ (Supplementary Note 1; Supplementary Fig. 3a, d), and STF position is strongly influenced by Agulhas Return Current retroflection variability, which can mask the secular gyre-expansion signal^25–27^. Given these basin-scale differences, subsequent analyses therefore focus on the Pacific and Atlantic sectors.

**Fig. 2:**
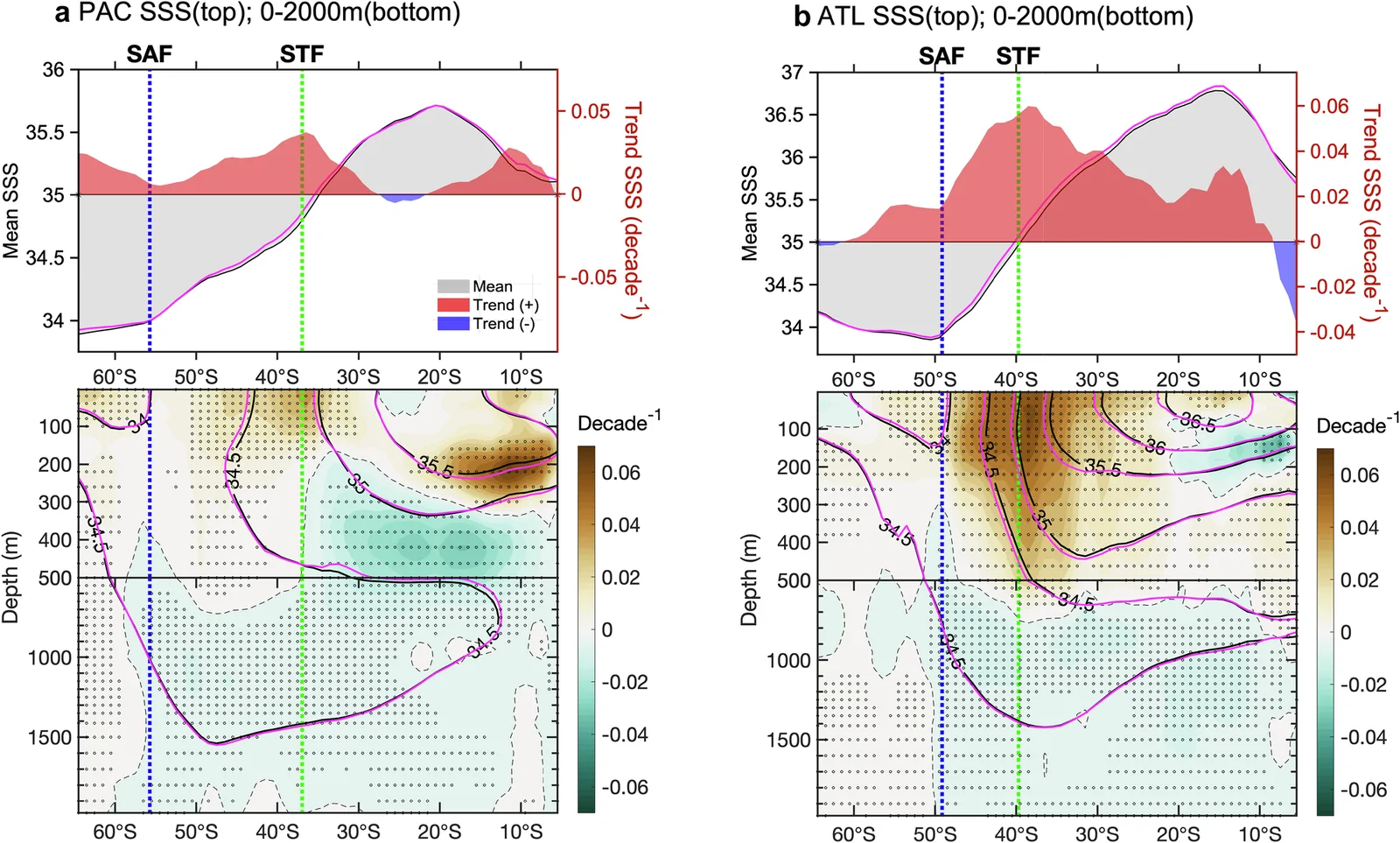
Zonal-mean salinity structure and trends in the South Pacific and Atlantic sectors. **a** Pacific (PAC, 120°E–85°W). **b** Atlantic (ATL, 85°W–20°E). Upper panels: SSS climatology (gray) and linear trend (red, positive; blue, negative) as a function of latitude; black and magenta curves show mean SSS for 2004–2008 and 2020–2024, respectively. Lower panels: linear salinity trend from 0 to 2000 m depth (shading) with an expanded view of the upper 0–500 m; stippled where significant at *p* < 0.05. Black and magenta contours denote mean isohalines for 2004–2008 and 2020–2024, respectively. Vertical dashed lines mark the climatological STF (green) and SAF (blue), which coincide at the surface with the S(35) and S(34) isohalines, respectively, and define the sharp SSS gradient across the STF–SAF corridor. All trends are in decade^−1^. Salinification peaks near 40–50°S and penetrates to ~500 m depth, consistent with a poleward extension of the subtropical high-salinity layer and a poleward shift of surface isohalines.

Zonal-mean SSS profiles (Fig. 2, upper panels) reveal the meridional structure of these trends. In the Pacific, the largest positive SSS trends coincide with the sharp meridional SSS gradient between S(35) and S(34) near 40–50°S (the STF–SAF corridor). In the South Atlantic, comparably large trends extend equatorward of the STF into the subtropical gyre interior, consistent with salinity accumulation associated with a weakening Atlantic Meridional Overturning Circulation (AMOC) and a strengthening South Atlantic subtropical gyre circulation^39–41^. Importantly, these trend peaks occur within waters fresher than the global mean (around 34.9), demonstrating salinification of the climatologically low-salinity band where an intensifying hydrological cycle would predict freshening. Equatorward of S(35) in the Pacific, the saltier subtropics show weaker, patchier increases; in the South Atlantic, salinification remains comparably strong equatorward of S(35), persisting across the subtropical gyre interior. Poleward of S(34), SSS trends diminish south of approximately 55°S in the Atlantic but remain weakly positive south of the SAF in the Pacific. The climatologically fresh 40–50°S band thus experiences substantial salinification, a pattern fundamentally inconsistent with surface freshwater forcing alone. Domain-mean SSS trends confirm that the relative magnitude of salinification between fixed latitude bands varies by basin (Supplementary Fig. 4). In the Pacific, the 40–50°S trend exceeds the 20–35°S trend (0.024 versus 0.009 decade^−^^1^), but only the former is statistically significant (*p* < 0.05). In the Atlantic, both bands show significant positive trends, with the 40–50°S band again the stronger of the two (0.047 versus 0.038 decade^−^^1^; both *p* < 0.05). We therefore describe the salinification mainly in relation to the STF and its poleward displacement, because the contrast between the two bands varies in both strength and significance between basins.

Depth–latitude salinity trend sections (Fig. 2, lower panels) reinforce this picture, revealing a vertically coherent salinification maximum centered near 40–50°S. The signal extends to ~500 m and peaks in the upper thermocline (~100–300 m), with positive trends reaching farther equatorward in the Atlantic than in the Pacific. Trend magnitudes reach 0.02–0.03 decade^−^^1^ in the Pacific and 0.03–0.05 decade^−^^1^ in the Atlantic. The patterns in the two basins diverge equatorward of the S(35) contour. In the Pacific, trends weaken north of 30°S but strengthen again north of 20°S, and a pronounced freshening at 300–500 m forms the dominant subsurface feature north of the STF. In the Atlantic, trends strengthen immediately equatorward of S(35) and weaken only north of ~35°S; freshening is confined to 100–250 m north of 20°S, while modest salinification persists at 300–500 m. South of S(34), patchy subsurface salinification is present in both basins. The overlaid S(35) isohaline is displaced poleward in both basins and downward throughout much of the water column in the Atlantic, consistent with poleward extension of the subtropical high-salinity layer into the STF–SAF corridor.

Because the analysis is performed in an Eulerian frame, fixed-depth salinity trends combine salinity changes on density surfaces with the heave effect of vertically displaced isopycnals. We decompose the fixed-depth salinity trend as, (∂*S*/∂*t*)|_*z*_ ≈ (∂*S*/∂*t*)|_*σ*_ − (∂*S*/∂*z*) × (∂*z*_*σ*_/∂*t*), where (∂*S*/∂*t*)|_*z*_ is the Eulerian salinity trend at fixed depth, (∂*S*/∂*t*)|_*σ*_ is the salinity trend on density surfaces (water-mass change), and −(∂*S*/∂*z*) × (∂*z*_*σ*_/∂*t*) is the heave contribution from the change in depth of density surfaces (Supplementary Figs. 5 and 6). In the upper 500 m of the 40–50°S band, both components are positive in each basin, and the Pacific–Atlantic mean (~0.014 decade^−^^1^) comprises ~60% heave and ~40% salinity change on density surfaces, with heave the larger contributor in the Pacific and water-mass change the larger contributor in the Atlantic (~75%). Both terms are dynamical. The heave component reflects a deepening of subtropical isopycnals at fixed locations, which can arise either from local downwelling driven by the poleward-shifting, downwelling-favorable wind-stress curl, or from the lateral displacement of sloping isopycnals as the subtropical gyre expands poleward. In both cases, a deeper and saltier portion of the water column is drawn to fixed depths, and both pathways are kinematic signatures of the wind-driven circulation change documented above. The density-surface component reflects poleward advection of saltier subtropical water masses along isopycnals, most pronounced in the Atlantic. This component is concentrated in the surface and shallow thermocline because the subtropical salinity maximum is itself an upper-ocean feature: poleward displacement of subtropical waters produces the largest salinity changes on shallow density surfaces, where the climatological contrast between subtropical and subantarctic waters is greatest, and the signal weakens with depth as the contrast diminishes. Neither component is a thermodynamic response to local freshwater flux. The subsurface salinification is therefore a coherent dynamical redistribution of the subtropical high-salinity layer rather than an Eulerian artifact of poleward-migrating isohalines.

### Gyre migration from the Helmholtz transport streamfunction (*ψ*)

To diagnose the circulation driving the poleward displacement of subtropical waters evident in Fig. 2, we compute a Helmholtz transport streamfunction (*ψ*) following the Methods. Absolute geostrophic transport, referenced to ocean-surface absolute dynamic topography (ADT), is combined with Ekman transport from wind stress, and a Helmholtz decomposition isolates the nondivergent, rotational component of the depth-integrated flow^42–44^. The resulting *ψ* represents the rotational, depth-integrated gyre circulation, and its zero contour, *ψ*(0), provides an objective gyre boundary.

Satellite ADT and Argo dynamic height (0–2000 dbar), along with satellite wind-stress curl, have all shifted coherently poleward since 2004, revealing a coordinated large-scale circulation reorganization (Supplementary Figs. 3 and 7). After removing the global mean sea-level rise of 3.7 cm decade^−^^1^, positive ADT trends span the subtropical gyres north of the SAF, with the 2020–2024 mean contours lying poleward of their 2004–2008 counterparts. Dynamic height shows the same behavior, with positive trends across the gyres and poleward displaced 20–25 dyne cm contours in 2020–2024 relative to 2004–2008. Wind forcing shifts similarly, with positive wind stress curl trends (downwelling favorable) near the STF; the zonal-mean zero-curl latitude over the Pacific and Atlantic migrates poleward at −0.46 ± 0.43° decade^−^^1^ (*p* < 0.05; Supplementary Fig. 3e). These coherent signals are consistent with a Sverdrup-type interior adjustment, implying a poleward translation of gyre streamlines.

**Fig. 3:**
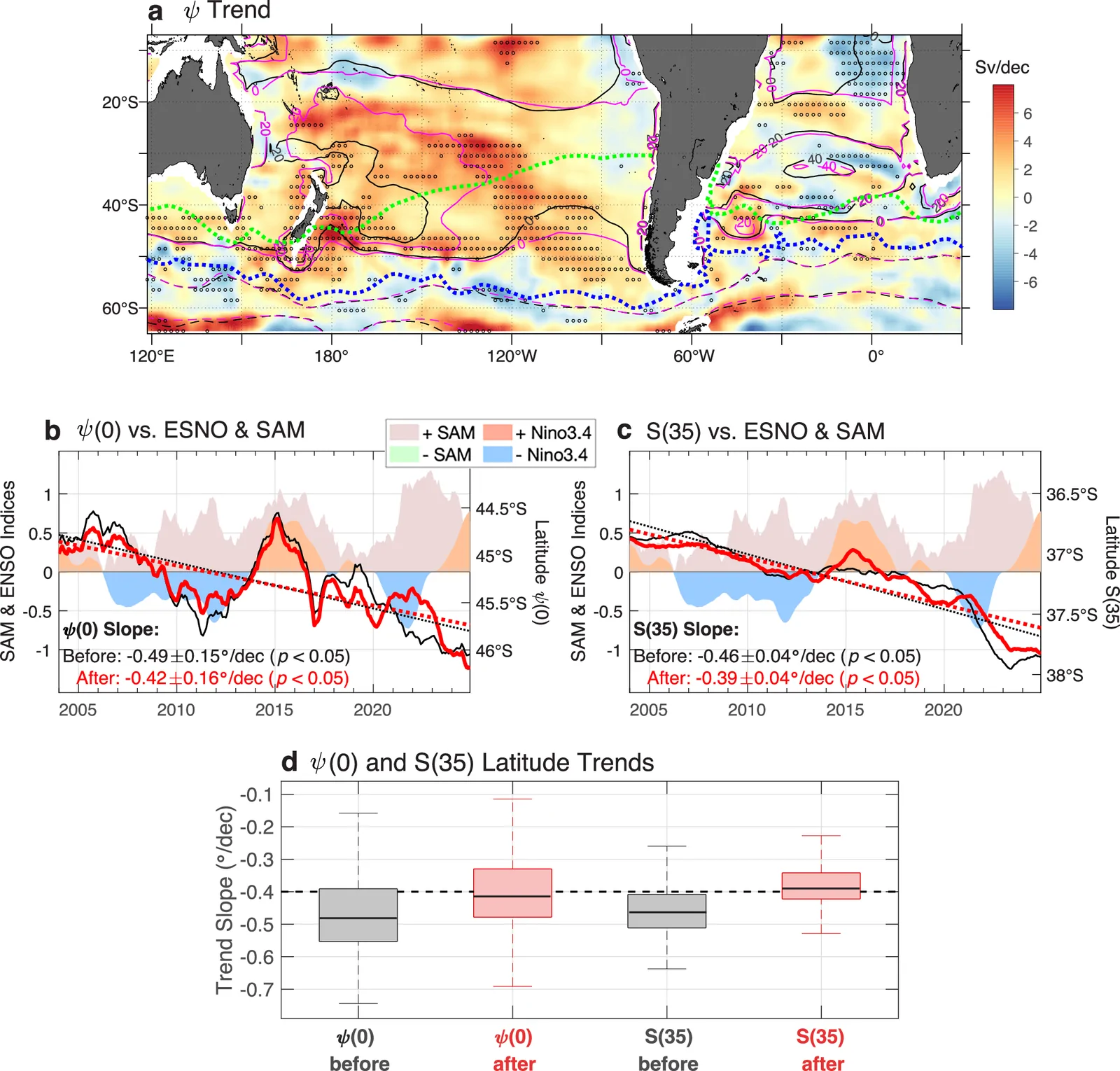
Gyre migration from the Helmholtz transport streamfunction (*ψ*) and influence of ENSO and SAM. **a** Linear trend in *ψ* (Sv decade^−^^1^; shading), stippled where significant at *p* < 0.05. Mean *ψ* contours at −100, 0, 20, and 40 Sv are shown for 2004–2008 (black) and 2020–2024 (magenta); negative values are dashed. Dotted lines mark the climatological STF (green) and SAF (blue). **b** Latitude of the *ψ*(0) gyre-edge separatrix (25-month running mean) before (black) and after (red) removing ENSO + SAM variability; the dashed line is the linear trend fitted to the unsmoothed monthly series. Background shading indicates standardized SAM (light pink, positive; green, negative—not observed, as SAM remained positive throughout the study period) and Niño-3.4 (gold, positive; blue, negative). **c** as in **b**, but for S(35). **d** Trend slopes (° decade^−^^1^) before (black) and after (red) ENSO + SAM removal; boxes show medians and interquartile ranges, and whiskers show the 2.5th–97.5th percentile (95% CI) of the bootstrap distribution (Methods). Poleward migration persists at ~0.4° decade^−1^ for both *ψ*(0) and S(35) after removal of interannual variability.

The *ψ* trend map (Fig. 3a) confirms poleward gyre migration in both basins. In each, opposite-signed large-scale trend anomalies straddle *ψ*(0), but the sign order differs. In the South Pacific, positive anomalies occupy the gyre interior and extend to the southern limb of *ψ*(0), with a narrow negative band just poleward of it. This positive-inside/negative-outside dipole indicates that transport increases are concentrated on the equatorward flank of *ψ*(0) with compensating decreases on the poleward flank, a spatial structure that displaces the zero streamline poleward regardless of whether the gyre intensifies overall^14,15,20^. In the South Atlantic, interior *ψ* trends are weaker and patchier; along the southern limb of *ψ*(0) (50°W–20°E), the dipole reverses (negative inside, positive outside), consistent with poleward displacement accompanied by limited interior intensification. In both basins, mean ψ contours shift poleward between 2004–2008 and 2020–2024 (Supplementary Fig. 8), with a western-intensified enhancement near the Brazil–Malvinas confluence (BMC) consistent with documented frontal variability and poleward excursions^45–47^.

To quantify the boundary translation and its surface expression, we track the latitudinal evolution of *ψ*(0) and S(35) (Fig. 3b, c). Both migrate poleward at comparable rates, with *ψ*(0) trending at −0.49 ± 0.15° decade^−^^1^ and S(35) at −0.46 ± 0.04° decade^−^^1^ (*p* < 0.05). The close agreement between these independent circulation and salinity metrics provides strong evidence that gyre expansion drives the observed salinification. Both rates also align with the poleward shift of the zero wind-stress-curl latitude ( − 0.46 ± 0.43° decade^−^^1^; Supplementary Fig. 3e). Consistent with prior work linking SAM to the latitude and strength of the Southern Hemisphere westerlies and ENSO to SAM variability^48,49^, interannual excursions in *ψ*(0) and S(35) covary with SAM and Niño-3.4 (Fig. 3b, c).

Regression attribution (Supplementary Fig. 9) indicates that *ψ*(0) is paced primarily by combined ENSO + SAM forcing (~22% of variance), whereas S(35) shows lower explained variance (~5%), consistent with salinity integrating wind-driven circulation changes over time rather than responding directly to atmospheric indices. Removing the linear ENSO + SAM signal reduces but does not eliminate the trends (Fig. 3d); *ψ*(0) weakens from −0.49 to −0.42° decade^−^^1^ and S(35) from −0.46 to −0.39° decade^−^^1^ (*p* < 0.05), a 10–15% reduction confirmed across bootstrap realizations (“Methods”). The modest reduction arises because the regression captures only the interannual component of ENSO and SAM variability; the secular positive SAM trend over 2004–2024, which forms part of the long-term poleward intensification of the Southern Hemisphere westerlies^36,48–50^, is retained in the adjusted time series and accounts for the persistent residual drift. SAM therefore acts at two distinct timescales, as a source of interannual modulation of gyre-edge and frontal positions, and as a driver of the multidecadal atmospheric reorganization underlying the secular migration. The ENSO + SAM decomposition separates these contributions; the residual poleward drift of about −0.4° decade^−^^1^ that persists, coincident with the period’s positive SAM tendency and the observed poleward shift of the zero-curl latitude, indicates a multidecadal climate-driven reorganization superimposed on interannual variability.

### Mixed-layer salinity budget and dominant contributions

**Fig. 4:**
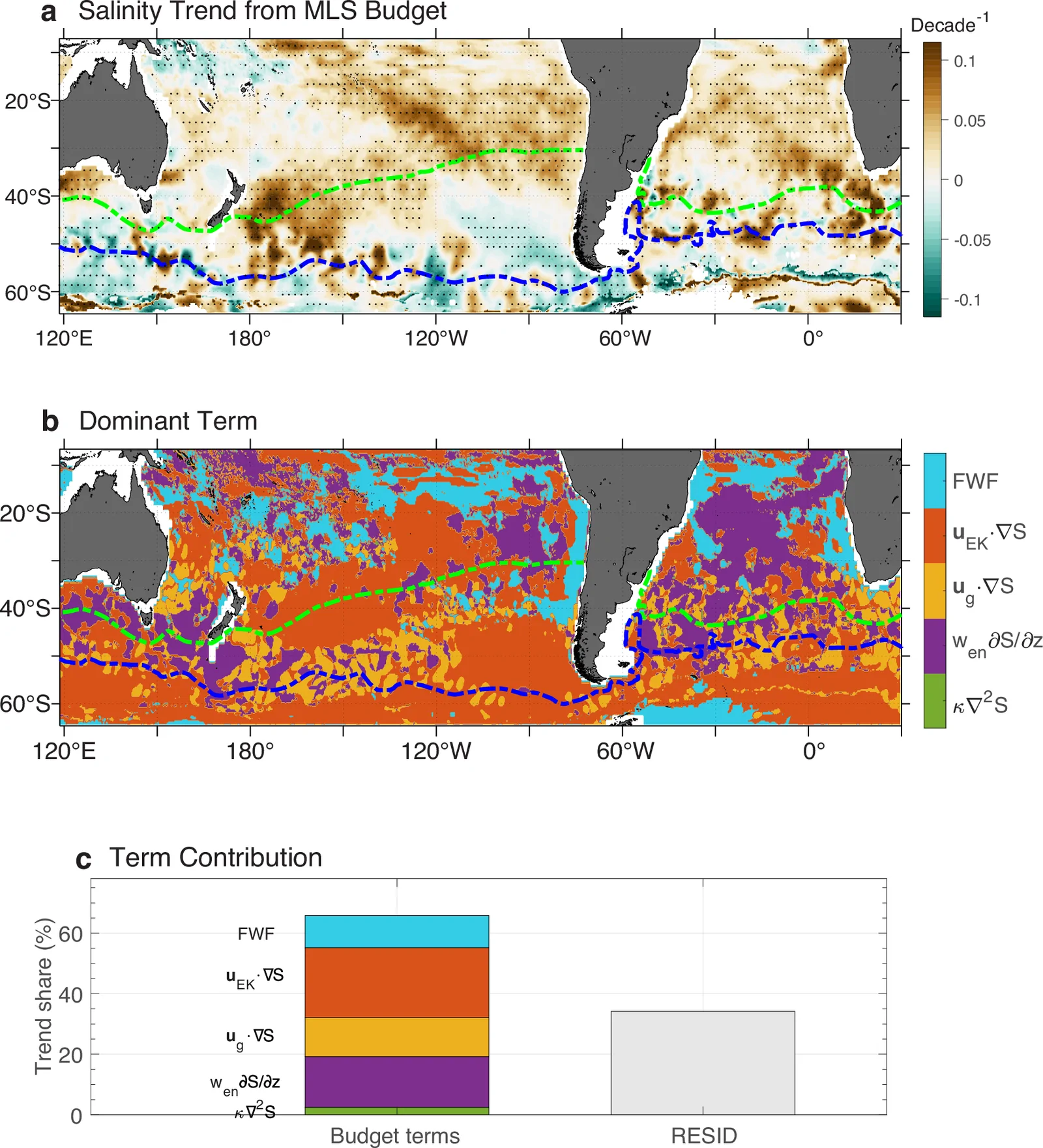
Mixed-layer salinity (MLS) trend attribution, 2004–2024. **a** Budget-reconstructed decadal MLS trend (decade^−1^), computed as the sum of surface freshwater flux (FWF; *S*_0_(*E *−* P)*/*h*), Ekman advection (**u**_EK_⋅∇*S*), geostrophic advection (**u**_g_⋅∇*S*), entrainment (*w*_*en*_∂*S*/∂*z*), and horizontal diffusion (*κ*_ℎ_∇^2^*S*) (“Methods” Eq. 7). Stippled where significant at *p* < 0.05. **b** Locally dominant budget term at each grid point, defined by the largest absolute contribution: FWF (cyan), Ekman advection (red), geostrophic advection (yellow), entrainment (purple), and horizontal diffusion (green). **c** Area-weighted trend share (%) of each term relative to the observed SSS trend. The five diagnosed terms account for 66% of the observed trend (Ekman advection, 23%; entrainment, 17%; geostrophic advection, 13%; FWF, 11%; diffusion, 2%), with a 34% trend-share residual. The climatological STF (green dashed) and SAF (blue dashed) are overlaid in (**a**) and (**b**).

The *ψ*(0) poleward shift co-locates with the 40–50°S salinification band (Figs. 1 and 2), but *ψ* is a depth-integrated, nondivergent diagnostic that cannot attribute salinity trends to specific processes. We therefore diagnose the mixed-layer salinity (MLS) budget^5,7,51^, partitioning its decadal trend among Ekman advection (*C*_EK_), geostrophic advection (*C*_*g*_), entrainment across the mixed layer base (*C*_ENT_), net surface freshwater flux (*C*_FWF_), and horizontal diffusion (Methods; Supplementary Fig. 10). The budget-reconstructed MLS trend *C*_TOT_ (Fig. 4a) reproduces the dominant spatial features of the observed decadal SSS trend (Fig. 1), including the salinification organized around the STF and adjacent frontal transition, additional subtropical salinification at 20–35°S, and patchy negative trends south of the SAF.

In the climatological mean, Ekman flux divergence freshens the SAZ. Northward Ekman transport carries relatively fresh subantarctic surface waters equatorward across the northward-increasing salinity gradient, yielding −*v*_EK_ ∂*S*/∂*y* < 0. A decomposition of the 2004–2024 meridional Ekman tendency trend into velocity, gradient, and covariance contributions (Supplementary Fig. 11) shows that at 40–50°S the sign of the trend reflects the balance among these terms. In the Pacific, weakening northward Ekman transport associated with the poleward shift of the westerly wind maximum is the primary positive contribution, outweighing the opposing gradient term. In the Atlantic, both terms contribute to freshening; salinification there is driven primarily by geostrophic advection and entrainment (Supplementary Fig. 10b, c). The full Ekman contribution *C*_EK_, including both zonal and meridional components, remains positive at 40–50°S (Supplementary Fig. 10a).

Horizontal advection, the sum of Ekman and geostrophic contributions, is the leading driver across the STF–SAF corridor, demonstrating that dynamic ocean circulation overwhelms surface freshwater forcing in this region. *C*_EK_ exhibits a strong, coherent positive band along and just south of the STF across the Pacific (~35–45°S), directly overlapping the 40–50°S salinification zone, a positive but spatially confined signal near the BMC in the Atlantic, and negative contributions south of the SAF in both basins (Supplementary Fig. 10a). The geostrophic contribution *C*_*g*_ is more spatially concentrated, peaking at the BMC and along the SAF in both basins, with weaker and patchier signals elsewhere (Supplementary Fig. 10b). The Ekman contribution *C*_EK_ is largest across the Pacific STF (Supplementary Fig. 10a), where the velocity weakening is strongest, and these advective maxima align with the poleward displacement of *ψ*(0) and the *ψ*-trend dipole (Fig. 3), linking the budget signal to gyre migration.

Entrainment is an important secondary contributor at 40–50°S, while surface freshwater flux plays a minor role (Supplementary Fig. 10c, d). In both basins, *C*_ENT_ provides a positive contribution along the 40–50°S band. In the Pacific, positive contributions are concentrated along the STF and SAF but are patchy elsewhere; in the Atlantic, *C*_ENT_ is predominantly positive over the subtropical interior north of and along the STF (Supplementary Fig. 10c), consistent with deeper mixing entraining saline ventilated-thermocline waters from the north and fresher Antarctic Intermediate Water (AAIW)-influenced waters^52,53^ from the south (Fig. 2, lower panels). *C*_FWF_ is positive over the subtropical interior north of the STF and negative poleward of it in both basins (Supplementary Fig. 10d), thus playing a minor role at 40–50°S. The diffusion is small and spatially noisy (not shown).

The dominant-term map (Fig. 4b; “Methods”) reveals a spatially coherent process hierarchy. Ekman advection controls the STF region in the Pacific; in the Atlantic, both Ekman and geostrophic advection govern the STF and its immediate flanks. Geostrophic advection leads along the SAF in both basins, more prominently in the Pacific. Poleward of the SAF, Ekman advection remains prevalent, with localized patches of geostrophic advection, entrainment, or freshwater flux dominance. Over the interior subtropics (~20–35°S), freshwater flux and entrainment co-dominate. Integrated over the study domain (Fig. 4c; “Methods”), horizontal advection accounts for the largest share (*C*_EK_ 23%, *C*_*g*_ 13%; total 36%), followed by entrainment (*C*_ENT_ 17%), freshwater fluxes (*C*_FWF_ 11%), and horizontal diffusion (2%), with a residual (RESID) trend share of 34%.

The 34% trend-share residual represents the fraction of the observed decadal SSS trend not reproduced by the budget-reconstructed MLS trend. To assess whether this residual could materially affect the dominant-term attribution, we independently reconstruct the MLS budget from the ECCO v4r4 state estimate, which achieves near-complete closure (residual <1% of the physical tendency terms; Supplementary Note 2 and Supplementary Table 1). ECCO reproduces the observed salinification along the Pacific Subtropical Front (Supplementary Fig. 12), and its trend in total advection (ADV_h_ + ADV_v_) identifies the same coherent salinifying signal at the gyre boundary as the observation-based diagnostic (Supplementary Fig. 13), supporting the dynamical consistency of the advective attribution. We further map the spatial distribution of the trend in the observation-based residual itself (Supplementary Fig. 14), which is small and spatially patchy across the Pacific STF salinification band, with organized signals largely confined to eddy-rich frontal regions of the SAF and the western South Atlantic, where mesoscale eddy salt fluxes contribute to the local tendency but lie outside the spatial scope of a large-scale, time-mean advective decomposition. These analyses indicate that the dominant-term attribution at the gyre boundary is robust and that the geography of the 34% trend-share residual reflects this expected scope rather than a methodological bias in the reconstructed budget.

### STF-centered salinification and fixed-band amplification

**Fig. 5:**
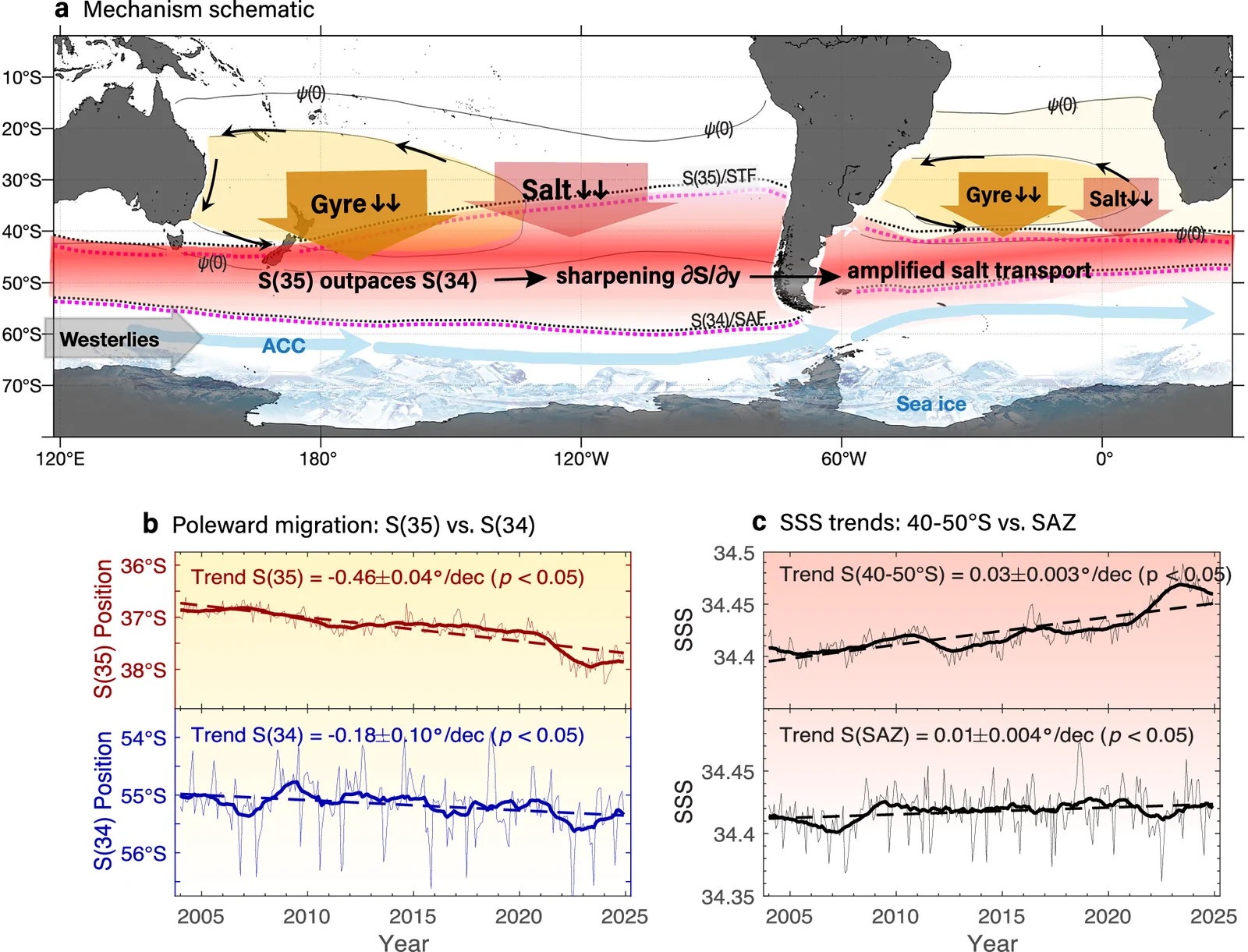
Mechanism and summary of surface salinification at 40–50°S. **a** Mechanism schematic. Broad brown arrows, poleward gyre shifts; broad red arrows, poleward salt advection; red shading, the salinification band. S(35)/STF and S(34)/SAF isohalines (proxies for the Subtropical and Subantarctic Fronts) are shown for 2004–2008 (black dashed) and 2020–2024 (magenta dashed). *ψ*(0) (thin black solid), gyre-edge separatrix from the Helmholtz transport streamfunction; ACC (light blue arrows), Antarctic Circumpolar Current. Central annotation summarizes the amplification mechanism. **b** Poleward migration of S(35) (upper, red) and S(34) (lower, blue): monthly anomalies with seasonal cycle removed (thin), 25-month running mean (thick), and linear trend fitted to unsmoothed monthly values (dashed). Annotated slopes in ° decade^−^^1^ with 95% confidence intervals. **c** SSS averaged over the fixed 40–50°S band (upper) and the front-following Subantarctic Zone (SAZ; lower); formatting as in (**b**); trends in decade^−^^1^. The nearly threefold difference between frames reflects the concentrated salinity change at fixed latitudes as the sharpened front migrates poleward through the 40–50°S band.

The schematic (Fig. 5a) outlines the advective pathway. Poleward shift of the subtropical gyres moves the gyre-edge separatrix *ψ*(0) southward, carrying the high-SSS subtropical zone toward higher latitudes and displacing the STF-centered salinity transition. The fixed 40–50°S band intersects this frontal transition in much of the Pacific and Atlantic and therefore captures a strong salinity response where the meridional gradient is large. However, the relative magnitude of salinification between 40 and 50°S and subtropical latitudes varies by basin and longitude, so the salinification tracks the migrating STF rather than a fixed latitude band, and is strongest where the 40–50°S band intersects the front. The SAZ, defined as the zone between the STF and SAF, is itself a moving control volume; as its boundaries shift poleward, part of the salinity change exits the frame with the migrating fronts rather than accumulating within the zone, so the SAZ-mean trend underestimates the salinification at these latitudes. The fixed 40–50°S frame avoids this ambiguity, capturing the full magnitude of the frontal displacement where it is most pronounced. The SAZ also varies zonally, lying closer to 45–55°S in the Pacific sector (Fig. 5b); the front-following view in Fig. 5c accounts for this asymmetry but yields a smaller, though still statistically significant, trend (~0.01 decade^−^^1^).

Figure 5b documents the differential migration underlying this amplification. S(35) migrates ∼0.46° decade^−^^1^ poleward, whereas S(34) migrates only ∼0.18° decade^−1^, a disparity of ~0.28° decade^−1^ corresponding to a frontal corridor narrowing of ~31 km decade^−1^ at 45°S. This compression sharpens the cross-frontal |∂*S*/∂*y* | , so that as the front migrates poleward through the 40–50°S band, fresher subantarctic surface water is progressively replaced by saltier subtropical water. The fixed 40–50°S band records a salinity trend of ~0.03 decade^−1^ (Fig. 5c), approximately three times the SAZ-mean trend of ~0.01 decade^−1^, with the difference reflecting the concentrated salinity change within the narrow band traversed by the migrating front. Consistent with this mechanism, the dominant-term map (Fig. 4b) shows Ekman advection controlling the mixed-layer salinity budget across most of the STF–SAF corridor in both basins, reflecting the broad-scale influence of wind-driven cross-front transport on the 40–50°S salinification signal. Geostrophic advection emerges as the leading term only in spatially confined patches, most prominently near the BMC in the Atlantic and at scattered locations along the SAF in the Pacific, where strong geostrophic velocity gradients locally amplify the advective contribution.

The contrasting SSS changes between these two frames are summarized in Fig. 5c. In the fixed-latitude (Eulerian) frame, the 40–50°S band records a ∼0.03 decade^−1^ SSS increase as the sharpened STF traverses it, maximizing poleward salt transport. In the front-following (Lagrangian) frame, the SAZ-mean trend is modest (~0.01 decade^−1^) because the control volume moves and contracts with the fronts, carrying part of the imported salt with the shifting boundaries and damping the area-mean change even as local gradients intensify. This frame-dependence has direct implications for monitoring Southern Ocean salinity change. Fixed-latitude observations capture the full magnitude of water mass redistribution, while front-following analyses could underestimate the impact on regional stratification and ventilation pathways. The ~0.02 decade^−1^ difference between frames reflects the partial export of imported salt with the migrating frontal boundaries, consistent with the unequal poleward migration rates of S(35) and S(34) documented in Fig. 5b.

Figure 5a–c thus traces a complete mechanistic pathway from gyre migration to the observed spatial pattern and magnitude of Southern Ocean salinification. Poleward gyre expansion drives a faster poleward shift of S(35) than S(34), narrowing the S(35)–S(34) corridor and sharpening the cross-frontal salinity gradient. As this sharpened front traverses the 40–50°S band, fresher subantarctic water is progressively replaced by saltier subtropical water, producing the observed SSS trend peak. This mechanism may generalize to other frontal systems where differential boundary migration amplifies cross-frontal fluxes, providing a framework for understanding how circulation changes project onto tracer distributions in a changing climate.

## Discussion and implications

The prevailing “salty-gets-saltier, fresh-gets-fresher” paradigm predicts net freshening across the climatologically low-salinity Southern Ocean, yet our results show that this expectation does not hold at 35–50°S. Dynamic ocean circulation changes substantially offset surface freshwater forcing from the intensifying water cycle, driving a wind-driven reorganization of subtropical gyre pathways that increases surface salinity poleward of ~35°S. The salinification is organized most consistently around the STF and adjacent frontal transition, including the 40–50°S band where the meridional salinity gradient between S(35) and S(34) is strong. This finding is consistent with recent evidence that wind-driven geostrophic circulation can drive SSS changes opposite in sign to local freshwater flux anomalies, undermining the regional SSS-FWF relationship on multidecadal timescales^54^. The signal is not surface-limited. Positive salinity trends penetrate to ~500 m at 40–50°S, where both wind-driven deepening of subtropical isopycnals (heave) and salinification of water masses on density surfaces contribute positively in each basin, with heave the larger contributor in the Pacific and water-mass change the larger contributor in the Atlantic. Both are dynamical expressions of the gyre reorganization rather than a thermodynamic response to local freshwater forcing, so the subsurface salinification represents a coherent redistribution of the subtropical high-salinity layer.

The dynamical origin of this salinification is established by three independent diagnostics that converge on comparable poleward translations: the surface S(35) isohaline shifting at −0.46 ± 0.04° decade^-1^, the gyre-edge latitude *ψ*(0) at −0.49 ± 0.15° decade^−1^, and the zero wind-stress-curl latitude at −0.46 ± 0.43° decade^−1^. That these metrics span salinity, dynamic height, and wind forcing yet yield statistically indistinguishable rates strengthens the case for a coherent large-scale dynamical reorganization rather than a locally forced anomaly. ENSO and SAM modulate *ψ*(0) and S(35), shifting them equatorward in El Niño and poleward during La Niña; removing their linear signal reduces but does not eliminate the migration, with residual trends of ∼0.4° decade^−1^ for both. The persistence of these trends, coincident with a more positive SAM tendency and a poleward drift of the zero-curl latitude, points to the evolving Southern Hemisphere westerlies as the multidecadal driver. The Indian sector departs from this picture, exhibiting distinct dynamical behavior arising from weaker and spatially less organized wind-stress curl trends and the strong control of the STF position by Agulhas Return Current retroflection geometry^25–27^, and is therefore treated separately (Supplementary Note 1).

The 40–50°S salinification peak is therefore fundamentally a dynamical phenomenon, its spatial pattern and magnitude set by horizontal advection rather than by any intensification of the local water cycle. Two geometric consequences of the poleward gyre shift combine to produce it. First, poleward gyre migration carries the S(35) and S(34) isohalines across the band of steepest climatological salinity gradient, translating high-salinity subtropical waters into latitudes where the background |∂*S*/∂*y*| is already large. Second, S(35) advances faster than S(34), narrowing the frontal corridor and sharpening the cross-frontal gradient; as this sharpened front migrates poleward through 40–50°S, saltier subtropical water progressively replaces fresher subantarctic water, concentrating the advective salinity increase within the narrow band traversed by the front. The mixed-layer budget confirms this dominance hierarchy: horizontal advection (Ekman + geostrophic) accounts for roughly three times the domain-mean trend share of surface freshwater flux (36% versus 11%), and the water cycle itself freshens the poleward portion of the 40–50°S band, meaning advection must overcome an opposing surface freshwater forcing tendency to produce the observed salinification. This further underscores the central role of the dynamical pathway.

The broader implications of this reorganization span several oceanic processes. Salinification near 40–50°S may locally modulate surface density in a warming Southern Ocean^28^, partly offsetting buoyancy gains from surface warming and freshwater input, though the net effect on stratification is likely to vary seasonally and regionally. Poleward migration of subtropical gyres further shifts the latitudes at which surface waters ventilate the thermocline, rerouting subduction pathways and potentially altering the ventilation of Subantarctic Mode Water and Antarctic Intermediate Water, with downstream consequences for oxygen and nutrient supply to the global thermocline and for carbon sequestration through the solubility pump^55,56^. Poleward displacement of the STF also shifts the boundary between nutrient-rich subantarctic and nutrient-poor subtropical waters, with potential consequences for primary productivity and the ecosystems that track frontal positions. The finding that dynamic redistribution can substantially offset precipitation-evaporation forcing has direct relevance for constraining water cycle amplification from observations, where salinity trends are often used as fingerprints of hydrological cycle change^32–34^.

Continued satellite salinity observations^57,58^ and fine-scale measurements from the Surface Water and Ocean Topography (SWOT) mission will be valuable for monitoring these frontal shifts and their coupling to ocean circulation and the water cycle^59^. The process-based framework developed here is transferable to other ocean basins and testable in climate models. A concise set of metrics, including the latitudes of *ψ*(0) and S(35) and the fixed-band SSS trend at 40–50°S, each directly observable and reproducible in model output, offers a practical basis for model evaluation and for identifying where and when dynamic processes take precedence over surface freshwater forcing when projecting future ocean state. Given that coupled climate models systematically disagree on Southern Ocean salinity responses^34^, these observational benchmarks also provide a mechanistic basis for identifying when and where dynamic ocean processes dominate surface freshwater forcing, a distinction essential for projecting future ocean state and its climate impacts.

## Methods

### Observational data products and uncertainty

#### Data products

Sea surface and subsurface salinity are from the Argo gridded product^60^, providing monthly fields on a 1° × 1° grid from the surface to 2000 dbar. Absolute dynamic topography and surface geostrophic velocities are from the CMEMS Global Ocean Gridded L4 Sea Surface Heights product on a 0.25° daily grid^61^. Dynamic height (0/2000 dbar) is computed from Argo gridded temperature and salinity fields referenced to absolute surface geostrophic velocities. Wind stress and evaporation are from the OAFlux2 all-satellite analysis^62,63^; precipitation is from GPCP v3.2^64^. Mixed-layer depth is computed from Argo profiles using a density threshold of Δ*σ* = 0.03 kg m^−^^3^ relative to 10 m. Salinity is reported on the Practical Salinity Scale 1978 (PSS-78) and is dimensionless; all trend quantities carry units of decade^−1^^65^. All fields are regridded to a common 0.25° monthly grid prior to budget calculation. The study domain covers 120°E–30°E, 65°S–5°S, with a primary analysis period of 2004–2024 and an ECCO validation period of 1992–2017.

#### Data product uncertainty

Argo float coverage in the Southern Ocean was sparse before 2007 and remains lower in the Indian sector than in the Pacific and Atlantic, perhaps responsible for the weaker and patchier salinity signals documented there; the Indian sector is therefore treated separately in Supplementary Note 1. Argo salinity accuracy is approximately 0.01 per profile after delayed-mode quality control, which corrects for conductivity sensor drift – the primary source of systematic bias in Argo salinity records^66^. Altimetric SSH trend uncertainty is approximately 0.3–0.5 cm decade^−1^ after removal of the global mean sea-level trend^61^, small relative to the ADT trends of 2–6 cm decade^−1^ documented in Supplementary Fig. 8. OAFlux wind speed accuracy, evaluated against 126 moored buoys, shows a mean difference of 0.13 m s^−1^ and an RMS difference of 0.71 m s^−1^, with wind direction mean and RMS differences of 0.55° and 17°, respectively^63^. Precipitation uncertainty in GPCP v3.2 tends to be elevated at high southern latitudes, where estimates poleward of 58°S rely mainly on infrared-based retrievals^64^. The impact of these uncertainties on diagnosed trends is assessed through a 12-month block bootstrap applied to all trend estimates, with 95% confidence intervals reported throughout; stippling in all spatial trend maps marks grid cells where trends are significant at *p* < 0.05. The poleward migration of *ψ*(0) and S(35) and the advective dominance in the mixed-layer budget are robust across all bootstrap realizations.

### Trend estimation and uncertainty

Linear trends are estimated by ordinary least squares. Let ${\left\{({t}_{i},\,{Y}_{i})\right\}}_{i=1}^{n}$ denote monthly observations of a scalar field *Y* (e.g., surface salinity) at times *t*_*i*_ measured in years. We fit *Y(t)* = *a + bt* + *ε* and report the slope *b* converted to decade^−1^ units. The standard error (SE) of the slope is

1 $${{\rm{SE}}}\left(\hat{b}\right)=\sqrt{{\hat{\sigma }}^{2}/{\sum }_{i=1}^{n}{\left(t-\bar{t}\right)}^{2}},{\hat{\sigma }}^{2}={\sum }_{i=1}^{n}{\hat{\varepsilon }}_{i}^{2}/(n-2)$$

with residuals ${\hat{\varepsilon }}_{i}^{2}=\,{Y}_{i}\,-\,(\hat{a}+\,\hat{b}{t}_{i})$ and $\bar{t}$ the sample mean of *t*_*i*_. Two-sided 95% confidence intervals are computed as $\hat{b}$ ± *t*_0.975,*n*−2_ × SE($\hat{b}$), where *t*_0.975,*n*−2_ is the Student-*t* critical value with *n* − 2 degrees of freedom; *p* values are obtained from a two-sided *t*-test. The running-mean smoothing shown in the figures is for visualization only and is not used in trend estimation.

### Helmholtz decomposition of the transport field

To objectively track subtropical gyre boundaries and their migration, we decompose the total depth-integrated horizontal transport **U** = (*u, v*) into a rotational component capturing closed gyre circulation (**U**_ψ_) and a divergent component associated with throughflow (**U**_ϕ_) following the Helmholtz theorem^42–44^:

2 $${{{\bf{U}}}}_{{{\uppsi }}}={{\bf{k}}}\times {\nabla }{\psi },{{{\bf{U}}}}_{{{\upphi }}}={\nabla }{{\phi }},{{\bf{U}}}={{\bf{k}}}\times {\nabla }{\psi }+{\nabla }{{\phi }}$$

where **k** is the local upward unit normal, *ψ* is the streamfunction, and *ϕ* is the velocity potential.

The divergent component satisfies ∇× (∇*ϕ*) = 0 and ∇· (∇*ϕ*) ≠ 0, while the rotational component satisfies ∇· (**k** × ∇*ψ*) = 0 and ∇× (**k** × ∇*ψ*) ≠ 0. Taking the horizontal divergence and curl of **U** yields two Poisson equations on the sphere:

3 $${{\nabla }}^{2}{\phi }={\nabla }{{\cdot }}{{\bf{U}}},{{\nabla }}^{2}{\psi }=-{{\bf{k}}}{{\cdot }}({\nabla }\times {{\bf{U}}})$$

solved with impermeable (no-normal-flow) boundary conditions along coasts and island perimeters to ensure a physically consistent separation.

The total transport **U** is obtained by summing the depth-integrated (0-2000dbar) geostrophic transport, derived from gridded Argo temperature and salinity fields^60^ referenced to the ocean surface using absolute surface geostrophic velocities from satellite altimetry^61^, and the Ekman transport computed from satellite scatterometer wind stress. This combination of density-driven geostrophic and wind-driven Ekman transport provides a complete representation of upper-ocean circulation, linking atmospheric forcing changes to water mass redistribution.

The total transport field is mapped to a regular longitude–latitude A-grid, then interpolated to an Arakawa C-grid^67^ to improve coastline representation and minimize numerical artifacts near boundaries. The Poisson equations for *ϕ* and *ψ* are solved using a finite-difference discretization of the spherical Laplacian with impermeable boundaries along coasts and island perimeters. The value of *ψ* is fixed to zero along the American coastlines to provide a consistent cross-basin reference^43^. Disconnected basins are treated independently. The Matlab implementation was based on the algorithm of Stanley^44^.

The resulting *ψ* field represents the rotational, divergence-free gyre circulation, with contours tracing gyre streamlines, extrema marking gyre centers, and magnitudes indicating gyre strength. The *ψ* = 0 contour serves as an objective marker of gyre extent, enabling quantification of meridional shifts in subtropical gyre boundaries over the analysis period. Unlike the Sverdrup streamfunction, which relies solely on wind stress curl under linear vorticity balance, the Helmholtz *ψ* is diagnosed directly from observed total transport, incorporating both wind-driven and buoyancy-driven components and accommodating complex coastlines and basin geometries, and is expressed in Sverdrup (1 Sv = 10^6^ m^3^ s^−1^).

### Frontal isohaline proxies

The latitudinal positions of the S(35) and S(34) isohalines are tracked as fixed-salinity proxies for the surface expressions of the STF and SAF, respectively. These are fixed salinity contours whose positions vary in time; their poleward migration rates serve as diagnostics of frontal displacement rather than as formal dynamical definitions of the fronts. The markedly different migration rates of S(35) and S(34), together with the close agreement between the S(35) migration and the independently diagnosed poleward shift of the gyre-edge streamfunction *ψ*(0), support the interpretation of these isohalines as proxies for the surface expressions of the migrating frontal zones over the study period. The latitude-dependent and basin-dependent SSS trends (Supplementary Fig. 4) further support the interpretation that the observed isohaline shifts reflect frontal-zone displacement rather than a spatially uniform salinity offset.

### Removing ENSO and SAM signals

To isolate the multidecadal trend from interannual climate variability, we regress monthly fields against standardized Niño-3.4 and SAM indices and subtract the fitted component while preserving the long-term trend, allowing us to assess whether gyre migration persists beyond known modes of variability. Let *y*_*s*_(*t*) denote the value at grid cell *s* and month *t* = 1, …, *N*. Let *X*_ENSO_(*t*) and *X*_SAM_(*t*) be the corresponding indices, standardized over the analysis period as $\widetilde{X}$_*k*_(*t*) = [*x*_*k*_(*t*) − *μ*_*k*_]/*σ*_*k*_, where *μ*_*k*_ is the mean and *σ*_*k*_ the standard deviation, for *k* ∈ {ENSO, SAM}. The data at each grid cell are modeled as:

4 $${y}_{s}(t)={\alpha }_{s}+{\beta }_{s,{{\rm{ENSO}}}}{\widetilde{X}}_{{{\rm{ENSO}}}}(t)+{\beta }_{s,{{\rm{SAM}}}}{\widetilde{X}}_{{{\rm{SAM}}}}(t)+{\varepsilon }_{s}(t)$$

In matrix form, with *y*_*s*_ ∈ R^N^, design matrix X = [1, $\widetilde{X}$_ENSO_, $\widetilde{X}$_SAM_] ∈ R^(N×3)^, and parameters *θ*_*s*_ = [*α*_*s*_, *β*_*s*,ENSO_, *β*_*s*,SAM_]^T^, parameters are estimated by ordinary least squares (OLS):

5 $${\hat{\theta }}_{s}={({X}^{T}X)}^{-1}{X}^{T}{y}_{s}$$

The mode-related signal is ${\hat{{{\boldsymbol{y}}}}}_{{{\boldsymbol{s}}}}={{\boldsymbol{X}}}{\hat{{{\boldsymbol{\theta }}}}}_{{{\boldsymbol{s}}}}$. The ENSO and SAM contribution is removed while retaining the mean via

6 $${\hat{{{\boldsymbol{y}}}}}_{{{\boldsymbol{s}}},{\mathrm{adj}}}={{{\boldsymbol{y}}}}_{{{\boldsymbol{s}}}}-{\hat{{{\boldsymbol{y}}}}}_{{{\boldsymbol{s}}}}+{{\bf{1}}}{\hat{\alpha }}_{s}$$

so that *y*_*s*,adj_ (the adjusted field) represents variability with the linear ENSO and SAM contributions removed. This approach assumes a linear, additive relationship with the indices and limited collinearity. Uncertainty in the regression coefficients is characterized using a moving-block bootstrap that resamples consecutive months to preserve temporal dependence (block length ≈1.5 × *N*^1/3^ months); 95% confidence intervals are reported using the percentile method.

### Mixed-layer salinity budget

To identify which physical processes drive the observed salinity changes, we diagnose the mixed-layer salinity budget that partitions the rate of salinity change into contributions from horizontal advection, vertical entrainment, surface freshwater exchange, diffusion, and an unresolved residual. Let *S*(*λ, ϕ, t*) denote mixed-layer salinity and *h*(*λ, ϕ, t*) the mixed-layer depth (density-threshold definition). The budget equation is

7 $$\frac{\partial S}{\partial t}=-({{{\bf{u}}}}_{{{\bf{EK}}}}+{{{\bf{u}}}}_{{{\bf{g}}}})\cdot {\nabla }{{\rm{S}}}-\frac{{w}_{{en}}}{h}({S}_{b}-S)+\frac{{S}_{0}(E-P)}{h}+{\kappa }_{h}{{\nabla }}^{2}S+{{\rm{\varepsilon }}}$$

where **u**_EK_ is the Ekman velocity, **u**_g_ the surface geostrophic velocity from altimetric dynamic topography, *w*_*en*_ the entrainment velocity at the base of the mixed layer, *S*_*b*_ the salinity of a 20 m slab immediately below the mixed layer, *E* − *P* the surface freshwater flux (evaporation minus precipitation, positive for net evaporation), *S*_0_ = 35 a reference salinity, * κ*_*h*_ an effective horizontal mixing coefficient, and *ε* a residual representing processes unresolved in monthly-mean gridded products. Positive terms denote salinification.

#### Term construction

Horizontal gradients are computed by second-order centered differences on the spherical grid. The geostrophic velocity u_g_ is obtained from SSH gradients via the geostrophic relation. The Ekman velocity is **u**_EK_ = **τ** × **k**/(*ρ*_*0*_*fh*), where **τ** is the surface wind stress, *ρ*_0_, a reference seawater density, and *f* the Coriolis parameter. Tkman advection (**u**_EK_ ⋅∇S) and geostrophic advection (**u**_g_⋅∇S) are retained separately to distinguish wind-driven from geostrophic salt transport. Entrainment is parameterized in bulk form as (*w*_*en*_*/h)(S*_*b*_*−S*), with *w*_*en*_ *=* max{0*, Dh/Dt*}, where *Dh/Dt* = *∂h/∂t* + **u** ⋅∇*h*; de-entrainment during mixed-layer shoaling is set to zero. Surface freshwater forcing is computed as *S*_0_(*E* − *P*)/*h*, with *E* − *P* fields co-located monthly to the salinity grid. Horizontal diffusion *κ*_*h*_ ∇^2^S is evaluated with *κ*_*h*_ = 1000 m^2^ s^−1^ and is subdominant in area-weighted diagnostics. All terms are evaluated on the common 0.25° monthly grid. The residual *ε* encompasses the covariance between mixed-layer depth fluctuations and salinity fluxes at the mixed-layer base, sub-monthly rectification of entrainment variability, submesoscale fluxes unresolved on the 0.25° grid, and uncertainties in the observational input fields.

#### Trend contribution decomposition

Each monthly MLS budget term *T*_*i*_(*t*, *λ*, *φ*) in Eq. (7) has units of mon^–1^. To convert these monthly tendencies into contributions to the decadal MLS trend, each term was integrated cumulatively in time:

8 $$\triangle {{{\rm{S}}}}_{{{\rm{i}}}}({t}_{n},\lambda,\varphi )=\mathop{\sum }_{k=1}^{n}{{{\rm{T}}}}_{{{\rm{i}}}}\left({t}_{k},\lambda,\varphi \right)\triangle {t}_{k}$$

where Δ*t*_*k*_ = 1 month and *n* = 1, …, *N* (with *N* = 252 for the 2004–2024 record), so that ΔSᵢ has units of PSS-78 and represents the cumulative salinity change contributed by process *i* from the start of the record to time *t*_*n*_. The decadal trend contribution is then the least-squares linear trend of Δ*S*ᵢ(*t*; *λ*, *φ*), scaled to decade^−1^ units:

9 $${C}_{i}(\lambda,\varphi )=120\,{\mathrm{months}}\times {\mathrm{slope}}\,({\rm\Delta }{S}_{{i}}(t,\lambda,\varphi )),$$

where the fitted slope has units of month^−1^, and multiplication by 120 months converts it to decade^−1^. Thus, *Cᵢ* represents the contribution of process *i* to the linear MLS trend. The budget-reconstructed MLS trend is

10 $${C}_{{TOT}}(\lambda,\varphi )={\sum }_{i=1}{C}_{i}(\lambda,\varphi )$$

shown in Fig. 4a; individual *Cᵢ*(*λ*,*φ*) maps are shown in Supplementary Fig. 10a–d.

#### Dominant-process map

At each grid cell, the budget term with the largest absolute decadal trend contribution |*Cᵢ*(*λ, φ*)| is identified as the locally dominant process (Fig. 4b); ties are resolved by the larger *t*-statistic of the corresponding slope estimate.

#### Trend share partition

The fractional contribution of each budget term to the observed decadal MLS trend is

11 $${\left({{\rm{Trend\; Share}}}\right)}_{i}(\lambda,\varphi )=|{C}_{i}(\lambda,\varphi )|/\max (\varepsilon,\varSigma {\sum }_{i}{C}_{i}(\lambda,\varphi )|+|{C}_{{{\rm{RES}}}}(\lambda,\varphi )|)\times 100\%$$

where *C*_RES_ = *C*_OBS_ − *C*_TOT_, the denominator includes all five diagnosed *C*_*i*_ terms and the residual so that shares sum to unity by construction, and *ε* = 10^–12^ decade^–1^ prevents division by zero. Cells where *C*_OBS_ or any *C*_*i*_ is non-finite are excluded; cosine-latitude weights are renormalized over the remaining valid cells. The domain-mean share for each term is the area-weighted average of (Trend Share)ᵢ(*λ,φ*) over all valid cells, expressed as a percentage (Fig. 4c). The residual trend share represents the fraction of the observed decadal MLS trend not accounted for by the five diagnosed budget terms and is examined further using the ECCO v4r4 state estimate (see below).

#### ECCO-based budget validation

To independently assess the dominant-term attribution shown in Fig. 4c, we reconstruct the MLS budget from the ECCO v4r4 state estimate on its native LLC90 grid (Supplementary Note 2; Supplementary Table 1; Supplementary Figs. 12–14). The ECCO budget is near-closed at the basin scale, with a residual below 1% of the physical tendency terms in both 1992–2017 and 2004–2017 (Supplementary Table 1). ECCO reproduces the observed Argo salinification along the Pacific Subtropical Front (Supplementary Fig. 12), and the trend in ECCO total advection (ADV_h_ + ADV_v_) identifies the same coherent salinifying advective signal at the gyre boundary as the observation-based diagnostic (Supplementary Fig. 13), supporting the dynamical consistency of the advective attribution. We further map the trend in the observation-based budget residual (Supplementary Fig. 14), which is generally small and patchy across the Pacific STF salinification band and is not collocated with the diagnosed advective trend, indicating that the dominant-term attribution at the gyre boundary is robust to the observational residual.

## Supplementary information

Supplementary Information

Transparent Peer Review file

## Data Availability

Argo temperature and salinity profiles are available from the Global Argo Data Repository (https://argo.ucsd.edu/). Satellite altimetry data are from the Copernicus Marine Environment Monitoring Service (https://marine.copernicus.eu/; product identifier SEALEVEL_GLO_PHY_L4_MY_008_047, 10.48670/moi-00148). Ocean evaporation and wind stress data are from the Objectively Analyzed Air-Sea Fluxes (OAFlux) project (https://scienceweb.whoi.edu/oaflux/). Precipitation data are from the GPCP Version 3.3 Satellite-Gauge (SG) Combined Precipitation Data Set (GPCPMON) (https://disc.gsfc.nasa.gov/datasets/GPCPMON_3.3/summary). The monthly SAM index is obtained from the British Antarctic Survey (http://www.nerc-bas.ac.uk/public/icd/gjma/newsam.1957.2007.txt). The monthly Niño-3.4 index is from the NOAA Climate Prediction Center (https://www.cpc.ncep.noaa.gov/data/indices/ersst5.nino.mth.91-20.ascii). The Gibbs SeaWater (GSW) Oceanographic Toolbox v3 (https://www.teos-10.org/software.htm) was used for computing seawater density and thermodynamic properties. Mapping and visualization were performed using M_Map (https://www.eoas.ubc.ca/~rich/map.html). Helmholtz decomposition was implemented following Stanley (https://www.mathworks.com/matlabcentral/fileexchange/75614-2d-helmholtz-decomposition-on-a-c-grid). Processed data used to generate the figures in this study are deposited at the Zenodo record^68^.

## Code availability

The analyses in this study use standard MATLAB routines, as described in the Methods. Code used to generate the figures is available at the same Zenodo record^68^.
