# Supplementary material for: Subtropical gyre expansion causes Southern Ocean salinification contrary to freshening predictions
Source: Nat Commun. 2026 Jul 22;17:8924. doi: 10.1038/s41467-026-75775-2 (PMC13503869; doi:10.1038/s41467-026-75775-2)
Supplement: Supplementary file 1 [file 41467_2026_75775_MOESM1_ESM.pdf]

Supplementary Information

Subtropical gyre expansion causes Southern Ocean salinification contrary to freshening predictions

Lisan Yu[1*] and John M. Toole[1]

Woods Hole Oceanographic Institution

[1] Woods Hole Oceanographic Institution, Woods Hole, MA, USA.

[*] Corresponding author: Dr. Lisan Yu, Physical Oceanography Department, Woods Hole Oceanographic Institution, Woods Hole, MA 02543, USA.

Email: lyu@whoi.edu

Supplementary Figures 1-14

Supplementary Notes 1-2

Table of Contents

Supplementary Notes

Note 1. Indian Ocean surface salinity changes and basin-scale Subtropical Front variability

Note 2. ECCO v4r4 mixed-layer salinity budget and comparison with the observation-based diagnostic

Supplementary Figures

Figure 1. Poleward migration of the surface S(35) isohaline by basin

Figure 2. Zonal-mean salinity climatology and linear trends in the Indian Ocean sector (20°E–120°E)

Figure 3. Trends in wind-stress curl and the zero-curl line position across Southern Hemisphere ocean basins

Figure 4. Domain-mean SSS trends for the 40–50°S and 20–35°S latitude bands

Figure 5. Isopycnal/heave decomposition of the depth–latitude salinity trend (2004–2024).

Figure 6. Depth-integrated decomposition of the 40–50°S fixed-depth salinity trend

Figure 7. Trends and multidecadal changes in absolute dynamic topography (ADT) and dynamic height (DH) across the Southern Hemisphere

Figure 8. Mean Helmholtz transport streamfunction in the Southern Hemisphere subtropical–subpolar gyres and its multidecadal evolution

Figure 9. Climate-mode contributions to the poleward migration of $\psi(0)$ and S(35)

Figure 10. Trend contributions to the mixed-layer salinity budget, 2004–2024

Figure 11. Decomposition of the meridional Ekman salinity tendency trend at 40–50°S (2004–2024)

Figure 12. Mixed-layer salinity trends from Argo (2004–2024) and ECCO v4r4 (1992–2017, 2004–2017)

Figure 13. Trends in horizontal advection from the observation-based budget and total advection from ECCO v4r4 budget

Figure 14. Spatial distribution of the linear trend in the observation-based mixed-layer salinity budget residual

Supplementary References

Supplementary Notes

Supplementary Note 1. Indian Ocean surface salinity changes and basin-scale Subtropical Front variability

The Indian Ocean sector (20°E–120°E) differs from the Pacific and Atlantic in both the surface and subsurface response (Main text Fig.1; Supplementary Fig.1). The zonal-mean latitude of S(35) shows no statistically significant poleward migration over 2004–2024 (+0.02 ± 0.05° decade^{-1}, $p > 0.05$; Supplementary Fig. 1c), compared with significant southward trends of −0.35 ± 0.04° decade^{-1} in the Pacific and −0.41 ± 0.07° decade^{-1} in the Atlantic ($p < 0.05$; Supplementary Fig. 1a,b). The surface SSS trend field shows weak signals of mixed sign across the STF–SAF corridor (main text Fig. 1), and the zonal-mean subsurface section reveals near-zero or weakly negative trends throughout the upper water column at 40–50°S (Supplementary Fig. 2), in contrast to the coherent salinification penetrating to ~500 m observed in the other two basins (main text Fig. 2).

This basin contrast is consistent with differences in the surface forcing. The wind-stress-curl trend in the southern Indian Ocean is weaker and less spatially coherent than in the Pacific and Atlantic (Supplementary Fig. 3a), and the latitude of the zero wind-stress-curl line shows no significant trend (−0.06 ± 0.11° decade^{-1}, $p > 0.05$; Supplementary Fig. 3d). In the Pacific and Atlantic, by contrast, the zero-curl line migrates significantly poleward at −0.54 ± 0.10° and −0.23 ± 0.10° decade^{-1}, respectively ($p < 0.05$; Supplementary Fig. 3b,c), with a combined Pacific–Atlantic trend of −0.46 ± 0.43° decade^{-1} (Supplementary Fig. 3e). The weaker Indian Ocean curl trend is consistent with previous studies showing that wind-stress-curl variability in this sector is strongly modulated by remote ENSO forcing through the Indonesian Throughflow and atmospheric teleconnections via the Walker circulation [1, 2], which can offset or mask the secular westerly trend on the timescales considered here. Frontal position in this sector is also influenced by Agulhas Return Current retroflection variability, which contributes additional interannual-to-decadal variability in the STF location [3, 4].

Given the absence of a significant secular trend in either S(35) or the zero-curl line in the Indian sector, the main-text analysis and mixed-layer salinity budget is presented for the Pacific and Atlantic, where the advective salinification signal is statistically significant, spatially coherent, and amenable to mechanistic decomposition. We do not interpret the Indian Ocean result as evidence that gyre-edge dynamics are absent in this sector, only that any secular signal over 2004–2024 is below the detection threshold against the larger-amplitude interannual variability characteristic of this basin.

Supplementary Note 2. ECCO V4r4 mixed-layer salinity budget and comparison with the observation-based diagnostic

To complement the observation-based mixed-layer salinity (MLS) budget and to assess the dynamical consistency of the diagnosed advective attribution, we performed an independent

budget analysis using the ECCO Version 4 Release 4 (v4r4) ocean state estimate, which achieves near-complete budget closure by construction.

Methodology. We reconstructed the full MLS budget on the ECCO native LLC90 grid following the ECCO budget documentation [5, 6, 7] and previous ECCO-based MLS budget studies [8, 9, 10, 11, 12], including the nonlinear free-surface volume adjustment. The monthly MLS budget is decomposed as

$$\frac{\partial S_{ML}}{\partial t} = \mathrm{FWF} + \mathrm{ADV}_h + \mathrm{ADV}_v + \mathrm{DIFF}_h + \mathrm{DIFF}_v + \varepsilon(\mathrm{t}) \qquad \text{(S1)}$$

where S_{ML} mixed-layer salinity, FWF is the surface freshwater-forcing tendency, ADV_h and ADV_v are the horizontal and vertical advective tendencies, DIFF_h and DIFF_v are the horizontal and vertical diffusive tendencies, and ε(t) is the residual. ε(t) is the time-varying residual, reflecting month-to-month changes in sub-monthly covariance terms that are not captured by monthly mean diagnostics. All tendency terms were regridded to a common 0.5° product, and the cumulative anomaly contribution of each budget term was integrated over two periods within the 26 years of monthly output: 1992–2017 (full reanalysis record) and 2004–2017 (overlapping with the Argo period analyzed in the main text).

Budget closure (Supplementary Table 1). Averaged over the South Pacific and South Atlantic (120°E–30°E, 65°S–5°S), the residual of the annual-mean ECCO budget is ~0.003 PSS-78 yr^{-1}, less than 1% of the total magnitude of the physical tendency terms in both periods (1992–2017 and 2004–2017). The basin-mean salinity tendency is the small net of three large opposing contributions: a salinifying freshwater forcing, a freshening net advection, and a salinifying net diffusion. Under the ECCO nonlinear free-surface formulation, the horizontal and vertical advective components individually carry a volume-flux contribution that nearly cancels between them; only their sum ($\mathrm{ADV_h} + \mathrm{ADV_v}$) is physically interpretable, and we therefore report and analyze this sum throughout.

**Budget Term**	**2004–2017 (PSS-78 yr^{-1})**	**1992–2017 (PSS-78 yr^{-1})**
∂S/∂t	-0.0118	-0.0133
FWF	0.0394	0.0369
($\mathrm{ADV_h}$, $\mathrm{ADV_v}$)	(-6.4293, 6.1874)	(-6.4110,6.1677)
ADV Total	-0.2419	-0.2433
($\mathrm{DIFF_h}$, $\mathrm{DIFF_v}$)	(-0.0023, 0.1896)	(-0.0020, 0.1919)
DIFF Total	0.1873	0.1899
ε (Residual)	0.0033	0.0032

Supplementary Table 1. Annual-mean mixed-layer salinity budget terms averaged over the South Pacific and South Atlantic basins (120°E–30°E, 65°S–5°S), in PSS-78 yr^{-1}, computed from ECCO over two periods. : 2004–2017 (overlapping with part of the Argo record) and 1992–

2017 (full reanalysis period). The large cancellation between ADV_h and ADV_v arises from the volume flux associated with the free-surface formulation in ECCO; only their sum is physically meaningful.

Salinity trend comparison (Supplementary Fig. 12). ECCO reproduces the broad structure of the observed Argo salinity trend, with the closest agreement along the Subtropical Front (STF) in the South Pacific. The Pacific salinification signal is present in both ECCO periods and is more spatially coherent over the longer 1992–2017 record, which reduces the imprint of interannual variability and yields a cleaner trend pattern aligned with the climatological STF position. Over the shorter 2004–2017 window that overlaps with Argo, the modeled signal is patchier but still locates the salinification along the same frontal band. In the South Atlantic, by contrast, the basin-scale salinification seen in Argo is not reproduced in either ECCO period, with the modeled trends remaining weak and spatially fragmented across the 30°–50°S band. This Pacific–Atlantic asymmetry is consistent with the expected skill of ECCO v4r4 in basins dominated by mesoscale eddy activity. The LLC90 grid (~1° at the equator, ~40 km at high latitudes) parameterizes rather than resolves mesoscale eddy fluxes (Forget et al., 2015), a known limitation in regions of strong mesoscale activity and energetic boundary currents[13] such as the Brazil–Malvinas Confluence and Agulhas Return Current, where eddy-driven salt transport contributes substantially to the local salinity tendency[14].

Advective trend comparison (Supplementary Fig. 13). The trend in horizontal advection from the observation-based budget ($-u\cdot\nabla S$, dominated by the Ekman and geostrophic components $u_{EK} + u_g$; Methods Eq.7) and the trend in ECCO total advection ($ADV_h + ADV_v$) identify the same coherent salinifying advective trend along the Pacific STF. In both diagnostics, the salinifying advective signal is collocated with the climatological STF position and aligns spatially with the observed salinity trend in Fig. 8a, providing direct evidence that horizontal advection at the gyre boundary is the leading driver of the Pacific salinification. This agreement is informative because the two diagnostics are methodologically distinct: the observation-based estimate is a direct kinematic decomposition combining satellite altimetry, winds and surface freshwater fluxes, and Argo salinity, while the ECCO estimate results from a dynamically consistent state estimate that incorporates these observations through 4D-Var assimilation. In the Atlantic, the ECCO advective trend is weaker and lacks a comparable frontal signature, paralleling the salinity-trend asymmetry noted above.

Residual diagnostic (Supplementary Fig. 14). The trend in the observation-based budget residual ($C\varepsilon$) is small and spatially patchy across most of the domain and is particularly modest across the Pacific STF salinification band where the advective attribution is centered. Organized residual trends are largely confined to the eddy-rich frontal regions of the Subantarctic Front (SAF) and the western South Atlantic, consistent with the spatial scope of a large-scale, time-mean advective decomposition. The Pacific STF band is not collocated with these organized features, supporting the robustness of the diagnosed advective contribution at the gyre boundary.

Supplementary Figures

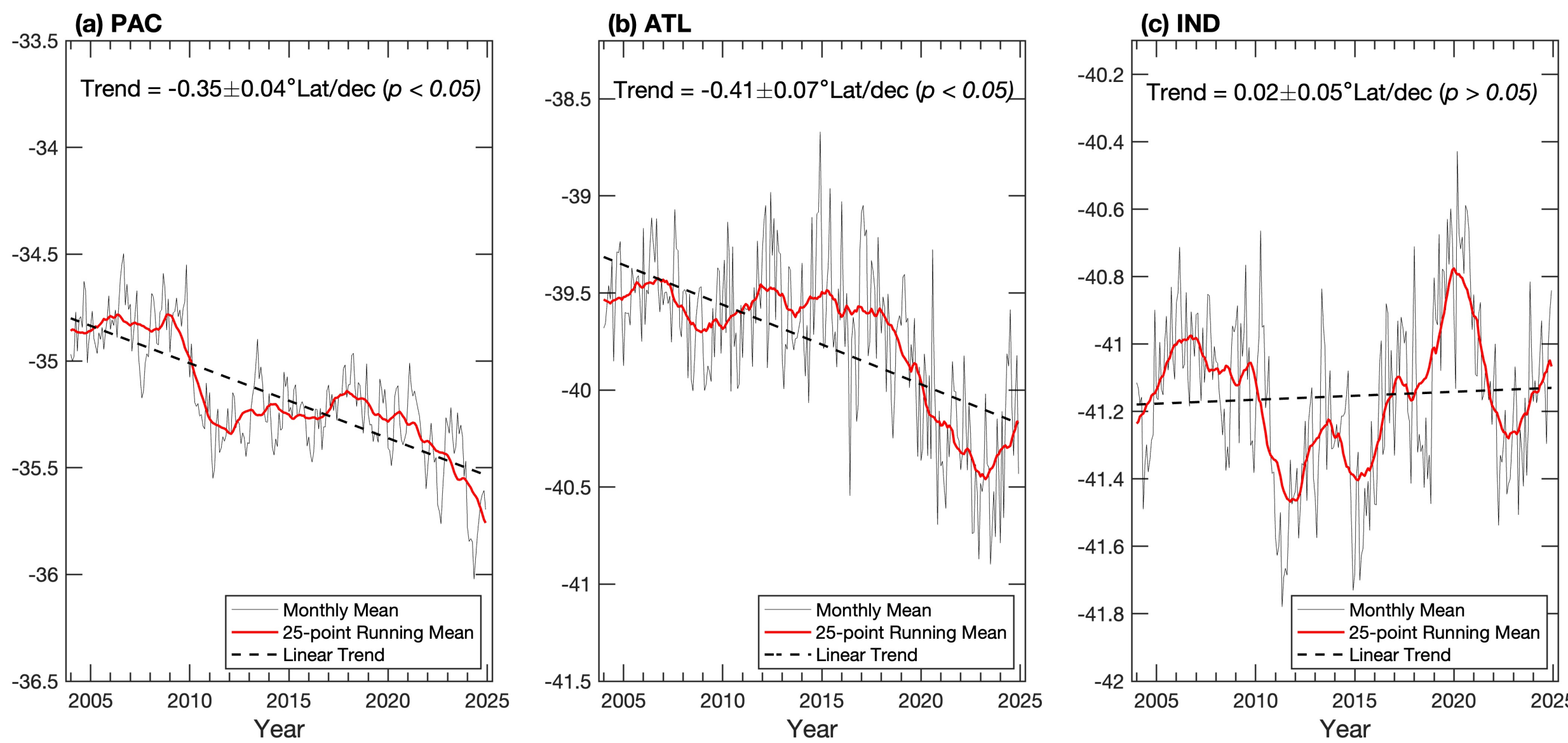

Supplementary Fig. 1 Basin-scale poleward migration of the surface 35 PSS-78 isohaline (S(35)), a proxy for the Subtropical Front. a–c Latitude time series of S(35) in each basin: Pacific (a; PAC, 120°E–85°W), Atlantic (b; ATL, 85°W–20°E) and Indian Ocean (c; IND, 20°E–120°E). In each panel, deseasonalized monthly latitudes are shown as the thin grey line, the 25-month running mean as the thick red line, and the least-squares linear trend (fitted to the unsmoothed, deseasonalized series) as the black dashed line; slopes are reported as ° latitude decade^{-1} ± 95% confidence interval. S(35) represents the surface expression of the poleward expansion of the subtropical salinity maximum (main text Fig. 1). Statistically significant southward migration is detected in the Pacific (−0.35 ± 0.04° decade^{-1}) and Atlantic (−0.41 ± 0.07° decade^{-1}) sectors ($p < 0.05$), consistent with basin-scale gyre expansion, whereas no significant trend is detected in the Indian Ocean sector (+0.02 ± 0.05° decade^{-1}; $p > 0.05$).

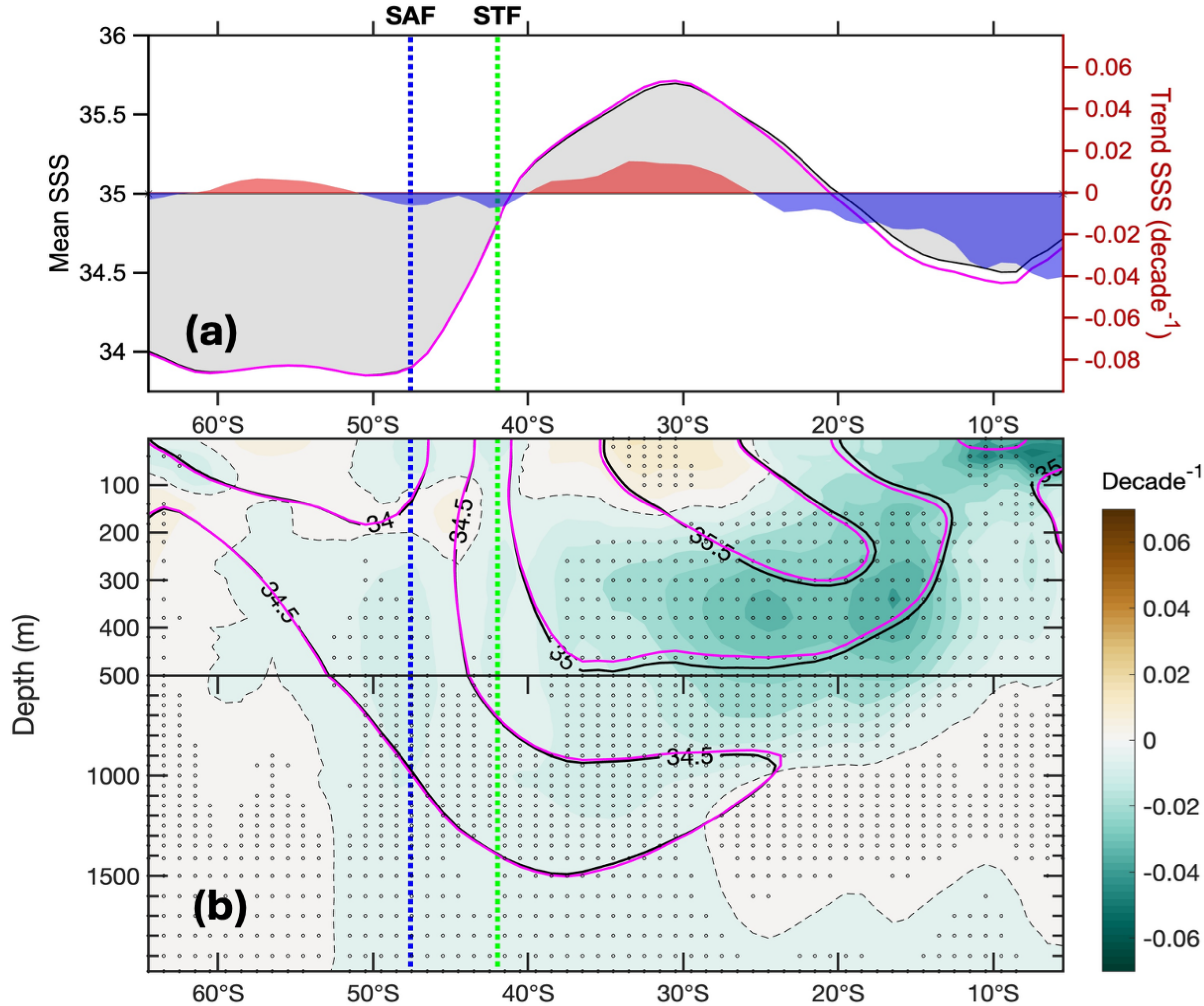

Supplementary Fig. 2 Zonal-mean salinity climatology and trends in the Indian Ocean sector (20°E–120°E). **a** SSS climatology (grey, left axis) and linear trend (shaded envelope, right axis; red, salinification; blue, freshening) versus latitude. Black and magenta curves show period-mean SSS for 2004–2008 and 2020–2024. **b** Salinity trend from the surface to 2,000 m (shading; PSS-78 decade^{-1}), with the upper 500 m on an expanded scale; stippling marks $p < 0.05$. Black and magenta contours show period-mean isohalines (PSS-78) for 2004–2008 and 2020–2024. Vertical dotted lines mark the climatological Subantarctic (SAF, blue) and Subtropical (STF, green) Fronts. Unlike the Pacific and Atlantic sectors (main text Fig. 1), the Indian sector shows no coherent salinification at 40–50°S and no subsurface penetration of positive trends between the STF and SAF, consistent with the absence of significant S(35) migration here (+0.02 ± 0.05° decade^{-1}; $p > 0.05$; Supplementary Fig. 1c). The weaker, less coherent wind-stress-curl trend (Supplementary Fig. 3; Supplementary Note 1) and strong Agulhas Return Current retroflection variability, which produces large interannual frontal displacements, likely obscure any secular gyre-expansion signal.

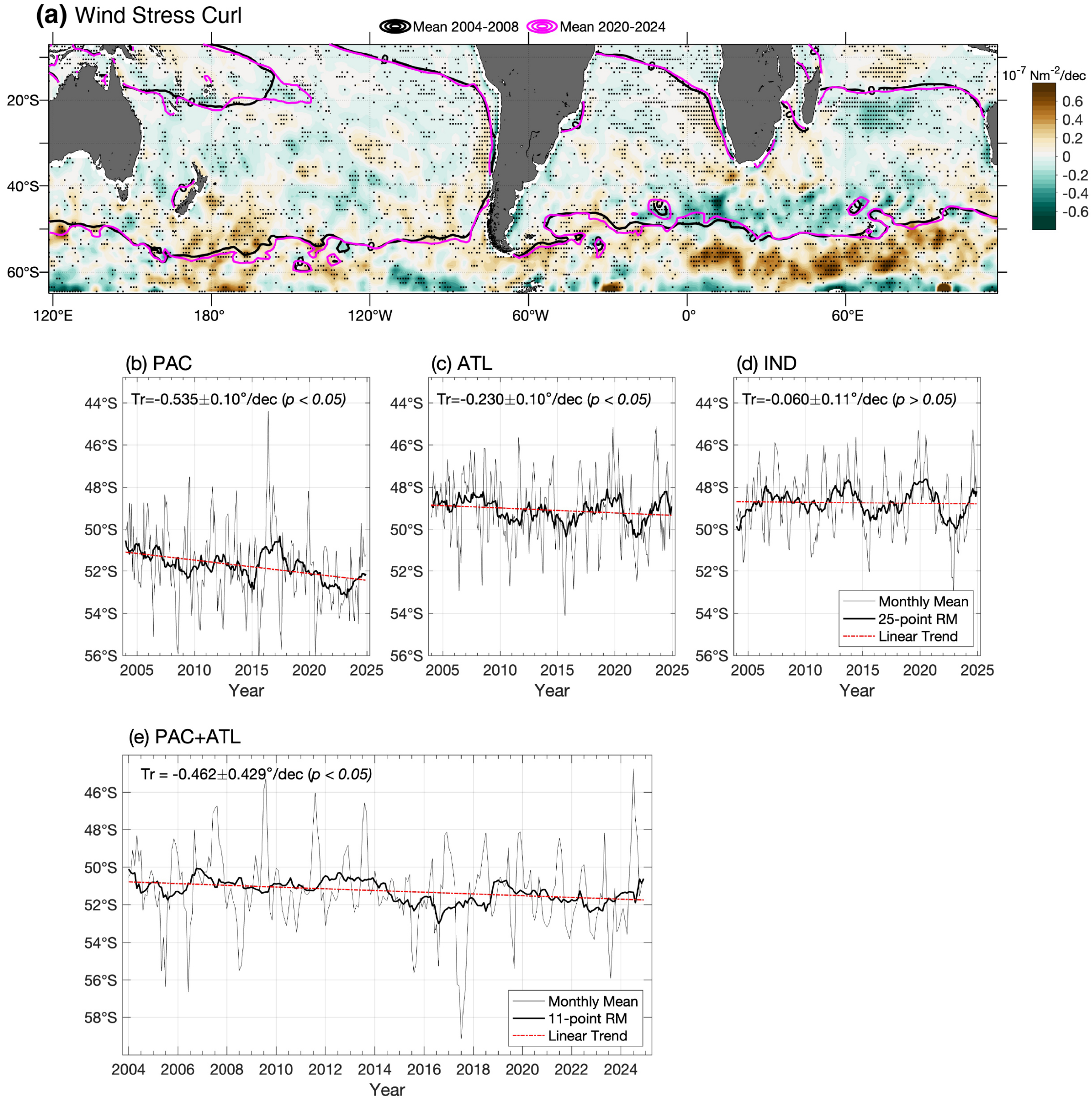

Supplementary Fig. 3 Trends in wind-stress curl and the zero-curl line position across Southern Hemisphere ocean basins. **a** Trend in wind-stress curl from satellite-based reanalysis (shading; 10^{-7} N m^{-2} decade^{-1}), with stippling indicating trends significant at $p < 0.05$. Solid contours show the zero wind-stress-curl line averaged over 2004–2008 (black) and 2020–2024 (magenta); dashed lines indicate the climatological positions of the Subtropical Front (STF, green) and Subantarctic Front (SAF, blue). **b-e** Latitude time series of the zero wind-stress-curl line in the Pacific (b; PAC, 120°E–85°W), Atlantic (c; ATL, 85°W–20°E), Indian (d; IND, 20°E–120°E), and combined Pacific–Atlantic (e; 120°E–20°E) sectors. In each panel, the deseasonalized monthly time series is shown in thin grey, the 25-month running mean in thick black, and the least-squares linear trend (fitted to the deseasonalized, unsmoothed series) as a red dashed line. Trend slopes (Tr) are reported in degrees latitude per decade with 95% confidence intervals. The Pacific and Atlantic show statistically significant poleward migration ($p < 0.05$); the Indian sector does not.

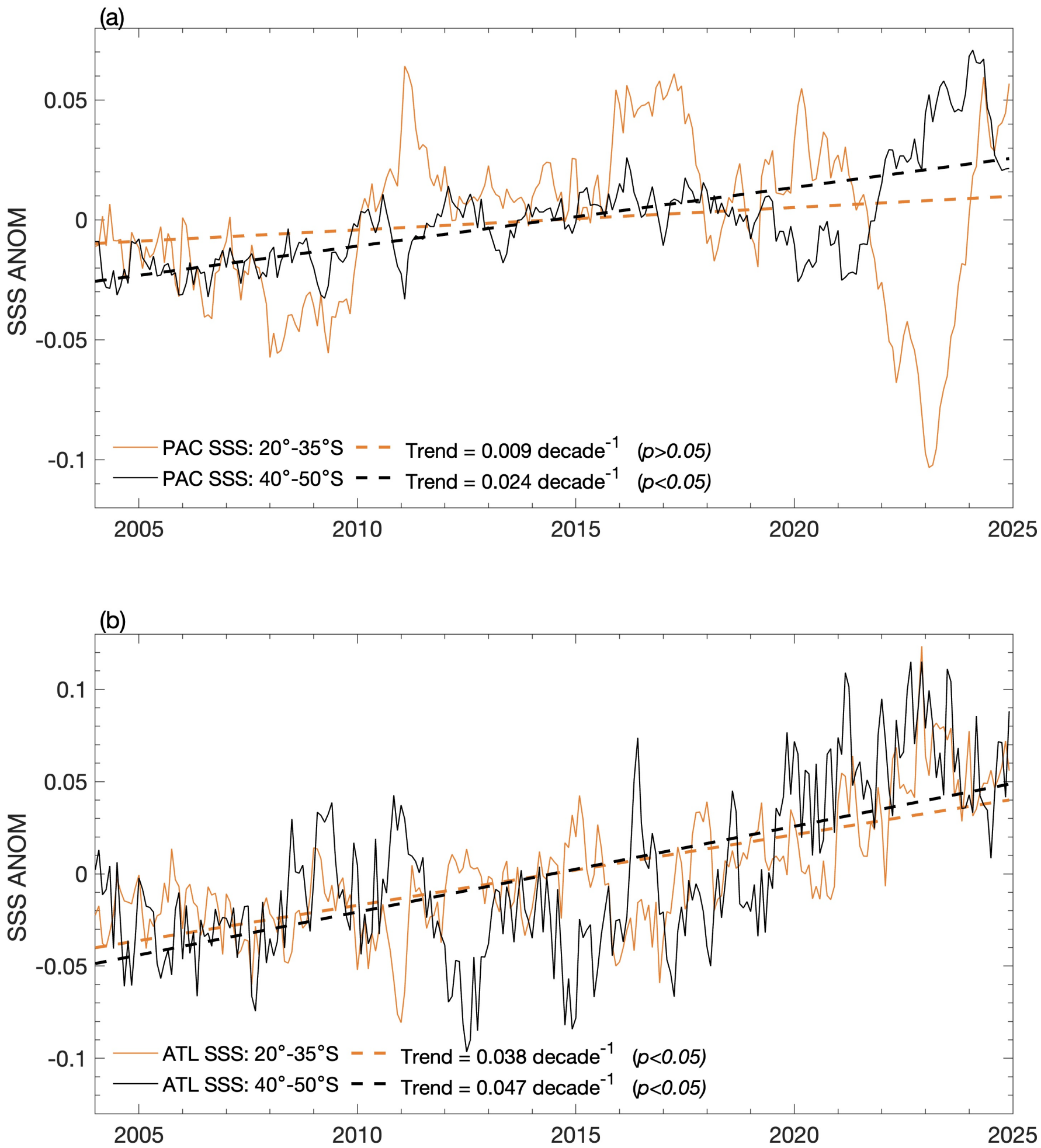

Supplementary Fig. 4 Domain-mean SSS trends for the 40–50°S and 20–35°S latitude bands. (a) South Pacific, (b) South Atlantic. Monthly area-weighted SSS anomalies for the 40–50°S band (black) and the 20–35°S band (orange), with linear trends (dashed; slopes and 95% confidence intervals annotated). In the Pacific, the 40–50°S trend (0.024 decade^{-1}, $p < 0.05$) is significant while the 20–35°S trend (0.009 decade^{-1}, $p > 0.05$) is not. In the Atlantic, both bands show significant positive trends, with the 40–50°S band the stronger of the two (0.047 versus 0.038 decade^{-1}; both $p < 0.05$). The relative magnitude of salinification between bands is therefore basin-dependent, consistent with the basin-specific patterns shown in main text Fig. 2.

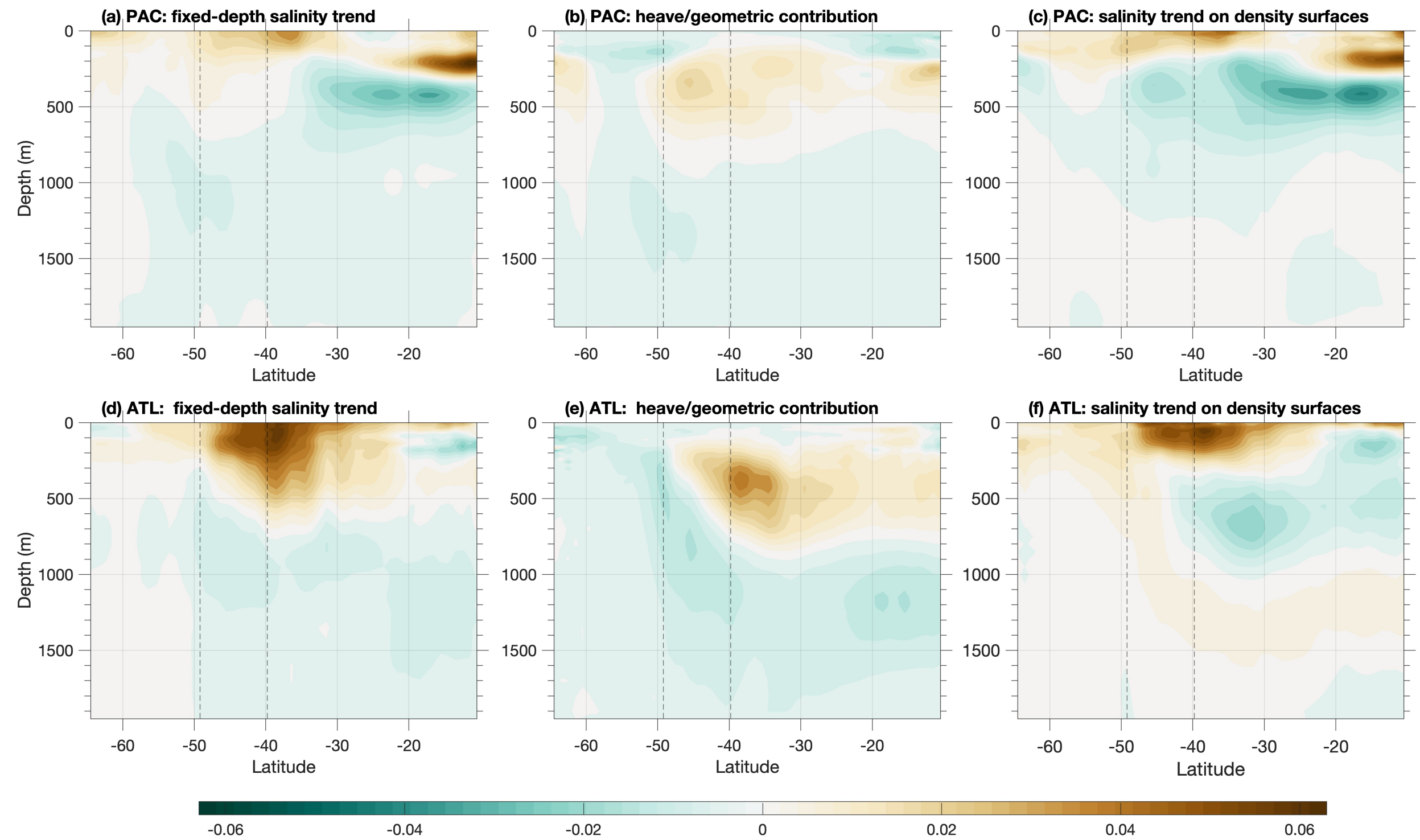

Supplementary Fig. 5 Isopycnal/heave decomposition of the depth–latitude salinity trend (2004–2024). Trends in decade^{-1}. Left column (a,d), total Eulerian salinity trend at fixed depth; middle column (b,e), heave contribution from vertical displacement of density surfaces, $-(\partial S/\partial z)(\partial z_\sigma/\partial t)$; right column (c,f), salinity trend on density surfaces (water-mass change). Top row (a–c), Pacific (PAC; 120°E–85°W); bottom row (d–f), Atlantic (ATL; 85°W–20°E). Dashed vertical lines mark the climatological SAF (left) and STF (right). The fixed-depth trend (a,d) is the sum of the heave (b,e) and density-surface (c,f) components. In the upper 500 m of the 40–50°S band, the heave term reflects wind-driven deepening of subtropical isopycnals and the density-surface term reflects salinification of water masses; both are positive, demonstrating that the subsurface trend is a dynamical signal rather than a purely geometric artefact of poleward-migrating isohalines.

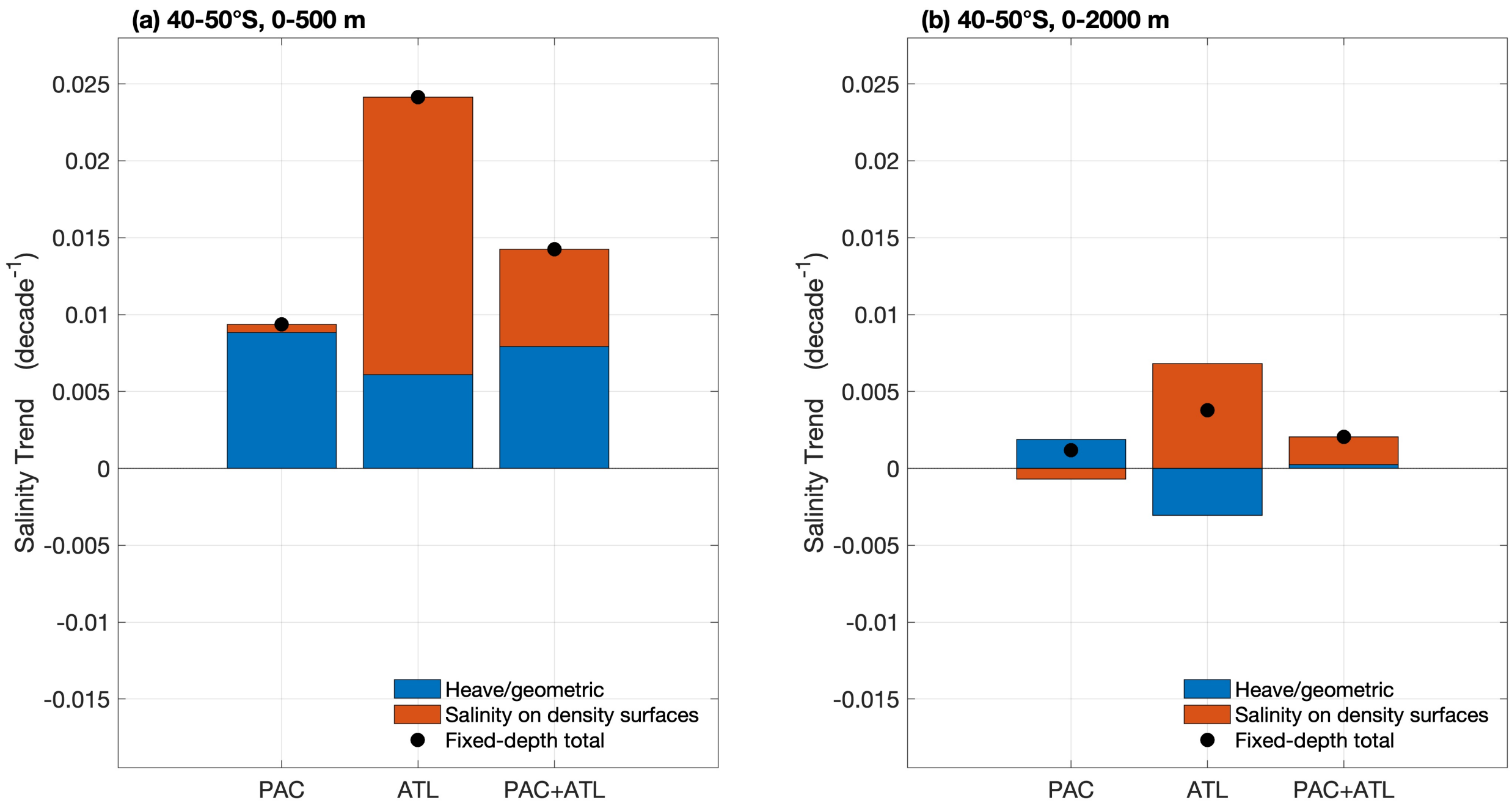

Supplementary Fig. 6 Depth-integrated decomposition of the 40–50°S fixed-depth salinity trend. Trends in decade^{-1}. Heave (blue) and density-surface (orange) contributions to the fixed-depth salinity trend (black dots), integrated over (a) the upper 0–500 m and (b) the full 0–2,000 m, for the Pacific (PAC), Atlantic (ATL), and combined (PAC+ATL) averages. In the upper 500 m (a), both components are positive in all sectors; the combined trend (~0.014 decade^{-1}) comprises a heave and a density-surface contribution, with heave the larger contributor in the Pacific and water-mass change the larger contributor in the Atlantic (~75%). The 0–2,000 m integral (b) is shown for completeness; it is dominated by near-zero, weakly stratified deep layers where the decomposition is poorly constrained and the two components partly cancel, and is therefore not used for interpretation.

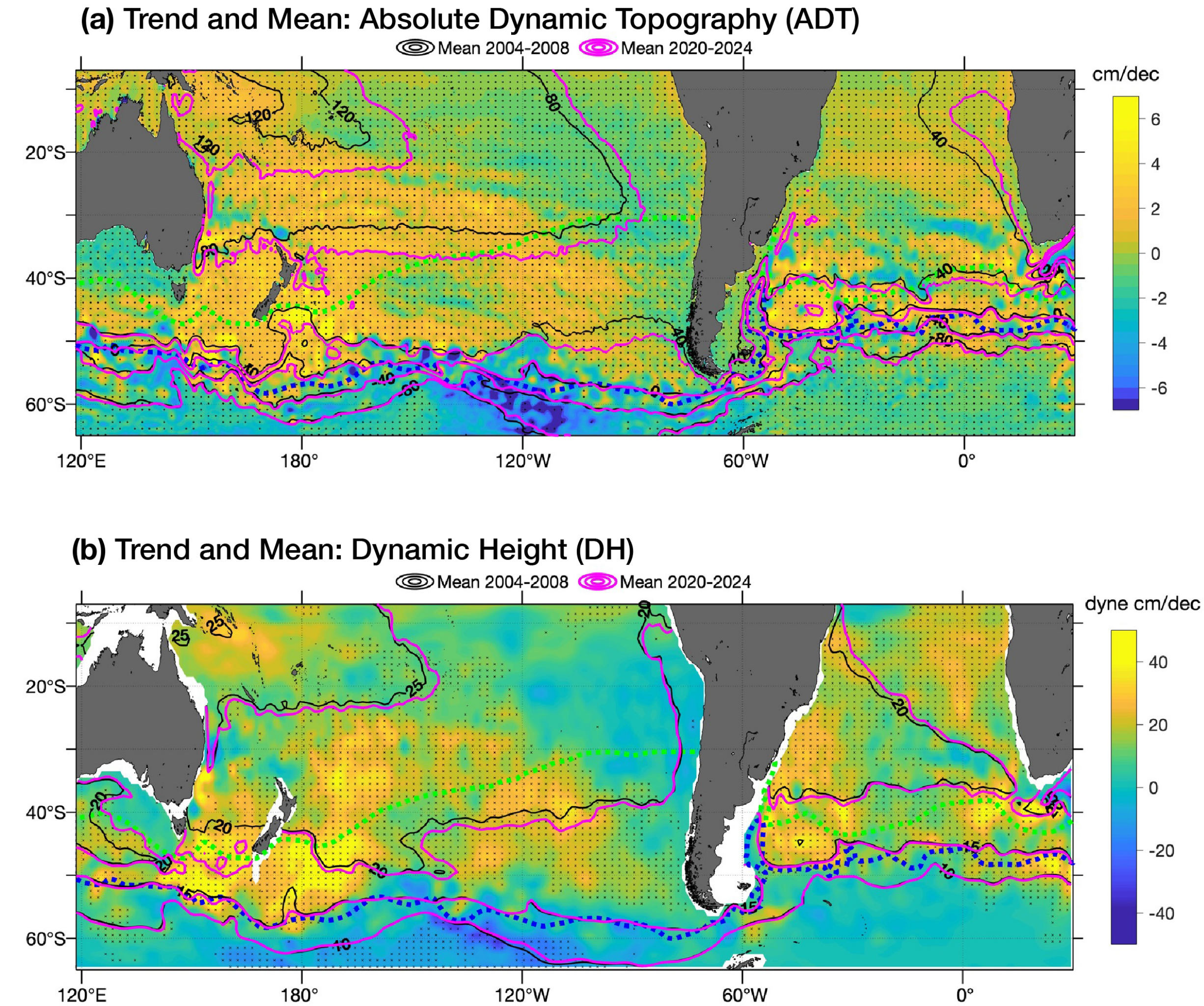

Supplementary Fig. 7 Trends and multidecadal changes in absolute dynamic topography (ADT) and dynamic height (DH) across the Southern Hemisphere. **a** ADT trend from satellite altimetry (shading; cm decade^{-1}), with the global-mean sea-level rise (3.7 cm decade^{-1}) removed. **b** DH trend at the surface relative to 2000 dbar from Argo (shading; dyne cm decade^{-1}). In both panels, stippling indicates trends significant at $p < 0.05$; solid contours show the period-mean fields for 2004–2008 (black) and 2020–2024 (magenta), with values labelled in cm (a) and dyne cm (b); dotted lines mark the climatological positions of the Subtropical Front (STF, green) and Subantarctic Front (SAF, blue). Positive trends are widespread across the subtropical gyres north of the SAF, consistent with a strengthening and/or poleward expansion of the anticyclonic circulation. The poleward displacement of the zero-ADT contour (a) and of the 25 dyne cm DH contour (b) is consistent with the southward migration of S(35) documented in both sectors (Supplementary Fig. 1a,b).

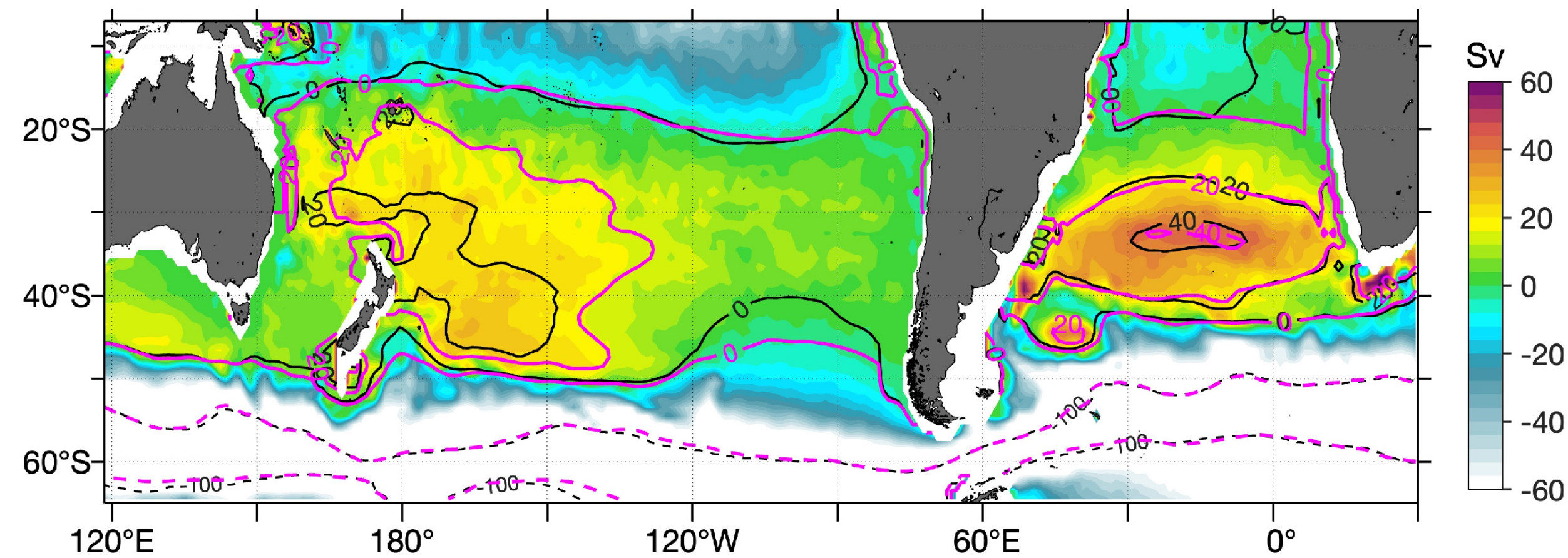

Supplementary Fig. 8 Mean Helmholtz transport streamfunction in the Southern Hemisphere subtropical–subpolar gyres and its multidecadal evolution. Shading shows the time-mean Helmholtz transport streamfunction ψ (Sv) for 2004–2024, providing a transport-based definition of the gyre edge. Overlaid contours compare two five-year climatologies: 2004–2008 (black) and 2020–2024 (magenta), drawn at ψ = −100, 0, +20 and +40 Sv, with negative values dashed. The zero contour delineates the boundary between the subtropical (positive ψ) and subpolar (negative ψ) circulation regimes; comparison of the black and magenta contours reveals the poleward displacement of the gyre edge over the study period, particularly evident in the South Atlantic (~20°W–10°E) and around southeastern Australia. Land is shaded grey.

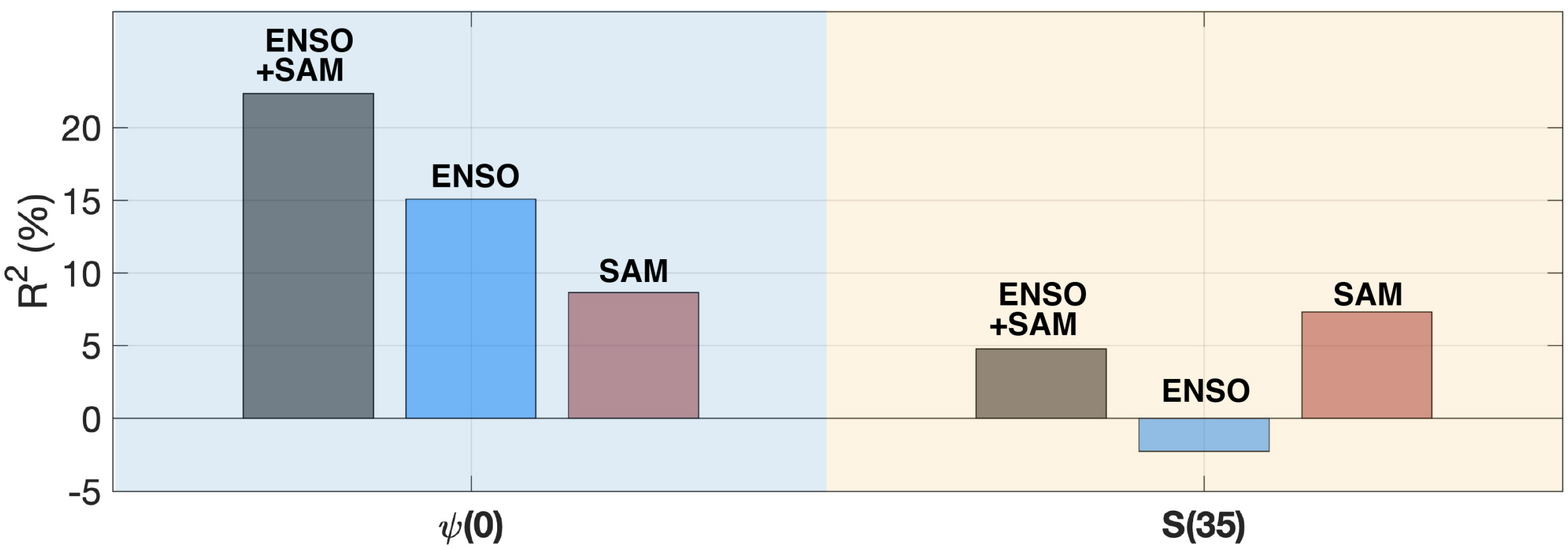

Supplementary Fig. 9 Climate-mode contributions to the poleward migration of ψ(0) and S(35). Variance explained (R^2, %) by linear regressions of the gyre-edge latitude ψ(0) (left, blue panel) and the Subtropical Front proxy latitude S(35) (right, orange panel) onto three predictor sets: joint ENSO + SAM, ENSO-only, and SAM-only (bars from left to right within each panel). For ψ(0), the combined ENSO + SAM model explains ~22% of the interannual variance, with ENSO the larger individual contributor (~15%) and SAM weaker (~9%). For S(35), SAM dominates (~7%), the joint model explains ~5%, and ENSO alone shows negligible (negative-adjusted) skill, indicating that the front's meridional excursions are predominantly SAM-driven. The regression framework fits a linear, additive model to each time series (Methods, Eq. 4), isolating the interannual ENSO and SAM components while preserving the long-term trend (Methods, Eq. 6). Negative adjusted R^2 values arise when a predictor adds no skill beyond what is expected by chance given the degrees of freedom. The approach assumes limited collinearity between the indices and does not resolve nonlinear or lagged interactions, which may account for a portion of the unexplained variance.

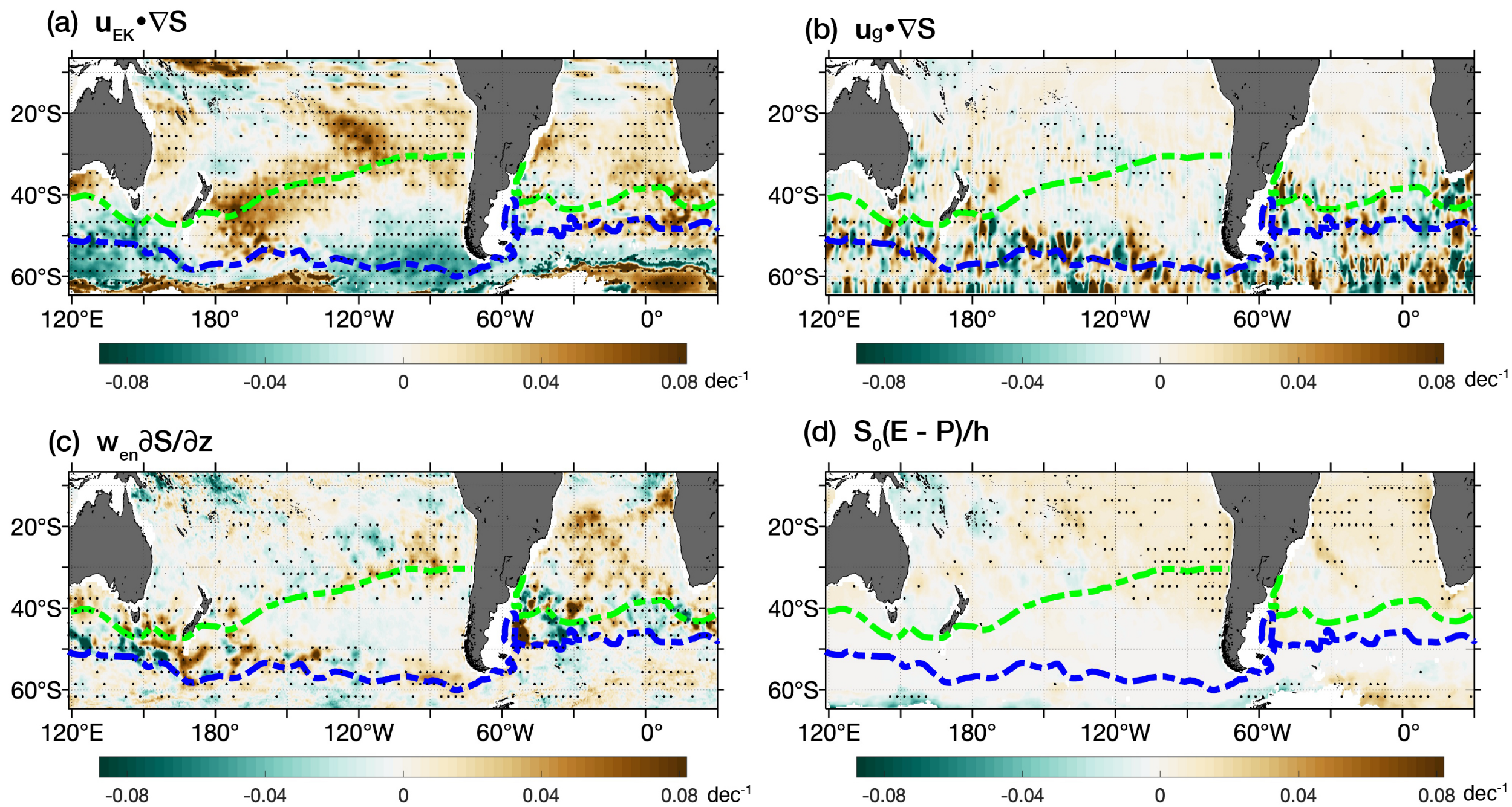

Supplementary Fig. 10 Trend contributions to the mixed-layer salinity budget, 2004–2024. Decadal trends (PSS-78 decade^{-1}) for each budget term, obtained by cumulative integration of the monthly tendencies defined in Methods (Eqs. 8–9): **a** Ekman advection, $\mathbf{u}_{EK} \cdot \nabla S$; **b** geostrophic advection, $\mathbf{u}_g \cdot \nabla S$; **c** entrainment, $w_{en}\, \partial S/\partial z$; and **d** surface freshwater flux, $S_0(E - P)/h$. Horizontal diffusion ($\kappa h \nabla^2 S$) is subdominant and is not shown. Positive values indicate salinification. Dashed contours indicate the climatological positions of the Subtropical Front (STF, green) and Subantarctic Front (SAF, blue).

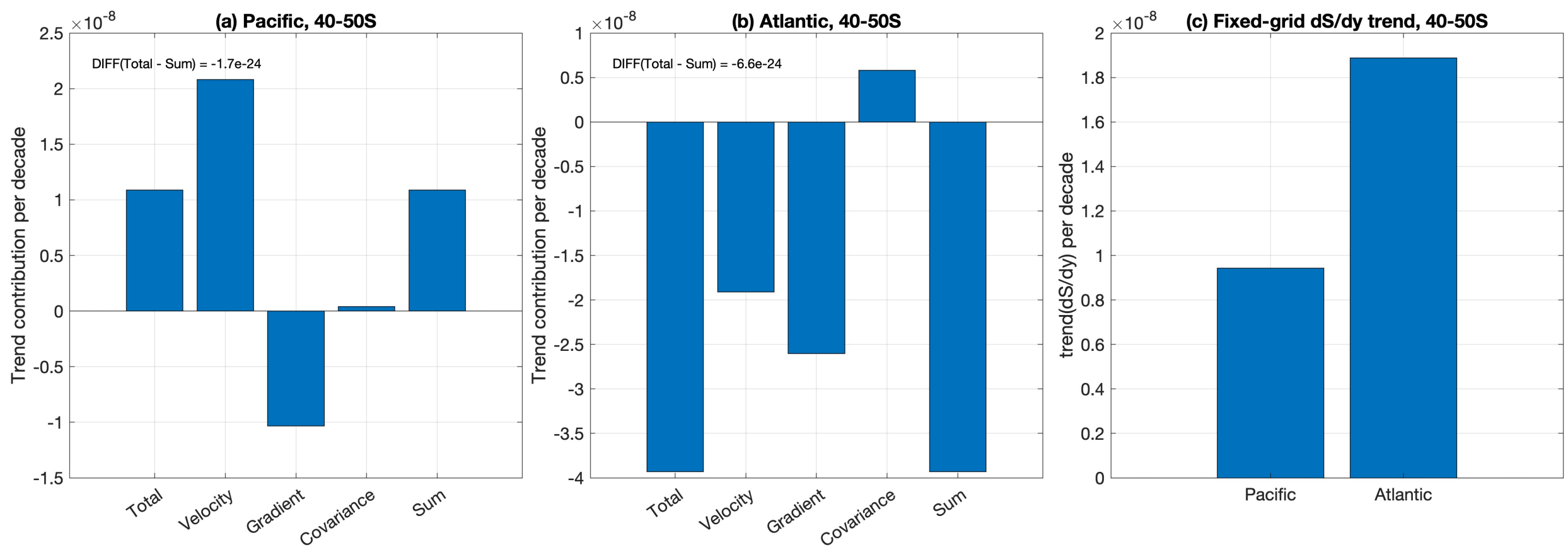

Supplementary Fig. 11 Decomposition of the meridional Ekman salinity tendency trend at 40–50°S (2004–2024). The 2004–2024 linear trend of the meridional Ekman salinity tendency, $T_{EK,y} = -v_{EK}\, \partial S/\partial y$, is decomposed as: trend($T_{EK,y}$) = – [trend(v_{EK}) × $\overline{\partial S/\partial y}$] – [$\overline{v_{EK}}$× trend($\partial S/\partial y$)] + R_{cov}, where overbars denote 2004–2024 temporal means. The first term (Velocity) is the contribution from changes in meridional Ekman transport acting on the mean salinity gradient. The second term (Gradient) is the contribution from changes in the local fixed-grid meridional salinity gradient acted upon by the mean Ekman velocity. The third term, R_{cov} = trend [$-v_{EK}'\,(\partial S/\partial y)'$], is the covariance residual, with primes denoting departures from the monthly mean. **(a)** Pacific sector (120°E–85°W). **(b)** Atlantic sector (85°W–20°E). In both panels, Total is the linear trend of $T_{EK,y}$ computed directly from the full product $-v_{EK}\, \partial S/\partial y$ without decomposition; Sum is the sum of the three decomposed terms (Velocity + Gradient + Covariance). The agreement between Total and Sum (difference < 10^{-23}) verifies closure of the linearized decomposition. **(c)** Linear trend of the fixed-grid meridional salinity gradient $\partial S/\partial y$ averaged over 40–50°S for each basin. Positive values indicate steepening of the northward salinity gradient. All trends are per decade; tendency terms in (a, b) are in decade^{-1} and the gradient trend in (c) is in decade^{-1}.

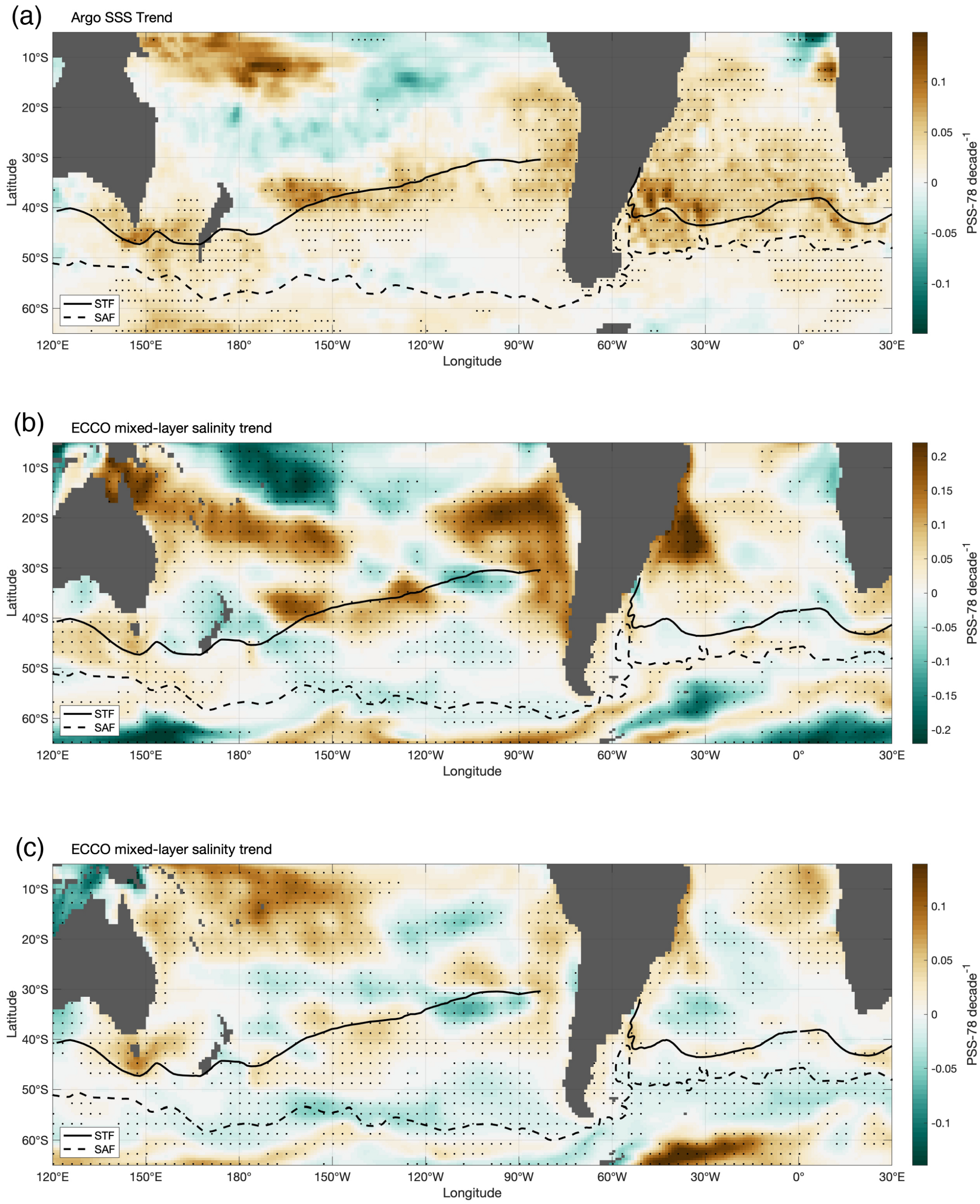

Supplementary Fig. 12 Mixed-layer salinity trends from Argo (2004–2024) and ECCO v4r4 (1992–2017, 2004–2017) across the South Pacific and South Atlantic basins (10°S–65°S), expressed in PSS-78 decade^{-1}. (a) Surface salinity trend from gridded Argo observations over 2004–2024. (b) ECCO mixed-layer salinity trend over 2004–2017, overlapping with part of the Argo period. (c) ECCO mixed-layer salinity trend over the full reanalysis period 1992–2017. Stippling indicates regions where the trend is statistically significant at the 95% confidence level. Solid and dashed black contours denote the climatological positions of the STF and SAF, respectively. Note the differing color scale in (b) relative to (a) and (c).

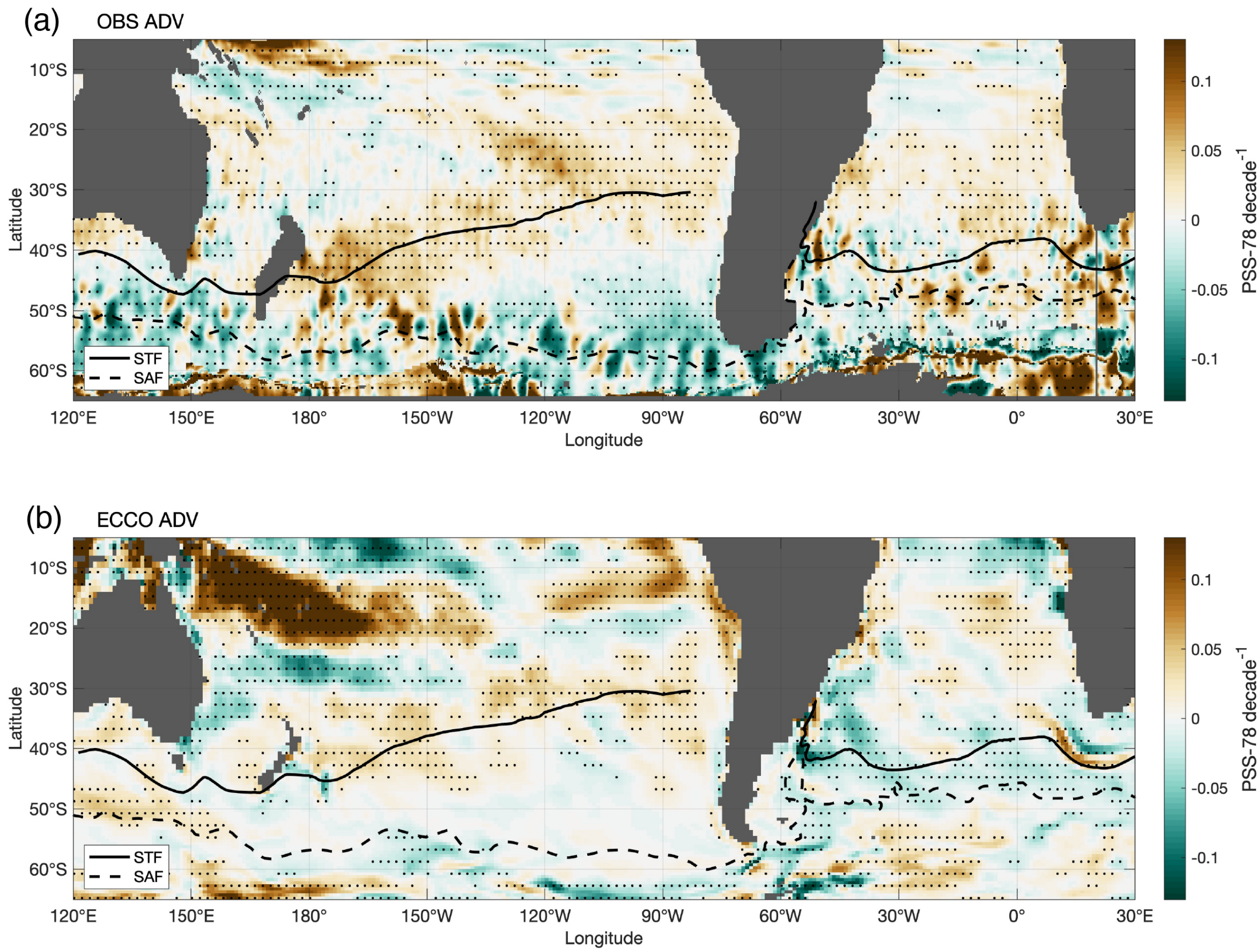

Supplementary Fig. 13 Trends in horizontal advection from the observation-based budget and total advection from ECCO v4r4 budget, expressed in PSS-78 decade^{-1}. **a** Horizontal advection ($-u \cdot \nabla S$, where u combines the geostrophic and Ekman velocities) from the observation-based mixed-layer salinity budget over 2004–2024. **b** Total advection ($ADV_h + ADV_v$) from the closed ECCO mixed-layer salinity budget over 2004–2017.over 2004–2017. In both panels, the trend is derived by linearly fitting the time-integrated (cumulative) budget term (Methods Eqs. 8-10). Stippling indicates regions where the trend is statistically significant at the 95% confidence level. Solid and dashed black contours denote the climatological positions of the STF and SAF, respectively.

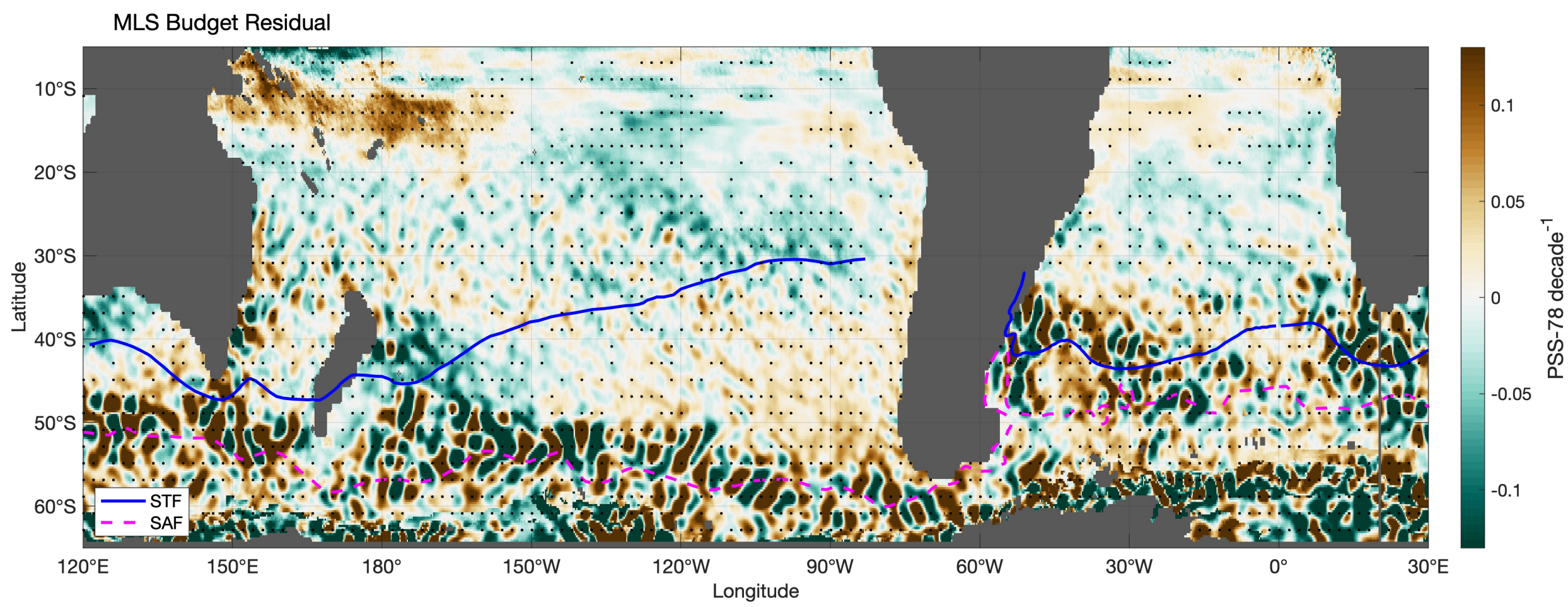

Supplementary Fig. 14 Spatial distribution of the linear trend in the observation-based mixed-layer salinity budget residual. The residual represents the difference between the observed salinity tendency and the sum of the diagnosed budget contributions (surface freshwater flux, horizontal advection, and entrainment; Methods Eq.7). The trend is derived by linearly fitting the time-integrated (cumulative) residual. Stippling indicates regions where the trend is statistically significant at the 95% confidence level. Solid blue and dashed magenta contours denote the climatological positions of the STF and SAF, respectively.

Supplementary References

1. Wang, R., Nan, F., Yu, F. & Wang, B. Subantarctic mode water variations in the three Southern Hemisphere ocean basins during 2004–2019. J. Geophys. Res. Oceans 127, e2021JC017906 (2022).
2. Volkov, D. L. et al. Unprecedented reduction and quick recovery of the South Indian Ocean heat content and sea level in 2014–2018. *Sci. Adv*. **6**, eabc1151 (2020).
3. Beal, L. M., De Ruijter, W. P. M., Biastoch, A., Zahn, R. & SCOR/WCRP/IAPSO Working Group 136. On the role of the Agulhas system in ocean circulation and climate. *Nature* **472**, 429–436 (2011).
4. Biastoch, A., Böning, C. W., Schwarzkopf, F. U. & Lutjeharms, J. R. E. Increase in Agulhas leakage due to poleward shift of Southern Hemisphere westerlies. *Nature* **462**, 495–498 (2009).
5. Forget, G., Campin, J.-M., Heimbach, P., Hill, C. N., Ponte, R. M. & Wunsch, C. ECCO version 4: an integrated framework for non-linear inverse modeling and global ocean state estimation. *Geosci. Model Dev*. **8**, 3071–3104 (2015).
6. Piecuch, C. G. A note on practical evaluation of budgets in ECCO version 4 release 3. https://ecco.jpl.nasa.gov/drive/files/Version4/Release3/doc/evaluating_budgets_in_eccov4r3.pdf (2017).
7. ECCO Consortium, Fukumori, I., Wang, O., Fenty, I., Forget, G., Heimbach, P. & Ponte, R. M. Synopsis of the ECCO central production global ocean and sea-ice state estimate, version 4 release 4. https://ecco.jpl.nasa.gov/drive/files/Version4/Release4/doc/v4r4_synopsis.pdf (2021).
8. Qu, T., Gao, S. & Fukumori, I. What governs the North Atlantic salinity maximum in a global GCM? *Geophys. Res. Lett*. **38**, L07602 (2011).
9. Qu, T., Gao, S., Fukumori, I., Fine, R. A. & Lindstrom, E. J. Origin and pathway of equatorial 13°C water in the Pacific identified by a simulated passive tracer and its adjoint. *J. Phys. Oceanogr*. **39**, 1836–1853 (2013).
10. Gao, S., Qu, T., and Nie, X. Mixed layer salinity budget in the tropical Pacific Ocean estimated by a global GCM, *J. Geophys. Res. Oceans*, **119**, 8255–8270 (2014)
11. Ponte, R. M. & Vinogradova, N. T. An assessment of basic processes controlling mean surface salinity over the global ocean, *Geophys. Res. Lett*., **43**, 7052–7058 (2016).
12. Vinogradova, N. T. & Ponte, R. M. In search of fingerprints of the recent intensification of the ocean water cycle. *J. Clim*. **30**, 5513–5528 (2017).
13. Menemenlis, D. et al. ECCO2: High resolution global ocean and sea ice data synthesis. *Mercator Ocean Q. Newsl.* **31**, 13–21 (2008).

14. Mason, E., Pascual, A., Gaube, P., Ruiz, S., Pelegrí, J. L. & Delepoulle, A. Subregional characterization of mesoscale eddies across the Brazil–Malvinas Confluence. *J. Geophys. Res. Oceans* **122**, 3329–3357 (2017).
