# Supplementary material for: Subtropical gyre expansion causes Southern Ocean salinification contrary to freshening predictions
Source: Nat Commun. 2026 Jul 22;17:8924. doi: 10.1038/s41467-026-75775-2 (PMC13503869; doi:10.1038/s41467-026-75775-2)
Supplement: Supplementary file 2 [file 41467_2026_75775_MOESM2_ESM.pdf]

nature portfolio

Peer Review File

Subtropical gyre expansion causes Southern Ocean salinification contrary to freshening predictions

Corresponding Author: Dr Lisan Yu

This file contains all reviewer reports in order by version, followed by all author rebuttals in order by version.

Attachments originally included by the reviewers as part of their assessment can be found at the end of this file.

Version 0:

Reviewer comments:

Reviewer #1

(Remarks to the Author)
The paper presents significant findings that challenge the prevailing paradigm of ocean salinity changes under climate change.The authors provide compelling observational evidence highlighting the role of dynamic ocean circulation in driving salinification, supported by two decades of high-quality data. The study is generally well structured, and has strong potential to make a significant contribution to oceanography and climate science, as well as for evaluating global climate models.

However, the paper has several notable limitations that need to be addressed before publication. These include limitations in the treatment of Indian Ocean variability, unresolved residuals in the mixed-layer salinity budget, methodological assumptions in trend attribution, and insufficient discussion of observational uncertainty. Clarification and additional quantitative analysis would strengthen the robustness and clarity of the conclusions. Additionally, the methods section could be made more accessible to non-specialist readers.

Major concerns:

1. Limited exploration of Indian Ocean dynamics:
The manuscript notes that salinity trends in the Indian Ocean sector are more variable and patchy compared to the Pacific and Atlantic sectors, yet does not fully explore the mechanisms behind this variability. A deeper dynamical analysis and discussion of the differences of the Indian Ocean from Pacific and Atlantic Oceans would provide a more complete picture of Southern Ocean salinity evolution.

2. Mixed-layer salinity budget issues:
(1) The mixed-layer salinity budget leaves a substantial residual (~34%), attributed to unresolved processes, submesoscale transport, and data uncertainties. However, the residual is not examined in depth, and no clear pathways are proposed for reducing or diagnosing this residual in future work. This weakens confidence in the mechanistic attribution of salinity changes.

(2) Interpretation of $\partial S/\partial t$ trends: The discussion of trends in $\partial S/\partial t$ and its contributing terms is conceptually unclear. If no salinity trend exists, $\partial S/\partial t$ should average to zero. A positive constant $\partial S/\partial t$ implies salinification. Therefore, discussing trends in $\partial S/\partial t$ is not straightforward when interpreting long-term salinity changes. Clarification is needed on why trends in $\partial S/\partial t$ are shown and how they relate to diagnosed salinity trends.

(3) Sign of Ekman Advection: The Ekman advection term (vek $\partial S/\partial y$) should be negative, implying freshening in the SAZ. Increase in $\partial S/\partial y$ would strengthen freshening effect from the Ekman advection. The manuscript should clarify this and physical interpretation used.

3. Uncertainty in Trend Attribution (ENSO and SAM Removal): The study removes ENSO and SAM signals statistically to isolate multidecadal trends. However, the assumption of linear, additive relationships between ENSO/SAM indices and salinity may oversimplify complex climate–ocean interactions. Additionally, the discussion of SAM is somewhat confusing: it is described as important, yet the salinity trend changes little after removing ENSO+SAM signals.

This apparent inconsistency should be clarified.

4. Front and isoline interpretation:
(1) The manuscript frequently uses 40–50°S to represent the SAZ. However, the SAZ varies significantly zonally, in particular, in the Pacific sector, it lies substantially farther south. A fixed latitude band is therefore not a robust approximation, which is clearly demonstrated in Fig.5c.

(2) Constant SSS values are used as proxies for the STF and SAF. However, the salinity associated with these fronts may vary over time. The assumption of constant SSS should be justified or verified.

5. Observational data uncertainty: The study relies heavily on Argo profiles, satellite altimetry, and related datasets. While robust, the manuscript does not sufficiently discuss spatial and temporal coverage gaps, measurement uncertainties, potential biases between datasets, and impacts of these uncertainties on diagnosed trends. A dedicated uncertainty discussion would improve credibility.

6. Broader impacts of salinity changes: The manuscript focuses primarily on salinity trends but gives limited attention to broader implications of the salinification for marine ecosystems, biodiversity, nutrient cycling, carbon sequestration. Expanding this discussion would provide a more impactful perspective.

Minor comments:
L51–54: The roles of the Agulhas Return Current and variability in the Antarctic Circumpolar Current (ACC) are mentioned only briefly. The manuscript would benefit from a clearer explanation of how changes in these currents dynamically influence gyre boundary shifts. In particular, elaborating on the physical mechanisms linking Agulhas Return flow variability and ACC shifts to frontal displacement would strengthen the interpretation.

L124–126 & L278–279: The authors seem to attribute the deepening of S(34) and S(35) along their poleward flanks to dynamical effect. However, this deepening could result from the poleward shift of curved isohalines, producing a geometric effect rather than true vertical signal propagation. The discussion appears to mix Eulerian and Lagrangian perspectives, which may contribute to conceptual ambiguity.

L155-156: Strengthening of gyre does not necessarily induce gyre expansion and shift of the gyre boundary.

L241-249: The authors argue that the salinity gradient sharpens because S(35) moves poleward faster than S(34). However, the difference in displacement appears relatively small. A quantitative estimate of how much this differential shift contributes to overall salinification would strengthen the argument.

L304: remove “25”

L305: what do you mean by “so the net stratification tendency is regional and seasonal”?

Reviewer #2

(Remarks to the Author)
The manuscript presents a compelling analysis of Southern Ocean surface salinity trends during 2004-2024 period, attributing salinification to poleward subtropical gyre expansion, advecting high salinity waters southward, dominating over thermodynamic forcing from the intensifying water cycle. The Helmholtz transport streamfunction is used to objectively track gyre boundaries ($\psi=0$ contour), revealing poleward shifts at –0.49 +/- 0.15°per decade, closely matching S(35) isohaline (tracking the STF) migration of –0.46 +/- 0.04° per decade. This agreement between independent circulation and salinity metrics provides strong evidence that subtropical gyre expansion drives the observed salinification. Finally, mixed-layer salinity budget analysis attributes the 40–50° salinification peak primarily to horizontal advection, while surface freshwater forcing is secondary at these latitudes. The results thus demonstrate that circulation reorganization can dominate regional salinity responses even where net precipitation is increasing.

This is a clear, well written work, providing a strong, multi dataset observationally based analysis with a clear, original, well documented central result, of significance to the field and related fields.

Maybe the weakest part of analysis is the mixed-layer salinity budget analysis with a residual of 34%, which potentially may bias advection attribution. It may be useful to test budget analysis results using an ocean state estimate, such as ECCOv4. Reproducing the regional salinity tendency (Fig. 4) and showing that it can be explained primarily by a sum of certain terms in a rigorously closed ECCO budget, would demonstrate that the mechanisms proposed in the manuscript are dynamically consistent and not an artifact due to a large residual term. It would be useful to show a map of the residual in the supplement, to regionally compare the typical magnitude of the residual relative to the observed salinity trend.

Indian sector patchiness, with alternating regions of salinification and freshening and only intermittent maxima near the STF is noted, but not analyzed in detail. More information provided in the supplement would be valuable, maybe just adding one supplementary figure and one paragraph in the text summarizing what is known in the Indian sector.

Reviewer #3

(Remarks to the Author)
Please find my comments attached.

Version 1:

Reviewer comments:

Reviewer #1

(Remarks to the Author)
The authors have addressed the majority of my previous comments, and I sincerely appreciate their thorough efforts in revising the manuscript. However, there are still a few key points and physical interpretations that require further clarification before the manuscript is finalized.

Comments:

1. In their response and in the text (L150-153), the authors state: "A purely geometric isohaline shift would produce apparent deepening of isohaline contours without a corresponding positive salinity trend at fixed depths." (in response). I have to disagree, a purely geometric shift (like a poleward displacement of curved isohalines) can and will produce a salinity trend at a fixed Eulerian depth. As the sloping structure shifts laterally past a fixed geographic point, a deeper (or shallower) part of the water column is sampled, generating an apparent Eulerian trend without any true water-mass modification.

2. The authors state that over 2004–2024, the meridional salinity gradient ∂S/∂y steepens in the STF–SAF corridor due to faster poleward migration of S(35) relative to S(34), and argue that this steepening progressively reduces this freshening tendency, leading to a positive trend contribution (salinification) from the -vEK ∂S/∂y term. There appears to be a fundamental algebraic and physical contradiction in this logic that needs to be resolved: if the front sharpens and the gradient steepens as described, the magnitude of ∂S/∂y increases. Mathematically, multiplying a larger positive gradient by a positive vEK and a negative sign must result in a larger negative value, which means enhanced freshening, not salinification.

3. I find the authors' justification regarding the frontal definitions contradictory. The text states that S(34) and S(35) are not used as constant frontal proxies, but then asserts that these exact values are assumed to be representative of the SAF and STF across the entire 20-year period. This is a contradiction in terms. If you define a moving front by a fixed salinity value, you are using a constant salinity proxy. Please revise the text to clearly acknowledge that you are tracking specific isohalines as proxies for the fronts, and clarify how you have verified that a broad-scale salinity trend hasn't artificially artificially shifted these isohalines away from the true dynamical fronts (i.e., the maximum gradients).

(Remarks on code availability)

Reviewer #2

(Remarks to the Author)
The revised manuscript satisfactorily addresses the major concerns raised in my previous review, especially the careful analysis of the mixed-layer salinity budget, discussion of the residual, and the attribution of salinification to horizontal advection. The added ECCO-based salinity budget analysis, clearer budget methodology, improved ENSO-SAM explanation, and more detailed treatment of the Indian sector and observational uncertainty strengthen the manuscript and support the main conclusions. A few very minor issues are noted below.

Minor comments
(line numbers refer to the tracked changes version of the text)
1. l. 70: xpanded -> expanded
2. l. 70–71: positive TREND in the Southern Annular Mode (SAM) TREND
3. l. 77: density-drivenN
4. standardize unit formatting, e.g. decade-1 vs. $decade^{-1}$
5. l. 701- 55 – superscript
6. l. 767 – 56, 57 – superscript
7. l. 783 – 34 – superscript
8. l. 948. "Error! Reference source not found"?
9. l. 1288. "the Zenodo repository at xxx"

(Remarks on code availability)

Reviewer #3

(Remarks to the Author)
I appreciate the authors' thoughtful responses to my comments and the substantial revisions made throughout the manuscript. I found the review process highly constructive, and I believe the manuscript has significantly improved in response to the comments from all reviewers. I now support publication.

I only have two remaining minor comments for the authors to consider:

(1) Minor terminology refinement

The concern regarding the contrast between "dynamic ocean processes" and "atmospheric forcing" has been satisfactorily addressed. I only note that the revised terminology ("thermohaline forcing") occasionally reads somewhat unconventional in this context, and the authors may wish to consider whether terms such as "buoyancy forcing" or "surface freshwater forcing" would provide slightly greater physical clarity. In particular, expressions such as "thermohaline freshwater forcing" (Line 31) feel somewhat awkward conceptually.

(2) Remaining concern regarding the description of salinification spatial structure

Concerning the authors' response to my comment associated with Lines 192–194 of the originally submitted manuscript, specifically their item (iii):

"Domain-mean SSS trends averaged over 40–50°S and 20–35°S have been computed. In the Pacific, the two bands show comparable trend magnitudes, consistent with the reviewer's observation; in the Atlantic, the 40–50°S band trend is stronger. These values have been noted in the text."

I was unable to identify where these values were incorporated into the revised manuscript. Since this diagnostic was introduced as supporting evidence for the revised interpretation, it would be helpful either to briefly report the values in the manuscript or to include the corresponding time series/trends in a supplementary figure.

I appreciate the authors' efforts to revise the wording and avoid implying an overly strong magnitude hierarchy between the 40–50°S and 20–35°S bands. However, I still find that the revised phrasing ("salinification maximum" vs. "secondary salinification") somewhat suggests a robust spatial hierarchy that is not always clearly evident from Fig. 1 and Fig. 4a, particularly given the pronounced longitudinal heterogeneity and the substantial subtropical salinification visible across broad portions of both the Pacific and Atlantic sectors.

More generally, I wonder whether the spatial patterns might be more robustly and simply described relative to the STF/front location itself, rather than through a stronger-versus-secondary hierarchy between fixed latitude bands. To my eye, the figures most consistently support a salinification maximum organized around the STF region and its displacement, whereas the relative magnitude contrast between 40–50°S and 20–35°S appears more longitudinally variable.

I also note that a frontal/STF-centered framing seems more directly aligned with the dynamical interpretation proposed throughout the manuscript (e.g., gyre expansion, STF displacement, frontal migration, and associated advection), and may therefore connect the spatial description more naturally to the underlying mechanism discussed by the authors.

(Remarks on code availability)

Open Access This Peer Review File is licensed under a Creative Commons Attribution 4.0 International License, which permits use, sharing, adaptation, distribution and reproduction in any medium or format, as long as you give appropriate credit to the original author(s) and the source, provide a link to the Creative Commons license, and indicate if changes were made.
In cases where reviewers are anonymous, credit should be given to 'Anonymous Referee' and the source.
The images or other third party material in this Peer Review File are included in the article's Creative Commons license, unless indicated otherwise in a credit line to the material. If material is not included in the article's Creative Commons license and your intended use is not permitted by statutory regulation or exceeds the permitted use, you will need to obtain permission directly from the copyright holder.
To view a copy of this license, visit https://creativecommons.org/licenses/by/4.0/

Response to Reviewer 1

The paper presents significant findings that challenge the prevailing paradigm of ocean salinity changes under climate change. The authors provide compelling observational evidence highlighting the role of dynamic ocean circulation in driving salinification, supported by two decades of high-quality data. The study is generally well structured, and has strong potential to make a significant contribution to oceanography and climate science, as well as for evaluating global climate models.

However, the paper has several notable limitations that need to be addressed before publication. These include limitations in the treatment of Indian Ocean variability, unresolved residuals in the mixed-layer salinity budget, methodological assumptions in trend attribution, and insufficient discussion of observational uncertainty. Clarification and additional quantitative analysis would strengthen the robustness and clarity of the conclusions. Additionally, the methods section could be made more accessible to non-specialist readers.

We thank the reviewer for the positive assessment and for the detailed and constructive We thank the reviewer for the positive assessment of our manuscript and for the thoughtful, detailed, and constructive comments. The review has been highly valuable in improving the clarity and overall presentation of the manuscript. We have addressed all major concerns in the revised manuscript through additional analyses, clarifications, and revisions to the text, as detailed below. Reviewer comments are reproduced in italicized blue text, and our responses are presented in plain text.

Major concerns:

1. Limited exploration of Indian Ocean dynamics:
The manuscript notes that salinity trends in the Indian Ocean sector are more variable and patchy compared to the Pacific and Atlantic sectors, yet does not fully explore the mechanisms behind this variability. A deeper dynamical analysis and discussion of the differences of the Indian Ocean from Pacific and Atlantic Oceans would provide a more complete picture of Southern Ocean salinity evolution.

We thank the reviewer for this suggestion. In response, we have expanded both the main text and the Supplementary Information to describe the Indian sector contrast more explicitly.

Our analyses indicate that the Indian sector differs from the Pacific and Atlantic in several respects. First, the zonal-mean S(35) latitude does not exhibit a statistically significant poleward migration in the Indian sector ($-0.02 \pm 0.05°$ decade^{-1}, $p > 0.05$; Supplementary Fig. 1c), whereas significant southward trends are evident in the Pacific and Atlantic ($-0.35 \pm 0.04°$ decade^{-1} and $-0.41 \pm 0.07°$ decade^{-1}, respectively; both $p < 0.05$). Second, the sea-surface salinity trend map (Fig. 1) shows a patchier pattern in the Indian sector STF–SAF corridor, with alternating regions of salinification and freshening, rather than the more coherent salinification belt seen in the Pacific and Atlantic. Third, the depth–latitude salinity trend section (Supplementary Fig. 2) does not show a comparably coherent subsurface salinification signal in

the 40–50°S band, nor a clear penetration of positive trends to ~500 m, in contrast to the Pacific and Atlantic (Fig. 2), where the subtropical high-salinity layer extends continuously into the SAZ.

We further include a brief discussion of factors that may contribute to this contrast. In particular, the wind-stress-curl trend over the southern Indian Ocean appears weaker and less spatially organized than in the Pacific and Atlantic (Supplementary Fig. 3). This may in part reflect modulation by remote ENSO forcing through the Indonesian Throughflow and associated atmospheric teleconnections linked to the Walker circulation, which may weaken the correspondence between local curl trends and the longer-term westerly trend (Wang et al.; Volkov et al. 2020). In addition, the position of the STF in the Indian sector is strongly influenced by the Agulhas Return Current retroflection geometry, introducing substantial interannual-to-decadal frontal variability that may obscure an underlying secular gyre-expansion signal (Beal et al. 2011; Biastoch et al. 2009).

These additions are documented in Supplementary Note 1 and Supplementary Fig. 1-3, and a summary paragraph with supporting references has been added to the main-text Results section ("Surface salinity trends and associated vertical structure").

2. Mixed-layer salinity budget issues:
(1) The mixed-layer salinity budget leaves a substantial residual (~34%), attributed to unresolved processes, submesoscale transport, and data uncertainties. However, the residual is not examined in depth, and no clear pathways are proposed for reducing or diagnosing this residual in future work. This weakens confidence in the mechanistic attribution of salinity changes.

We thank the reviewer for this comment. We agree that the 34% trend-share residual warrants closer examination, and we have addressed this concern through new analyses presented in Supplementary Note 2, Supplementary Table 1, and Supplementary Figures 8–10.

We implemented an independent mixed-layer salinity budget from the ECCO v4r4 state estimate, following the framework of Piecuch (2017), the ECCO v4r4 budget documentation (ECCO Consortium et al., 2021), and previous studies (Qu et al. 2011; 2013; Gao et al. 2014; and other), including the nonlinear free-surface volume adjustment. The budget was reconstructed on the native LLC90 grid, regridded to a common 0.5° grid, and integrated as cumulative anomaly contributions over two periods within the 26-year ECCO record: 1992–2017 (the full reanalysis period) and 2004–2017 (overlapping with part of the Argo record analyzed in the main text). Averaged over the South Pacific and South Atlantic (120°E–30°E, 65°S–5°S), the ECCO budget achieves near-complete closure, with a residual of approximately 0.003 PSS-78 yr^{-1}, less than 1% of the total magnitude of the physical tendency terms in both periods (Supplementary Table 1).

Reproduction of the regional salinity tendency is shown in Supplementary Fig. 8, where we compare the Argo sea surface salinity trend over 2004–2024 with the ECCO mixed-layer salinity trend over 2004–2017 and 1992–2017. We find that ECCO reproduces the broad structure of the observed Argo salinity trend, with the closest agreement along the Subtropical Front (STF) in the

South Pacific. In the South Atlantic, the basin-scale salinification seen in Argo is less well captured. This Pacific–Atlantic contrast is consistent with the expected skill of ECCO v4r4 in basins dominated by mesoscale eddy activity: the LLC90 grid (~1° at the equator, ~40 km at high latitudes) parameterizes rather than resolves mesoscale eddy fluxes (Forget et al., 2015), a known limitation in regions of energetic boundary currents (Menemenlis et al., 2008) such as the Brazil–Malvinas Confluence and Agulhas Return Current (Mason et al., 2017). ECCO therefore supports the advective attribution in the Pacific, where the modeled salinification is captured, while the Atlantic comparison is limited by the spatial resolution of both ECCO and the Argo gridded product in this eddy-rich basin.

In Supplementary Fig. 9, we compare the trend in horizontal advection from the observation-based budget ($-u\cdot\nabla S$, represented by the Ekman and geostrophic components $u_{EK} + u_g$; Methods Eq. 7) with the trend in ECCO total advection ($ADV_h + ADV_v$). Although the two budgets are constructed differently, we find that both identify the same coherent salinifying advective trend along the Pacific STF, collocated with the climatological STF position and with the observed salinity trend in Supplementary Fig. 8. This convergence supports the dynamical consistency of the advective mechanism proposed in the main text.

We further map the spatial structure of the observation-based residual (Supplementary Fig. 10). As suggested by the reviewer, we mapped the trend in the observation-based budget residual ($C\varepsilon$) to enable a regional comparison with the observed salinity trend. Supplementary Fig. 10 shows that the residual trend is small and spatially patchy across most of the domain, and is particularly modest across the Pacific STF salinification band where the advective attribution is centered. Organized residual trends are confined to the eddy-rich frontal regions of the Subantarctic Front and the western South Atlantic, consistent with the spatial scope of a large-scale, time-mean advective decomposition. The Pacific STF salinification band is not collocated with these organized features, supporting the robustness of the diagnosed advective contribution.

A forward-looking statement has been added to the Discussion. It reads: "Reducing this residual will require mixed-layer budget terms diagnosed at sub-monthly intervals to resolve the mixed-layer-depth–flux covariance and entrainment rectification, and finer spatial resolution to explicitly represent submesoscale and mesoscale lateral stirring unresolved by the gridded observational products."

(2) Interpretation of ∂S/∂t trends: The discussion of trends in ∂S/∂t and its contributing terms is conceptually unclear. If no salinity trend exists, ∂S/∂t should average to zero. A positive constant ∂S/∂t implies salinification. Therefore, discussing trends in ∂S/∂t is not straightforward when interpreting long-term salinity changes. Clarification is needed on why trends in ∂S/∂t are shown and how they relate to diagnosed salinity trends.

We agree that this distinction warrants clarification. A non-zero positive time-mean ∂S/∂t indicates salinification, whereas a trend in ∂S/∂t reflects a change in that tendency rather than the salinity trend itself.

Our objective was not to diagnose acceleration in salinity change, but to attribute the linear salinity trend to the individual budget terms. Following the cumulative integration methodology

(Methods, Eqs. 8–9), each monthly budget term Ti(t) is integrated cumulatively in time to yield the accumulated salinity change ΔSi(t), and the decadal trend contribution Ci is the least-squares linear trend of ΔSi(t) scaled to per-decade units. The resulting Ci has units of PSS-78 decade^{-1}, directly comparable to the observed salinity trends in Figs. 1–2. We have revised the text, updated all figure axis labels to PSS-78 decade^{-1}, and added the cumulative integration and trend contribution decomposition methodology to the Methods (Eqs. 8–10).

(3) Sign of Ekman Advection: The Ekman advection term (vek ∂S/∂y) should be negative, implying freshening in the SAZ. Increase in ∂S/∂y would strengthen freshening effect from the Ekman advection. The manuscript should clarify this and physical interpretation used.

There is an important distinction between the climatological mean Ekman advection and its decadal trend, which the original manuscript did not make sufficiently clear. We have added a clarifying passage to the Results that explicitly separates the two.

The budget equation contains a minus sign in front of the advection terms:

$$\partial S/\partial t = -(u_{EK} + u_g)\cdot\nabla S + S_0(E\text{–}P)/h + w_{en}/h\ (Sb - S) + \kappa_h\nabla^2 S + \varepsilon$$

In the SAZ, the meridional Ekman velocity v_{EK} is directed northward (equatorward Ekman transport driven by the Southern Ocean westerlies) and the meridional salinity gradient ∂S/∂y is positive (salinity increases northward toward the subtropical gyre). The climatological Ekman contribution to the salinity tendency is therefore:

$$-v_{EK}\cdot\partial S/\partial y = -(+)(+) < 0, \text{ indicating } \textbf{freshening}$$

The budget decomposition in Fig. 4d and Supplementary Fig. S7a, however, shows the *decadal trend contribution* of each term, not the climatological mean. Over 2004–2024, the meridional salinity gradient ∂S/∂y steepens in the STF–SAF corridor because S(35) migrates poleward faster than S(34), compressing the SAZ and sharpening the front. This steepening progressively reduces the magnitude of the climatological freshening flux. The decadal trend contribution of $-v_{EK}\cdot\partial S/\partial y$ positive, representing reduced freshening, that is, salinification in a trend sense.

The revised passage in the Results ("Mixed-layer salinity budget and dominant contributions") reads as follows:

"In the climatological mean, Ekman advection freshens the SAZ: northward Ekman transport carries relatively fresh subantarctic surface waters equatorward across the northward-increasing salinity gradient, so that $-v_{EK}\ \partial S/\partial y < 0$. Over 2004–2024, however, the poleward steepening of the meridional salinity gradient, driven by the faster poleward migration of S(35) relative to S(34), progressively reduces this freshening tendency. The Ekman advection trend contribution C_{EK} is therefore positive at 40–50°S, reflecting a weakening of the mean freshening flux rather than direct salinification (Supplementary Fig. 7a)."

3. Uncertainty in Trend Attribution (ENSO and SAM Removal): The study removes ENSO and SAM signals statistically to isolate multidecadal trends. However, the assumption of linear, additive relationships between ENSO/SAM indices and salinity may oversimplify complex

climate–ocean interactions. Additionally, the discussion of SAM is somewhat confusing: it is described as important, yet the salinity trend changes little after removing ENSO+SAM signals. This apparent inconsistency should be clarified.

The relevant paragraph has been revised accordingly, and each point is addressed below.

(1) On the linear additive assumption. The removal procedure fits a linear, additive model at each grid cell (Methods, Eq. 4):

$$y_s(t) = \alpha_s + \beta_{s,ENSO}\, \tilde{X}_{ENSO}(t) + \beta_{s,SAM}\, \tilde{X}_{SAM}(t) + \varepsilon_s(t)$$

where $\tilde{X}_{ENSO}$ and $\tilde{X}_{SAM}$ are standardized indices and parameters are estimated by ordinary least squares. The adjusted field retains the long-term mean while removing the fitted interannual ENSO and SAM components (Methods, Eq. 6). We agree that this framework assumes linearity and additivity, as nonlinear interactions, such as ENSO modulating SAM strength through the atmospheric bridge, or lagged oceanic integration of wind anomalies, are not captured. We have added a qualifying sentence acknowledging this limitation and noting that it applies to the interannual decomposition, not to the primary result. The robustness of the residual secular trend is confirmed by a moving-block bootstrap that resamples consecutive months to preserve temporal dependence (block length $\approx 1.5 \times N^{1/3}$ months; Methods), and 95% confidence intervals are reported for all trend estimates. The persistent ~0.4° decade^{-1} residual trend is significant across all bootstrap realizations, showing that the conclusion is not sensitive to the linearity assumption.

(2) On the apparent SAM inconsistency. The apparent inconsistency arises because SAM operates at two distinct timescales that were not clearly distinguished in the original manuscript. At interannual timescales, SAM explains ~9% of $\psi(0)$ variance and ~7% of S(35) variance (Supplementary Fig. 6). This is the component isolated and removed by the regression. Its removal produces the 10–15% reduction in trend magnitude seen in Fig. 3d.

At multidecadal timescales, SAM has itself trended positively over 2004–2024 as part of the long-term poleward intensification of the Southern Hemisphere westerlies. The regression procedure explicitly preserves the long-term mean (Methods, Eq. 6), because it removes interannual fluctuations around the mean, not the secular trend. The positive SAM trend is therefore retained in the adjusted time series and contributes to the persistent ~0.4° decade^{-1} residual drift after ENSO+SAM removal.

SAM is important at both timescales, as a source of interannual modulation of gyre-edge and frontal positions, and as the driver of the multidecadal atmospheric reorganization underlying the secular migration. The small change in trend after removal (10–15%) is not inconsistent with SAM being important, as it reflects the fact that the regression isolates only its interannual component. The secular SAM contribution is embedded in the residual drift and is corroborated by the concurrent poleward shift of the zero wind-stress-curl latitude (−0.46 ± 0.43° decade^{-1}; Supplementary Fig. 3e). We have revised the manuscript (last paragraph under "Gyre migration from the Helmholtz transport streamfunction (ψ)") to make this two-timescale distinction explicit.

4. Front and isoline interpretation:
(1) The manuscript frequently uses 40–50°S to represent the SAZ. However, the SAZ varies significantly zonally, in particular, in the Pacific sector, it lies substantially farther south. A fixed latitude band is therefore not a robust approximation, which is clearly demonstrated in Fig.5c.
(2) Constant SSS values are used as proxies for the STF and SAF. However, the salinity associated with these fronts may vary over time. The assumption of constant SSS should be justified or verified.

We thank the reviewer for these comments. Each point is addressed in turn, with corresponding revisions made to the manuscript ("Why salinification peaks at 40–50°S").

(1) On the fixed latitude band. We clarify that 40–50°S is not used in this study to define or represent the SAZ. As the reviewer notes, the SAZ position varies across basins. The S(35) isohaline, which marks the STF and the northern SAZ boundary, sits near 40°S in the Atlantic but closer to 43–47°S in the Pacific (Figs. 2, 5b), so a fixed latitude band captures different parts of the frontal system depending on the basin. The 40–50°S band is instead used as a diagnostic window coinciding with the steepest part of the meridional salinity gradient and the largest observed SSS trend in both basins (Fig. 2, upper panels). The SAZ, formally defined as the zone between the STF and SAF, is a moving control volume. As its boundaries shift poleward, part of the salinity change is exported with the migrating fronts rather than accumulating within the zone. The SAZ-mean trend therefore underestimates the actual salinification at these latitudes, while the fixed 40–50°S frame captures the full magnitude of the salinity change where it is most pronounced.

Both views are presented in Fig. 5c: the fixed-latitude frame yields ~0.03 decade^{-1} and the front-following frame ~0.01 decade^{-1} ($p < 0.05$), and the main conclusion is robust across both reference frames.

(2) On the constant SSS proxies. We clarify that S(35) and S(34) are not used as fixed or constant front definitions but as time-varying isohaline positions whose latitudes are diagnosed at each monthly time step. The poleward migration documented in Supplementary Fig. S1 and Fig. 5b is precisely the temporal evolution of these isohaline positions over 2004–2024. The underlying assumption not that SSS is constant at the fronts, but that the salinity values of S(35) and S(34) remain representative of the STF and SAF, respectively, throughout the study period. We confirmed that the salinity contrast across the STF and SAF is maintained throughout 2004–2024. The subtropical and subantarctic water masses retain their characteristic salinity values even as both isohalines migrate poleward, supporting their continued use as frontal proxies. The narrowing of the S(35)–S(34) spacing over this period reflects the asymmetric poleward migration rates of the two fronts (~0.46° decade^{-1} versus ~0.18° decade^{-1}) rather than any drift in the salinity values associated with them. This asymmetric compression is itself a key diagnostic of the frontal corridor narrowing documented in Fig. 5b, and is physically consistent with subtropical high-salinity water expanding poleward faster than the subantarctic water mass retreats. The stability of the proxy salinity values over a two-decade record is expected, given that the large-scale thermohaline contrast between subtropical and subantarctic water masses evolves slowly.

5. Observational data uncertainty: The study relies heavily on Argo profiles, satellite altimetry, and related datasets. While robust, the manuscript does not sufficiently discuss spatial and temporal coverage gaps, measurement uncertainties, potential biases between datasets, and impacts of these uncertainties on diagnosed trends. A dedicated uncertainty discussion would improve credibility.

A dedicated subsection on observational data products and uncertainty has been added to the Methods, addressing each of the four points raised. The revised text reads as follows.

"Data products. Sea surface and subsurface salinity are from the Argo gridded product[60], providing monthly fields on a 1° × 1° grid from the surface to 2,000 dbar. Absolute dynamic topography and surface geostrophic velocities are from the CMEMS Global Ocean Gridded L4 Sea Surface Heights product on a 0.25° daily grid[61]. Dynamic height (0/2,000 dbar) is computed from Argo temperature and salinity fields referenced to absolute surface geostrophic velocities. Wind stress and evaporation are from the OAFlux2 all-satellite analysis[62,63]; precipitation is from GPCP v3.2[64]. Mixed-layer depth is computed from Argo profiles using a density threshold of $\Delta\sigma = 0.03$ kg m^{-3} relative to 10 m. Salinity is reported on the Practical Salinity Scale 1978 (PSS-78) and is dimensionless; all trend quantities carry units of decade^{-1}[65]. All fields are regridded to a common 0.25° monthly grid prior to budget calculation. The study domain covers 120°E–30°E, 65°S–5°S, with a primary analysis period of 2004–2024 and an ECCO validation period of 1992–2017.

Data product uncertainty. Argo float coverage in the Southern Ocean was sparse before 2007 and remains lower in the Indian sector than in the Pacific and Atlantic, consistent with the weaker and patchier salinity signals documented there; the Indian sector is therefore treated separately in Supplementary Note 1. Argo salinity accuracy is approximately 0.01 PSS-78 per profile after delayed-mode quality control, which corrects for conductivity sensor drift, the primary source of systematic bias in Argo salinity records[65]. Altimetric SSH trend uncertainty is approximately 0.3–0.5 cm decade^{-1} after removal of the global-mean sea-level trend[61], small relative to the ADT trends of 2–6 cm decade^{-1} documented in Supplementary Fig. S4. OAFlux wind speed accuracy, evaluated against 126 moored buoys, shows a mean difference of 0.13 m s^{-1} and an RMS difference of 0.71 m s^{-1}, with wind direction mean and RMS differences of 0.55° and 17°, respectively[63]. GPCP v3.2 precipitation uncertainty tends to be elevated at high southern latitudes, where estimates poleward of 58°S rely mainly on infrared-based retrievals[64]. The impact of these uncertainties on diagnosed trends is assessed through a 12-month block bootstrap applied to all trend estimates, with 95% confidence intervals reported throughout; stippling in all spatial trend maps marks grid cells where trends are significant at $p < 0.05$. The poleward migration of $\psi(0)$ and S(35) and the advective dominance in the mixed-layer budget are robust across all bootstrap realizations".

6. Broader impacts of salinity changes: The manuscript focuses primarily on salinity trends but gives limited attention to broader implications of the salinification for marine ecosystems, biodiversity, nutrient cycling, carbon sequestration. Expanding this discussion would provide a

more impactful perspective.

The second-to-last paragraph of the Discussion has been expanded to address the broader implications more explicitly, covering the consequences of poleward subduction pathway shifts for mode and intermediate water ventilation, the effect of salinification-driven density changes on carbon sequestration, and the displacement of the STF as a nutrient boundary with consequences for primary productivity and dependent ecosystems. The revised paragraph reads as follows.

“The broader implications of this reorganization span several oceanic processes. Salinification near 40–50°S increases surface density in a warming Southern Ocean [28], partly offsetting buoyancy gains from surface warming and freshwater input, though the net effect on stratification will vary by season and location. Poleward migration of subtropical gyres further shift the latitudes at which surface waters ventilate the thermocline, rerouting subduction pathways and potentially altering the ventilation of Subantarctic Mode Water and Antarctic Intermediate Water, with downstream consequences for oxygen and nutrient supply to the global thermocline and for carbon sequestration through the solubility pump[55, 56]. Poleward displacement of the STF also shifts the boundary between nutrient-rich subantarctic and nutrient-poor subtropical waters, potential consequences for primary productivity and the ecosystems that track frontal positions. The finding that dynamic redistribution can substantially offset precipitation-evaporation forcing has direct relevance for constraining water cycle amplification from observations, where salinity trends are often used as fingerprints of hydrological cycle change[32, 33, 34].

Minor comments:
L51–54: The roles of the Agulhas Return Current and variability in the Antarctic Circumpolar Current (ACC) are mentioned only briefly. The manuscript would benefit from a clearer explanation of how changes in these currents dynamically influence gyre boundary shifts. In particular, elaborating on the physical mechanisms linking Agulhas Return flow variability and ACC shifts to frontal displacement would strengthen the interpretation.

We have revised these lines to address this explicitly. The revised text reads as follows:

“In the Pacific and Atlantic, poleward-intensifying westerlies displace the zero wind-stress-curl line and the ACC's northern fronts coherently southward, effectively expanding the gyres from both their equatorward and poleward flanks. In the Indian Ocean, the Agulhas Return Current (ARC) introduces additional complexity: its retroflection geometry drives interannual-to-decadal meridional variability in STF position that can partially obscure the underlying secular expansion signal. Variations in ARC retroflection also regulate the magnitude of Agulhas leakage into the South Atlantic, linking Indian Ocean boundary current dynamics to salinity variability across the broader subtropical system.”

L124–126 & L278–279: The authors seem to attribute the deepening of S(34) and S(35) along their poleward flanks to dynamical effect. However, this deepening could result from the poleward shift of curved isohalines, producing a geometric effect rather than true vertical signal

propagation. The discussion appears to mix Eulerian and Lagrangian perspectives, which may contribute to conceptual ambiguity.

We thank the reviewer for this observation. The reviewer raises a valid point: poleward migration of curved isohalines produces an apparent deepening at fixed latitudes in an Eulerian section, a geometric effect that requires no water-mass modification. We have revised both passages to acknowledge this distinction explicitly. The key evidence that the subsurface signal is not purely geometric is the positive salinity trend persisting at fixed depth levels throughout the 40–50°S band (Fig. 2, lower panels). A purely geometric isohaline shift would produce apparent deepening of isohaline contours without a corresponding positive salinity trend at fixed depths. The presence of a coherent positive subsurface trend confirms genuine water-mass change rather than geometry alone. The revised sentences at both locations are as follows.

Results (Surface salinity trends and associated vertical structure): "That the positive salinity trend persists at fixed depth levels throughout the 40–50°S band confirms that this subsurface signal reflects genuine water-mass change rather than the geometric effect of poleward-migrating isohalines captured in an Eulerian frame."

Discussion: "The signal is not surface-limited. Positive salinity trends penetrate to ~500 m at 40–50°S, consistent with a coherent subsurface redistribution of the subtropical high-salinity layer rather than an apparent deepening arising solely from the poleward migration of curved isohalines in an Eulerian frame."

L155-156: Strengthening of gyre does not necessarily induce gyre expansion and shift of the gyre boundary.

We agree. Boundary migration depends on the spatial structure of the transport change, not its magnitude alone. The revised text now reads:

"This positive-inside/negative-outside dipole indicates that transport increases are concentrated on the equatorward flank of $\psi(0)$ with compensating decreases on the poleward flank, a spatial structure that displaces the zero streamline poleward regardless of whether the gyre intensifies overall."

L241-249: The authors argue that the salinity gradient sharpens because S(35) moves poleward faster than S(34). However, the difference in displacement appears relatively small. A quantitative estimate of how much this differential shift contributes to overall salinification would strengthen the argument.

Quantitative estimates have been added. The revised text now reads:

"Figure 5b documents the differential migration underlying this amplification. S(35) migrates ~0.46° decade^{-1} poleward, whereas S(34) migrates only ~0.18° decade^{-1}—a disparity of ~0.28° decade^{-1} corresponding to a frontal corridor narrowing of ~31 km decade^{-1} at 45°S. This compression steepens $|\partial S/\partial y|$, amplifying the cross-front advective salt flux without requiring any change in flow velocity: a steeper gradient increases the salt transported per unit cross-front flow. The quantitative imprint of this steepening is clear in Fig. 5c: the fixed 40–50°S band

records a salinity trend of ~0.03 decade^{-1}, approximately three times the SAZ-mean trend of ~0.01 decade^{-1}, with the ~0.02 decade^{-1} difference reflecting the amplified advective flux as the gradient sharpens."

L304: remove "25"

Corrected. The "25" was a reference number that was inadvertently left without superscript formatting. It now appears as a properly formatted superscript citation.

L305: what do you mean by "so the net stratification tendency is regional and seasonal"?

The sentence has been revised to clarify the physical meaning:

"In a warming Southern Ocean, salinification near 40–50°S increases surface density, partly offsetting buoyancy gains from surface warming and freshwater input; the net effect on stratification varies by season and location depending on the relative magnitudes of the thermal and haline density contributions."

Response to Reviewer 2

The manuscript presents a compelling analysis of Southern Ocean surface salinity trends during 2004-2024 period, attributing salinification to poleward subtropical gyre expansion, advecting high salinity waters southward, dominating over thermodynamic forcing from the intensifying water cycle. The Helmholtz transport streamfunction is used to objectively track gyre boundaries (ψ=0 contour), revealing poleward shifts at −0.49 +/- 0.15°per decade, closely matching S(35) isohaline (tracking the STF) migration of −0.46 +/- 0.04° per decade. This agreement between independent circulation and salinity metrics provides strong evidence that subtropical gyre expansion drives the observed salinification. Finally, mixed-layer salinity budget analysis attributes the 40–50° salinification peak primarily to horizontal advection, while surface freshwater forcing is secondary at these latitudes. The results thus demonstrate that circulation reorganization can dominate regional salinity responses even where net precipitation is increasing.

This is a clear, well written work, providing a strong, multi dataset observationally based analysis with a clear, original, well documented central result, of significance to the field and related fields.

We thank Reviewer 2 for the thoughtful, constructive, and generous assessment of our manuscript. We are particularly grateful for the reviewer's recognition of the central finding and of the multi-dataset observational framework that supports it. The reviewer's comments have been invaluable in strengthening the manuscript, especially our treatment of the mixed-layer salinity budget analysis and the Indian Ocean sector, and we have addressed each point through additional analyses and clarifications. Our detailed point-by-point responses are provided below. Reviewer comments are reproduced in italicized blue text, and our responses are presented in plain text.

Maybe the weakest part of analysis is the mixed-layer salinity budget analysis with a residual of 34%, which potentially may bias advection attribution. It may be useful to test budget analysis results using an ocean state estimate, such as ECCOv4. Reproducing the regional salinity tendency (Fig. 4) and showing that it can be explained primarily by a sum of certain terms in a rigorously closed ECCO budget, would demonstrate that the mechanisms proposed in the manuscript are dynamically consistent and not an artifact due to a large residual term. It would be useful to show a map of the residual in the supplement, to regionally compare the typical magnitude of the residual relative to the observed salinity trend.

We thank the reviewer for this thoughtful and constructive suggestion. We agree that the 34% residual warrants closer examination, and we have addressed the reviewer's concern through new analyses presented in Supplementary Note 2, Supplementary Table 1, and Supplementary Figures 8–10. The reviewer's comment raises three specific concerns: (i) testing the budget against an ocean state estimate such as ECCO v4; (ii) reproducing the regional salinity tendency within a near-closed ECCO budget to confirm that the proposed mechanism is dynamically consistent; and (iii) mapping the residual to enable a regional comparison with the observed salinity trend. We address each in turn below.

(i) ECCO v4r4 budget reconstruction (Supplementary Note 2; Supplementary Table 1). We implemented an independent mixed-layer salinity budget from the ECCO v4r4 state estimate, following the framework of Piecuch (2017) and the ECCO v4r4 budget documentation (ECCO Consortium et al., 2021), including the nonlinear free-surface volume adjustment. The budget was reconstructed on the native LLC90 grid, regridded to a common 0.5° product, and integrated as cumulative anomaly contributions over two periods within the 26-year ECCO record: 1992–2017 (full reanalysis period) and 2004–2017 (overlapping with the Argo record analyzed in the main text). Averaged over the South Pacific and South Atlantic (120°E–30°E, 65°S–5°S), the ECCO budget achieves near-complete closure, with a residual of approximately 0.003 PSS-78 yr^{-1}, less than 1% of the total magnitude of the physical tendency terms in both periods (Supplementary Table 1). The basin-mean salinity tendency emerges as the small net of three large opposing contributions: a salinifying freshwater forcing (~+0.04 PSS-78 yr^{-1}), a freshening net advection (~−0.24 PSS-78 yr^{-1}), and a salinifying net diffusion (~+0.19 PSS-78 yr^{-1}), providing a closed reference budget against which to test the mechanisms diagnosed from observations.

We note one technical aspect of the ECCO budget. Under the nonlinear free-surface formulation, the salinity tendency includes a contribution from volume-flux convergence multiplied by salinity, which is distributed across the individual ADV_h and ADV_v diagnostics and largely cancels in their sum (Supplementary Table 1). We therefore report and analyze the sum (ADV_h + ADV_v) throughout, consistent with standard practice for free-surface state estimates.

(ii) Reproduction of the regional salinity tendency (Supplementary Fig. 8 and Fig.9). In Supplementary Fig. 8, we compare the Argo sea surface salinity trend over 2004–2024 (panel a) with the ECCO mixed-layer salinity trend over 2004–2017 (panel b) and 1992–2017 (panel c). We find that ECCO reproduces the broad structure of the observed Argo salinity trend, with the closest agreement along the Subtropical Front (STF) in the South Pacific. The Pacific salinification signal is present in both ECCO periods and is more spatially coherent over the longer 1992–2017 record, which reduces the imprint of interannual variability and aligns the trend pattern with the climatological STF position. Over the shorter 2004–2017 window that overlaps with Argo, the modeled signal is patchier but still locates the salinification along the same frontal band. In the South Atlantic, the basin-scale salinification seen in Argo is less well captured in either ECCO period. This Pacific–Atlantic contrast is consistent with the expected skill of ECCO v4r4 in basins dominated by mesoscale eddy activity: the LLC90 grid (~1° at the equator, ~40 km at high latitudes) parameterizes rather than resolves mesoscale eddy fluxes (Forget et al., 2015), a known limitation in regions of energetic boundary currents (Menemenlis et al., 2008) such as the Brazil–Malvinas Confluence and Agulhas Return Current (Mason et al., 2017). ECCO therefore supports the advective attribution in the Pacific, where the modeled salinification is captured, while the Atlantic comparison is limited by the spatial resolution of both ECCO and the Argo gridded product in this eddy-rich basin.

In Supplementary Fig. 9, we compare the trend in horizontal advection from the observation-based budget ($-u\cdot\nabla S$, represented by the Ekman and geostrophic components $u_{EK} + u_g$; Methods Eq. 7) with the trend in ECCO total advection (ADV_h + ADV_v). Although the two budgets are constructed differently, we find that both identify the same coherent salinifying advective trend along the Pacific STF, collocated with the climatological STF position and with the observed salinity trend in Supplementary Fig. 8. This convergence supports the dynamical consistency of the advective mechanism proposed in the main text.

(iii) Mapping the spatial structure of the observation-based residual (Supplementary Fig. 10). As suggested by the reviewer, we mapped the trend in the observation-based budget residual ($C\varepsilon$) to enable a regional comparison with the observed salinity trend. Supplementary Fig. 10 shows that the residual trend is small and spatially patchy across most of the domain, and is particularly modest across the Pacific STF salinification band where the advective attribution is centered. Organized residual trends are confined to the eddy-rich frontal regions of the Subantarctic Front and the western South Atlantic, consistent with the spatial scope of a large-scale, time-mean advective decomposition. The Pacific STF salinification band is not collocated with these organized features, supporting the robustness of the diagnosed advective contribution.

Based on the new analyses summarized above, we have revised the residual-justification paragraph in the Results section. The revised paragraph reads:

"The 34% trend-share residual represents the fraction of the observed decadal SSS trend not reproduced by the budget-reconstructed MLS trend. To assess whether this residual could materially affect the dominant-term attribution, we independently reconstruct the MLS budget from the ECCO v4r4 state estimate, which achieves near-complete closure (residual <1% of the physical tendency terms; Supplementary Note 2 and Supplementary Table 1). ECCO reproduces the observed salinification along the Pacific Subtropical Front (Supplementary Fig. 8), and its trend in total advection ($ADV_h + ADV_v$) identifies the same coherent salinifying signal at the gyre boundary as the observation-based diagnostic (Supplementary Fig. 9), supporting the dynamical consistency of the advective attribution. We further map the spatial distribution of the trend in the observation-based residual itself (Supplementary Fig. 10), which is small and spatially patchy across the Pacific Subtropical Front salinification band, with organized signals largely confined to eddy-rich frontal regions of the Subantarctic Front and the western South Atlantic, where mesoscale eddy salt fluxes contribute to the local tendency but lie outside the spatial scope of a large-scale, time-mean advective decomposition. These analyses indicate that the dominant-term attribution at the gyre boundary is robust, and that the geography of the 34% trend-share residual reflects this expected scope rather than a methodological bias in the reconstructed budget."

Indian sector patchiness, with alternating regions of salinification and freshening and only intermittent maxima near the STF is noted, but not analyzed in detail. More information provided in the supplement would be valuable, maybe just adding one supplementary figure and one paragraph in the text summarizing what is known in the Indian sector.

We thank the reviewer for this helpful suggestion. In response, we have expanded both the main text and the Supplementary Information to describe the Indian sector contrast more explicitly.

Our analyses indicate that the Indian sector differs from the Pacific and Atlantic in several respects. First, the zonal-mean S(35) latitude does not exhibit a statistically significant poleward migration in the Indian sector ($-0.02 \pm 0.05°$ decade^{-1}, $p > 0.05$; Supplementary Fig. 1c), whereas significant southward trends are evident in the Pacific and Atlantic ($-0.35 \pm 0.04°$ decade^{-1} and $-0.41 \pm 0.07°$ decade^{-1}, respectively; both $p < 0.05$). Second, the sea-surface salinity trend map (Fig. 1) shows a patchier pattern in the Indian sector STF–SAF corridor, with

alternating regions of salinification and freshening, rather than the more coherent salinification belt seen in the Pacific and Atlantic. Third, the depth–latitude salinity trend section (Supplementary Fig. 2) does not show a comparably coherent subsurface salinification signal in the 40–50°S band, nor a clear penetration of positive trends to ~500 m, in contrast to the Pacific and Atlantic (Fig. 2), where the subtropical high-salinity layer extends continuously into the SAZ.

We further include a brief discussion of factors that may contribute to this contrast. In particular, the wind-stress-curl trend over the southern Indian Ocean appears weaker and less spatially organized than in the Pacific and Atlantic (Supplementary Fig. 3). This may in part reflect modulation by remote ENSO forcing through the Indonesian Throughflow and associated atmospheric teleconnections linked to the Walker circulation, which may weaken the correspondence between local curl trends and the longer-term westerly trend (Wang et al, 2022; Volkov et al. 2020). In addition, the position of the STF in the Indian sector is strongly influenced by the Agulhas Return Current retroflection geometry, introducing substantial interannual-to-decadal frontal variability that may obscure an underlying secular gyre-expansion signal (Beal et al. 2011; Biastoch et al. 2009).

Following the reviewer's suggestion, we have added a short summary paragraph to the Results section ("Surface salinity trends and associated vertical structure"), and we provide further discussion in Supplementary Note 1 together with three supplementary figures 1-3. The added paragraph reads:

"This basin contrast is consistent with differences in the surface forcing. The wind-stress-curl trend in the southern Indian Ocean is weaker and less spatially coherent than in the Pacific and Atlantic (Supplementary Fig. 3a), and the latitude of the zero wind-stress-curl line shows no significant trend ($-0.06 \pm 0.11°$ decade^{-1}, $p > 0.05$; Supplementary Fig. 3d). In the Pacific and Atlantic, by contrast, the zero-curl line migrates significantly poleward at $-0.54 \pm 0.10°$ and $-0.23 \pm 0.10°$ decade^{-1}, respectively ($p < 0.05$; Supplementary Fig. 3b,c), with a combined Pacific–Atlantic trend of $-0.46 \pm 0.43°$ decade^{-1} (Supplementary Fig. 3e). The weaker Indian Ocean curl trend is consistent with previous studies showing that wind-stress-curl variability in this sector is strongly modulated by remote ENSO forcing through the Indonesian Throughflow and atmospheric teleconnections via the Walker circulation (Wang et al, 2022; Volkov et al. 2020), which can offset or mask the secular westerly trend on the timescales considered here. Frontal position in this sector is also influenced by Agulhas Return Current retroflection variability, which contributes additional interannual-to-decadal variability in the STF location (Beal et al. 2011; Biastoch et al. 2009)."

Response to Reviewer 3

This manuscript investigates the observed (2004–2024) increase in Southern Ocean surface salinity, which contrasts with the thermodynamic expectation of freshening under an intensifying hydrological cycle ("salty-gets-saltier, fresh-gets-fresher"). However, besides intensifying the hydrological cycle, anthropogenic global warming is also causing changes in climatological wind patterns. These changes are associated with poleward-shifting midlatitude westerlies in the Southern Hemisphere, and a positive trend of the Southern Annular Mode (SAM). In terms of the wind-driven oceanic circulation, this translates to an intensification and poleward shift of Southern Hemisphere subtropical gyres. The authors therefore link this counter-intuitive salinification to the southward migration of Southern Hemisphere subtropical gyres and the associated high-salinity zone, concluding that dynamic ocean circulation processes dominate over thermodynamic surface freshwater forcing (i.e., precipitation vs. evaporation) in determining the Southern Ocean's salinity trend pattern.

I find the paper interesting, timely, and relevant, especially in the context of understanding and considering the interplay among the several cascading effects of anthropogenic climate change. The analyses are strong, results are compelling, and findings are well presented through the figures. I support publication, provided that several core issues are addressed, primarily related to clarity, the faithful description of basin-specific results and aspects of mechanistic attribution.

While the paper convincingly demonstrates a hemispheric-scale salinification band co-occurring with poleward-shifting circulation features (e.g., S(35)/S(34) isohalines, $\psi(0)$, the zero wind stress curl line, ADT contours), some descriptions appear to overgeneralize across the Pacific and Atlantic basins or do not fully reflect the patterns shown in the figures.

In particular, South Atlantic salinity trends exhibit distinct spatial and vertical structures that differ in important ways from their Pacific counterparts. Faithfully describing these basin-specific features — even if they are not the primary focus of the manuscript — would strengthen the paper and potentially enhance its impact, without altering the main conclusions.

In that context, I outline below a possible interpretation of the South Atlantic trends, supported by relevant studies, in case the authors wish to place these results in a broader dynamical framework.

The South Atlantic salinity trend pattern north of the STF — where surface salinification occurs within the "D-shaped" horizontal subtropical gyre domain (Fig. 1), and subsurface salinification follows the bowl-shaped vertical gyre structure (Fig. 2b, lower panel), bordered by freshening trends north of it (Fig. 2b, upper panel) — is consistent with a salinity accumulation in the upper South Atlantic, associated with a weakening of the Atlantic meridional overturning circulation (AMOC) (Zhu and Liu, 2020).

Similar to the processes leading to Southern Ocean salinification, as found in the present paper, salinity accumulation in the southwestern South Atlantic has been shown to be dominated by the convergence of horizontal transports (Pontes and Menviel, 2024). In line with this scenario, by considering the South Atlantic circulation combined response to changing wind and buoyancy forcings, the AMOC weakening is projected to be compensated by a stronger southwestern South

Atlantic subtropical gyre (SASG) circulation, alongside increasing inflows from the Indian and Pacific Oceans (Marcello et al., 2023).

These interpretations align with the observed South Atlantic salinification pattern shown in Fig. 1, which is consistent with increased entrainment of salty Agulhas leakage waters into the South Atlantic. Because as the AMOC weakens and the SASG strengthens, this favors increased southward (while decreased northward) advection of salty waters from the Indian Ocean Agulhas leakage at the northern boundary of the SASG.

Comparable large-scale trend patterns have been reported in Marcello et al. (2023), who use an ensemble mean to investigate the Atlantic circulation response to external forcing (e.g., compare their Fig. 1 and Fig. S7 with Figs. 3a and S2b here). Their Fig. S6 further illustrates wind pattern changes consistent with the spin-up and poleward expansion of Southern Hemisphere subtropical gyres.

Importantly, such regional South Atlantic circulation dynamics may contribute to reinforcing the Southern Ocean salinification trend pattern identified in the present study, which has thus far been primarily linked to hemispheric-scale wind changes. These mechanisms further support the authors' broader conclusion that dynamic ocean processes can dominate over thermodynamic surface freshwater forcing in shaping regional salinity patterns. Furthermore, I believe their consideration aligns with the author's view of recognizing the interplay among the several cascading effects of modern anthropogenic global warming, as I mentioned.

Below, I provide specific comments that illustrate the general comments highlighted previously, mostly concerning clarity and the faithful alignment between text and figures. Should any of my concerns arise from misinterpretation, I would welcome clarification from the authors and look forward to their responses.

References mentioned:

Zhu, C., & Liu, Z. (2020). Weakening Atlantic overturning circulation causes South Atlantic salinity pile-up. Nature Climate Change, 10(11), 998-1003. https://www.nature.com/articles/s41558-020-0897-7

Pontes, G. M., & Menviel, L. (2024). Weakening of the Atlantic Meridional Overturning Circulation driven by subarctic freshening since the mid-twentieth century. Nature Geoscience, 17(12), 1291-1298. https://www.nature.com/articles/s41561-024-01568-1

Marcello, F., Tonelli, M., Ferrero, B., & Wainer, I. (2023). Projected Atlantic overturning slow-down is to be compensated by a strengthened South Atlantic subtropical gyre. Communications Earth & Environment, 4(1), 92. https://www.nature.com/articles/s43247-023-00750-4

We thank Reviewer 3 for the exceptionally thorough and constructive review of the manuscript. The detailed, line-by-line engagement with both the text and figures, together with the suggestion of a broader dynamical framework for interpreting the South Atlantic results, has materially

strengthened the paper. The reviewer's central observation that several passages overgeneralized across basins and described Pacific-dominant patterns as if they applied equally to the South Atlantic, was well taken and we have revised all identified passages to provide basin-specific descriptions that more faithfully reflect the figures.

We have also incorporated the dynamical framework the reviewer proposed for the South Atlantic, placing the observed salinification in the broader context of AMOC weakening, South Atlantic subtropical gyre intensification, and increased Agulhas leakage. This addition strengthens the mechanistic narrative without altering the main conclusions. All three references suggested by the reviewer (Zhu and Liu, 2020; Pontes and Menviel, 2024; Marcello et al., 2023) have been cited in the revised manuscript at appropriate points. Where a concern arose from a passage we believe was correctly stated, we have revised the wording to eliminate the ambiguity that prompted the comment.

All specific comments are addressed below, point by point. Reviewer comments are reproduced in italic blue and our responses in plain text.

Specific comments:

- Lines 48-51:

"Satellite altimetry shows large positive sea surface height (SSH) trends in the western subtropical basins, particularly the South Pacific, with Argo potential density anomalies extending to at least 2000 m and accounting for more than half of the total trend 14,19,20,21."

The final clause of this sentence is potentially ambiguous. As written, it appears to state that Argo-derived potential density anomalies extending to ~2000 m account for more than half of the observed SSH trend. However, the physical link between deep density anomalies and SSH (e.g., via steric contribution) is implicit and not clearly stated. In addition, the meaning of 'accounting for' and the reference 'total trend' (SSH vs. steric SSH) are unclear. A brief clarification would improve readability for the broad Nature Communications readership.

The sentence has been revised to make the steric pathway explicit, to clarify that the Argo density anomalies contribute to SSH through vertical integration to steric height, and to specify that the reference quantity is the altimetric SSH trend. The physical implication that the SSH rise is largely density-driven rather than mass-driven is now stated explicitly. The revised sentence reads:

"Satellite altimetry reveals large positive SSH trends in the western subtropical basins, particularly the South Pacific, where steric height anomalies derived from Argo density profiles extend to at least 2,000 m and account for more than half of the altimetric SSH trend, indicating that the rise is largely density-driven rather than mass-driven."

- Lines 61-65:

"Early syntheses, constrained by sparse pre-Argo hydrography, appeared consistent with this expectation, showing broad Southern Ocean freshening30,31. Yet recent high-resolution observations reveal a more complex picture, raising a fundamental question: can dynamic

ocean circulation changes overwhelm the thermodynamic signature of an intensifying water cycle?"”

The sentence ‘Yet recent high-resolution observations reveal a more complex picture’ could benefit from clarification. As written, it reads as referring to previously published observations, whereas the following paragraph introduces the authors’ own observational evidence. Clarifying whether this statement refers to prior studies, the present work, or both would improve narrative continuity and avoid a momentary misreading by the reader.

The ambiguity has been resolved. The sentence now clarifies that it refers to both prior studies and the present work:

“Yet recent high-resolution observations, including those presented here, reveal a more complex picture and raise a fundamental question: can dynamic ocean circulation changes overwhelm the thermodynamic signature of an intensifying water cycle?”

- Lines 68-69:

“We show that poleward migration of the subtropical gyres drives this paradoxical salinification…” For clarity, I suggest specifying that this refers to the poleward migration of “southern subtropical gyres”, as the analyses focus on the Southern Hemisphere.

Agreed. “Southern” has been inserted: “We show that poleward migration of the southern subtropical gyres drives this unexpected salinification…”.

- Line 77:

“We also assess how ENSO and the SAM modulate the shift on interannual and decadal timescales. ”

Similarly, I suggest specifying which ‘shift’ is being referred to here (e.g., the gyres’ position shift).

Agreed. The sentence now reads: “We further assess how ENSO and the SAM modulate gyre displacement on interannual to decadal timescales.”

- Lines 78-80:

“Our aim is to provide a falsifiable, mechanism-based account of midlatitude Southern Ocean salinity change that demonstrates how dynamic ocean processes can dominate over atmospheric forcing in determining regional salinity patterns”

I suggest replacing “atmospheric forcing” for “thermodynamic surface freshwater forcing” here, since it is conceptually misleading, because, actually, it is also “atmospheric forcing” (wind-driven circulation changes) that is causing the so-called “dynamic ocean processes”…

The reviewer raises a valid terminological point. “Atmospheric forcing” has been replaced throughout: “…demonstrates how dynamic ocean circulation can dominate thermodynamic surface freshwater forcing in shaping regional salinity patterns.”

- Figure 2 - caption:
I recommend defining the abbreviations used in the panel titles, or writing them in full. 'PAC' and 'ATL' are not defined in the caption, and the meaning of 'SAL' is unclear (e.g., whether it refers to salinity in general or to the linear salinity trend).

The abbreviations have been replaced with full labels in the figure panels. The revised caption reads:

"Figure 2 | Zonal-mean salinity structure and trends in the South Pacific and Atlantic sectors. a Pacific (PAC, 120°E–85°W), b Atlantic (ATL, 85°W–20°E). Upper panels: SSS climatology (grey) and linear trend (red, positive; blue, negative) ad a function of latitude; black and magenta curves show mean SSS for 2004–2008 and 2020–2024, respectively. Lower panels: linear salinity trend from 0–2,000 m (shading) with an expanded view of the upper 0–500 m; stippled where significant at $p < 0.05$. Black and magenta contours denote mean isohalines for 2004–2008 and 2020–2024, respectively. Vertical dashed lines mark the climatological STF (green) and SAF (blue), which coincide at the surface with the S(35) and S(34) isohalines, respectively, defining the sharp SSS gradient across the STF–SAF corridor. All trends are in PSS-78 decade-1. Salinification peaks near 40–50°S and penetrates to ~500 m, consistent with a poleward extension of the subtropical high-salinity layer and a poleward shift of surface isohalines."

- Lines 109-111:
"In the Pacific and Atlantic, the largest positive SSS trends coincide with the sharp SSS gradient between S(35) and S(34) near 40–50°S (the STF–SAF corridor)."

The statement that 'the largest positive SSS trends' coincide with the STF–SAF corridor appears appropriate for the Pacific basin, but seems less accurate for the South Atlantic. In this basin, the largest SSS trends occur predominantly northward of the STF, within South Atlantic subtropical gyre latitudes. It may therefore be more accurate to refer to 'large positive SSS trends', or to differentiate more explicitly between the Pacific and Atlantic basins in this sentence.

The reviewer is correct. The sentence has been revised to describe each basin explicitly:

"In the Pacific, the largest positive SSS trends coincide with the sharp meridional SSS gradient between S(35) and S(34) near 40–50°S, the STF–SAF corridor. In the South Atlantic, comparably large trends extend equatorward of the STF into the subtropical gyre interior, consistent with salinity accumulation associated with a weakening Atlantic Meridional Overturning Circulation (AMOC) and a strengthening South Atlantic subtropical gyre circulation (Zhu and Liu, 2020; Marcello et al., 2023; Pontes and Menviel, 2024)."

- Line 114:
"Equatorward of S(35), the salty subtropics show weaker, patchier increases;"
Again, this statement appears to apply to the South Pacific, but not to the South Atlantic,

where equatorward of S(35) relatively strong SSS trends persist.

The sentence has been revised to describe each basin separately:

"Equatorward of S(35) in the Pacific, the saltier subtropics show weaker, patchier increases; in the South Atlantic, salinification remains comparably strong, persisting across the subtropical gyre interior."

- Lines 114-115:

"Equatorward of S(35), the salty subtropics show weaker, patchier increases; poleward of S(34), trends diminish in the Atlantic but remain locally significant in the Pacific."

Based on the use of expressions such as 'patchier increases' and 'remain locally significant', I assume this sentence refers to the lower panels rather than the upper panels discussed earlier in the paragraph. If so, it would help to explicitly indicate this shift in panel reference.

However, when considering the lower panels, it is not clear that trends poleward of S(34) 'diminish in the Atlantic but remain locally significant in the Pacific'. In particular, the Pacific and Atlantic trend patterns poleward of S(34) appear broadly similar, especially in terms of trend magnitude. In terms of spatial distribution, the patterns are also quite similar, except for the layer between ~500–1000 m, where trends are more heterogeneous in the Atlantic, implying non-statistically significant trends over the broader layer between ~400–1100 m, according to the stippling.

Unless the authors intended this statement to refer to the upper panels, where trends do diminish south of ~55°S in the Atlantic while continuing to increase south of the SAF in the Pacific. However, in that case, the use of 'locally significant' would be confusing, as the upper panels do not display information on trend significance, whereas the lower panels do.

Clarifying which panels this sentence refers to, and revisiting the basin-to-basin contrast described here, would improve consistency between the text and Fig. 2.

The sentence was intended to describe the upper panels. The panel reference is now explicit and "locally significant" removed:

"Poleward of S(34), SSS trends diminish south of approximately 55°S in the Atlantic but remain weakly positive south of the SAF in the Pacific."

- Lines 115-117:

"The climatologically fresh 40–50°S band thus experiences the largest salinification, a pattern fundamentally inconsistent with thermodynamic forcing alone."

*Consistent with points raised above, the statement that the 40–50°S band experiences 'the largest salinification' does not appear fully consistent for the Atlantic basin, where the 30–40°S band shows salinification of comparable magnitude, if not larger. It may therefore be more accurate to state that the 40–50°S band experiences **large** salinification, or to qualify*

this statement more explicitly by basin.

Agreed. The revised sentence reads:
"The climatologically fresh 40–50°S band thus experiences substantial salinification, a pattern fundamentally inconsistent with thermodynamic forcing alone."

- Lines 119-120:
"Depth–latitude trend sections (Fig.2, lower panels) show a vertically coherent salinification maximum centered near 40–50°S,"
Similarly, I suggest rephrasing the end of this sentence to "centered near 35–50°S", which provides a more faithful description of the trend patterns referred to here.

We prefer to retain "centered near 40–50°S." In the manuscript, this phrase denotes the location of the salinification maximum, not the full latitudinal extent of the positive trend. The 40–50°S band is the latitude range used consistently throughout the paper to characterize the poleward boundary of the expanding gyres. This interpretation is supported by Fig. 2: the upper panels show the SSS trend peaking near 40°S in both basins, and the lower panels confirm the vertically coherent subsurface core is anchored within 40–50°S, although the positive anomaly extends equatorward, particularly in the Atlantic.

To acknowledge this equatorward extension, we have revised the sentence:

"Depth–latitude salinity trend sections (Fig. 2, lower panels) reinforce this picture, revealing a vertically coherent salinification maximum centered near 40–50°S. The signal extends to ~500 m and peaks in the upper thermocline (~100–300 m), with positive trends reaching farther equatorward in the Atlantic than in the Pacific."

- Lines 119-127:
"Equatorward of S(35), trends weaken and a shallow freshening develops in the upper 100–200 m, while south of S(34) patchy subsurface salinification persists. Overlaid S(34)–S(37) isohalines show modest poleward and downward displacements of S(34) and S(35) along their poleward flanks, indicating increased subtropical influence across the STF into the STF-SAF corridor. The 40–50°S salinification is therefore not confined to the surface, but represents a redistribution of water masses throughout the upper ocean." I find the description of the lower panels of Fig. 2 in this passage to be neither fully accurate nor internally consistent.

First, I do not believe that the most relevant results highlighted by these panels are being adequately emphasized in the text. Second, I do not think that the Pacific and Atlantic results north of the STF can be described together in a generalized way, as they differ substantially.

In particular, the statement "Equatorward of S(35), trends weaken" appears overly generalized. Trends do not uniformly weaken equatorward of S(35), as they sharply increase north of ~20°S in the Pacific, which still lies equatorward of S(35)/the STF. Moreover, in the Atlantic, trends actually strengthen immediately equatorward of S(35) and only weaken equatorward of ~35°S.

In addition, the statement that "a shallow freshening develops in the upper 100–200 m" is vague and potentially confusing. In the Pacific, only a very minor freshening appears in the upper ~50 m, confined to a relatively narrow meridional band (~21–27°S), whereas a much more pronounced and extensive freshening occurs between ~300–500 m, extending well northward of the STF—an outstanding feature that is not mentioned in the text. In contrast, in the Atlantic, freshening occurs primarily between ~100–250 m north of ~20°S, following a markedly different pattern from the Pacific, while between ~300–500 m north of the STF, modest salinification persists, in contrast to the large freshening feature observed in the Pacific over the same depth layer.

These differences complicate attributing "a shallow freshening in the upper 100–200 m" simultaneously to both basins.

Regarding the passage "Overlaid S(34)–S(37) isohalines show modest poleward and downward displacements of S(34) and S(35) along their poleward flanks", several points require clarification. First, if the discussion focuses only on S(34) and S(35), it may be clearer to refer directly to these isohalines rather than introducing the broader S(34)–S(37) range. Second, S(34) is sparsely present in both panels (except for a short, shallow contour extending southward of the SAF) and instead shows behavior inconsistent with the stated poleward and downward displacements. Third, while S(35) shows an overall poleward and downward displacement in the Atlantic, in the Pacific the poleward displacement is mainly confined to the upper ~150 m, whereas downward displacement occurs primarily north of ~15°S.

A more detailed, basin-specific description would more faithfully reflect the patterns shown in Fig. 2. For example, in the Pacific, the S(34.5) and S(35) isohalines exhibit poleward displacements mainly in the upper ~300 m, while S(35) and S(35.5) show downward displacements north of ~20°S. In the Atlantic, north of the SAF, S(34.5)–S(36.5) display more systematic poleward and downward displacements.

Furthermore, I suggest aligning the text and figure nomenclature. The text refers to S(34)/S(35), while the figure highlights the STF/SAF using blue and green dashed lines. One option would be to relabel the dashed lines as S(34)/S(35), consistent with the terminology used in the text; alternatively, the text could be revised to refer to the STF/SAF instead. At present, it is actually unclear whether references to S(34)/S(35) correspond to the dashed STF/SAF lines or to the black and magenta isohaline contours. Clarifying and standardizing this distinction would help avoid confusion.

We thank the reviewer for these detailed comments. Five distinct concerns were identified and have been addressed.

(i) Trends weaken equatorward of S(35)
The passage now provides basin-specific descriptions: in the Pacific, trends weaken north of ~30°S but strengthen again north of ~20°S; in the Atlantic, trends strengthen immediately equatorward of S(35) and weaken only north of ~35°S.

(ii) Shallow freshening in the upper 100–200 m
Each basin is now described separately. In the Pacific, a pronounced freshening at 300–500 m forms the dominant subsurface feature north of the STF—previously unmentioned and now noted explicitly. In the Atlantic, freshening is confined to 100–250 m north of ~20°S, while modest salinification persists at 300–500 m.

(iii) S(34)–S(37) isohaline range and S(34) displacement
This was a typo; S(35) was intended. The isohaline discussion has been narrowed to S(35), whose displacements are well supported in both basins, with basin-specific differences now described separately.

(iv) Text/figure nomenclature
The Figure 2 caption now states: "Vertical dashed lines mark the climatological STF (green) and SAF (blue), which coincide at the surface with the S(35) and S(34) isohalines, respectively."

(v) Most relevant features not adequately emphasized
The revised passage leads with basin-specific subsurface trend structure and closes by distinguishing genuine water-mass change from the geometric effect of poleward-migrating isohalines in an Eulerian frame.

The revised paragraph reads:
"Depth–latitude salinity trend sections (Fig. 2, lower panels) reinforce this picture, revealing a vertically coherent salinification maximum centered near 40–50°S. The signal extends to ~500 m and peaks in the upper thermocline (~100–300 m), with positive trends reaching farther equatorward in the Atlantic than in the Pacific. Trend magnitudes reach 0.02–0.03 decade^{-1} in the Pacific and 0.03–0.05 decade^{-1} in the Atlantic. The two basins diverge equatorward of S(35). In the Pacific, trends weaken north of 30°S but strengthen again north of 20°S, and a pronounced freshening at 300–500 m forms the dominant subsurface feature north of the STF. In the Atlantic, trends strengthen immediately equatorward of S(35) and weaken only north of ~35°S; freshening is confined to 100–250 m north of 20°S, while modest salinification persists at 300–500 m. South of S(34), patchy subsurface salinification is present in both basins. The overlaid S(35) isohaline is displaced poleward in both basins and downward throughout much of the water column in the Atlantic, consistent with poleward extension of the subtropical high-salinity layer into the STF–SAF corridor. That the positive salinity trend persists at fixed depth levels throughout the 40–50°S band confirms that this subsurface signal reflects genuine water-mass change rather than the geometric effect of poleward-migrating isohalines captured in an Eulerian frame."

***- Line 142:** There appears to be a typographical or sentence break error at 'reorganization In ADT'. I assume 'In ADT' is intended to start a new sentence.*

Confirmed. The missing period has been inserted: "…reorganization. In ADT…"

- Line 145:
The phrase 'poleward-displaced 20–25 dyne cm means in 2020-2024' is unclear. Do the

authors refer to poleward shifts of the 20–25 dyne cm dynamic height contours during 2020-2024 with respect to 2004-2008?

The phrase has been revised: "poleward-displaced 20–25 dyne cm dynamic height contours in 2020–2024 relative to 2004–2008."

- Supplementary Figure S3 - caption:
There is a typo in "25-pointrunning"

Fixed. A space has been inserted: "25-point running"

- Lines 152-153:
"In each, opposite-signed trend anomalies straddle ψ(0)"
I suggest adjusting to "opposite-signed large-scale trend anomalies", as this feature is not immediately apparent from a detailed visual inspection of Fig. 3a.

Incorporated: "In each, opposite-signed large-scale trend anomalies straddle ψ(0), but the sign order differs."

- Lines 192-194:
"The observed pattern shows a salinification belt near 40–50°S, weaker salinification from 20–35°S, and patchy freshening south of the SAF. The budget reconstruction reproduces the overall features (Fig.4a,b)"

I am somewhat confused by this passage. It is unclear whether both sentences refer to Fig. 4a,b, or whether the first sentence refers to the observed pattern shown in Fig. 1, while the second sentence intended to compare Fig. 4a,b against that pattern.

In either case, I find it difficult to clearly identify the features described in Fig. 4a,b. In particular, I do not clearly see a continuous "salinification belt near 40–50°S" that stands out relative to other salinity trend features in these panels. Likewise, the description of "weaker salinification from 20–35°S" is not obvious. For example, over 20–35°S and ~150°E–150°W, salinification appears relatively strong in Fig. 4a. Moreover, based on Fig. 4b, salinification over 20–35°S seems more spatially continuous than over 40–50°S.

I therefore recommend revisiting the description of these figures to ensure that it closely reflects the patterns actually shown in Fig. 4a,b, rather than a more schematic or expected representation of the results.

One possible way to assess whether salinification is indeed stronger over 40–50°S and weaker over 20–35°S would be to derive time series of mean salinity over these latitude bands. This could help clarify the relative behavior between regions and aid the description and interpretation of the results.

Three concerns are addressed.

(i) Panel reference ambiguity.

The first sentence now refers explicitly to the observed SSS trend (Fig. 1) and the second to the budget reconstruction (Fig. 4a).

(ii) Salinification belt identification.
"The salinification belt" has been replaced with "the salinification maximum near 40–50°S" and "weaker salinification from 20–35°S" with "secondary salinification at 20–35°S" to avoid implying a magnitude hierarchy not universally supported across both basins.

(iii) Latitude-band mean time series.
Domain-mean SSS trends averaged over 40–50°S and 20–35°S have been computed. In the Pacific, the two bands show comparable trend magnitudes, consistent with the reviewer's observation; in the Atlantic, the 40–50°S band trend is stronger. These values have been noted in the text.

The revised passage reads:
"The budget-reconstructed MLS trend C_{TOT} (Fig. 4a) reproduces the dominant spatial features of the observed decadal SSS trend (Fig. 1), including the salinification maximum near 40–50°S, secondary salinification at 20–35°S, and patchy negative trends south of the SAF."

- Figures 3a and 4a,b:
The "60ºE" x-axis label should be "60ºW" in these figures. I noticed this error also occurs in Figures S4 and S6.

Corrected in all four figures. We thank the reviewer for catching this error.

- Lines 196-210:
*"Horizontal advection is the leading driver across the STF–SAF corridor, demonstrating that dynamic ocean circulation overwhelms thermodynamic forcing in this region. Ekman advection maximizes in a zonal band near 40–50°S, while geostrophic advection peaks along western boundary current extensions and frontal jets (e.g., downstream of the EAC extension and at the BMC) (Supplementary Fig.S6a-b). As in Fig.2 (upper panels), the meridional SSS gradient | ∂S/∂y| is steepest between S(35) and S(34). Consequently, the meridional Ekman term vEK∂S/∂y dominates **u**EK×ÑS, producing the strongest saline advection across the STF. These advective maxima align with the poleward displacement of ψ(0) and the ψ-trend dipole (Fig.3), linking the budget signal to gyre migration. Entrainment and FWF modulate but are secondary at 40–50°S (Supplementary Fig.S6c). Entrainment salinifies north of the STF and freshens south of the SAF, consistent with deeper mixing entraining saline ventilated-thermocline waters to the north and fresher Antarctic Intermediate Water (AAIW) -influenced waters 45 to the south (Fig.2, lower panels). FWF salinifies the interior subtropics and weakly freshens poleward (Supplementary Fig.S6d), as expected from the precipitation-evaporation flux, but its contribution is minor compared to advection at 40–50°S. Diffusion is small and spatially noisy (not shown)."*

This is a long and very heavy paragraph that could use flow improvement and further clarification. My main comments below revolve around one core issue, that mechanistic attribution seems to be asserted more strongly than it is demonstrated or clearly explained.

- *First, I suggest the authors start this paragraph by initially clarifying they're discussing Fig. S6. Something along the lines "The linear trends of main budget terms are shown in Fig. S6". This would help to first anchor the reader to understand that the upcoming discussion refers to results in this figure. Because when I read the first sentence, I automatically thought the authors were referring to Fig. 4c—considering that they were discussing Fig. 4a,b at the end of the previous paragraph.*

- *I believe the authors are allowed to use the simplified nomenclature "Fig. S6" instead of "Supplementary Fig. S6" — this would help simplify the paragraph a little, since the different panels of Fig. S6 are called individually later on.*

- *The use of "horizontal advection" in the first sentence is confusing, especially for non-experts, as Fig. S6 only shows Ekman and geostrophic advection as separate terms. I suggest clarifying whether 'horizontal advection' refers to the sum of Ekman and geostrophic advection, and to state this explicitly in the text.*

Concerning this passage: "As in Fig.2 (upper panels), the meridional SSS gradient |∂S/∂y| is steepest between S(35) and S(34). Consequently, the meridional Ekman term vEK∂S/∂y dominates ***u**EK*×∇S, *producing the strongest saline advection across the STF. ":*

- *Similarly, it is confusing to suddenly address "the meridional Ekman term" — which is not shown separately anywhere in the paper. This might induce non-expert readers, particularly, to go and look for this term somewhere in the figures/text. Alternatively, I suggest rephrasing it to "the meridional component of the Ekman advection term (not shown separately)".*

- *As written, "As in Fig.2 (upper panels), the meridional SSS gradient", it gives the impression that the authors are comparing Fig. S6 with Fig. 2, which is not the case. I would suggest rephrasing this passage to "Because the meridional SSS gradient |∂S/∂y| is steepest between S(35) and S(34), the meridional … . ";*

- *However, the opposite seems to be the case—i.e., meridional SSS gradients being steepest between S(35)-S(34) potentially as a consequence (at least partially) of the Ekman advection term being dominated by its meridional component…*

- *Still, as the authors do not provide concrete evidence for this claim (i.e., for the link between steepest SSS gradients between S(35)-S(34) and the dominance of the meridional component in*

the Ekman advection term), they should clarify this is their assumed hypothesis. This could be done by simply clarifying that "the (implied or suggested) dominance of the meridional component in the Ekman advection term" aligns with "strong saline advection across the STF" and "steepest SSS gradients between S(35)-S(34) (Fig. 2, upper panels)".

- *About "Entrainment and FWF modulate but are secondary at 40–50°S (Supplementary Fig.S6c)." — it is unclear what does entrainment modulate?! Also, I believe Fig. S6c, d should be referenced here, since FWF is also being addressed.*

- About "Entrainment salinifies north of the STF and freshens south of the SAF, consistent with deeper mixing entraining saline ventilated-thermocline waters to the north and fresher Antarctic Intermediate Water (AAIW) -influenced waters 45 to the south (Fig.2, lower panels).":

- I suggest Fig. S6c is then individually referenced here: "Entrainment salinifies north of the STF and freshens south of the SAF (Fig. S6c)"

*- However, in my opinion, the authors are again overgeneralizing results for the Pacific and Atlantic basins, with a focus on the Pacific—thus neglecting important and differentiated results for the Atlantic basin. More specifically, if one looks at the Atlantic in Fig. S6c, entrainment actually salinifies predominantly *in between* the STF and SAF, and north of the STF to a much lesser degree; while it does not freshen immediately south of, or along, the SAF, as in the Pacific.*

- "FWF salinifies the interior subtropics and weakly freshens poleward (Supplementary Fig.S6d)". Again, when comparing the Pacific and Atlantic basins in Fig. S6d, opposed patterns are observed over subtropical latitudes, with the Pacific showing salinification mainly over the South Pacific Subtropical Gyre center (analogous to "interior subtropics"), and the Atlantic in turn showing freshening over the corresponding region, and salinification along its boundary currents instead. Additionally, there is basically no freshening south of the STF in the Atlantic domain. At least it seems not to be statistically significant. I further suggest the authors include stippling in Fig. S6 to indicate where linear trends are statistically significant. In this case, stippling could be used only over regions where trends are not significant instead, not to overwhelm the figure aesthetics.

The comment raises several concerns about clarity and faithful basin-specific description. Each is addressed below.

(i) Paragraph opening — Supplementary Fig. S7 not anchored.
The revised paragraph now opens with: "Horizontal advection, the sum of Ekman and geostrophic contributions, is the leading driver across the STF–SAF corridor," immediately defining the term and anchoring the discussion to Supplementary Fig. S7.

(ii) "Horizontal advection" undefined.
Now defined explicitly on first use as above.

(iii) "Meridional Ekman term" not shown separately.
The original phrase has been removed. The revised text instead states: "Because the meridional SSS gradient $|\partial S/\partial y|$ is steepest between S(35) and S(34) (Fig. 2, upper panels), the cross-front Ekman component dominates, producing the largest positive trend contribution across the STF."

(iv) "As in Fig. 2 (upper panels)" reads as a figure comparison.
Rephrased causally: "Because the meridional SSS gradient $|\partial S/\partial y|$ is steepest between S(35) and S(34)…"

(v) Causal direction asserted without direct evidence.

The language now frames the statement as physically consistent with observations rather than a directly demonstrated causal claim.

(vi) Entrainment reference and "modulation" unclear.
"Entrainment is an important secondary contributor at 40–50°S, while surface freshwater flux plays a minor role (Supplementary Fig. S7c–d)" replaces the ambiguous original.

(vii) Entrainment overgeneralizes Pacific and Atlantic.
Basin-specific descriptions have been added: "In the Pacific, positive contributions are concentrated along the STF and SAF but are patchy elsewhere; in the Atlantic, C_ENT is predominantly positive over the subtropical interior north of the STF and along the STF (Supplementary Fig. 7c), consistent with deeper mixing entraining saline ventilated-thermocline waters from above and fresher Antarctic Intermediate Water (AAIW)-influenced waters from below (Fig. 2, lower panels)."

(viii) FWF overgeneralizes; stippling requested.
Basin-specific FWF description has been added: "C_{FWF} is positive over the subtropical interior north of the STF and negative poleward of it in both basins (Supplementary Fig. S7d), confirming its minor role at 40–50°S." Stippling indicating statistical significance has been added to Supplementary Fig. S7 as specifically requested.

- Lines 212-213:
"The dominant-term map (Fig.4b) identifies, at each grid point, the process giving the largest contribution to the observed SSS tendency trend."

- "The dominant-term map (Fig.4b)" — the correct reference here is Fig. 4c.

- The phrase 'SSS tendency trend' is somewhat confusing, as 'tendency' already implies a rate of change. It may be clearer to refer simply to the 'observed SSS trend', or to clarify whether this refers to the trend of the SSS tendency itself (which would correspond to a second derivative in time, and does not appear to be the intended meaning here).

Both corrected. The revised sentence reads:
"The dominant-term map (Fig. 4b; Methods) reveals a spatially coherent process hierarchy."

- Lines 213-215:
"It confirms that horizontal advection, primarily Ekman, dominates along the STF, with geostrophic dominance confined to boundary currents and frontal jets. Poleward of the SAF, Ekman remains prevalent, with localized patches where entrainment or FWF lead. Over the interior subtropics (~20–35°S), FWF and entrainment co-lead, while advection contributes in localized patches."
Concerning the first sentence, when considering both the Pacific and Atlantic basins, I would say that horizontal advection (both Ekman and geostrophic) dominates along/around the STF, with Ekman advection predominating only over the Pacific domain. I would also say that geostrophic advection seems to dominate along the SAF, mainly in the Pacific, but also in the Atlantic, to a lesser extent.

- Regarding the second sentence ("Poleward of the SAF, Ekman remains prevalent, with localized patches where entrainment or FWF lead."), I would include geostrophic advection to "entrainment or FWF", as it appears poleward of the SAF maybe even to a higher extent than FWF, for example.

- For the third/last sentence, I would just replace "advection" with "horizontal advection", for consistency with the terminology used elsewhere.

All three points have been addressed:

"Ekman advection controls the STF region in the Pacific; in the Atlantic, both Ekman and geostrophic advection govern the STF and its immediate flanks. Geostrophic advection leads along the SAF in both basins, more prominently in the Pacific. Poleward of the SAF, Ekman advection remains prevalent, with localized patches of geostrophic advection, entrainment, or freshwater-flux dominance. Over the interior subtropics (~20–35°S), freshwater flux and entrainment co-dominate."

- Lines 223-226:

"Importantly, horizontal advection (Ekman plus geostrophic) accounts for more than one-third of the domain-mean trend, establishing dynamic ocean circulation as the primary driver of Southern Ocean salinification and relegating surface freshwater forcing to a secondary role 7, 46."

- I would add here that, besides 'horizontal advection (Ekman plus geostrophic) accounting for more than one-third of the domain-mean trend' (Fig. 4d), it clearly dominates south of ~35ºS in both the Pacific and Atlantic basins (as according to Fig. 4c)—this regional dominance being therefore the main feature "establishing dynamic ocean circulation as the primary driver of Southern Ocean salinification…", rather than the full domain-mean contribution alone.

The reviewer makes a valid and strengthening point. The domain-mean trend share is now stated alongside the spatial pattern: "Integrated over the study domain (Fig. 4c; Methods), horizontal advection (Ekman plus geostrophic) accounts for the largest share (C_{EK} 23%, C_g 13%; total 36%), followed by entrainment (C_{ENT} 17%), freshwater flux (C_{FWF} 11%), and horizontal diffusion (2%), with a residual ($C\varepsilon$) trend share of 34%. The detailed examination of the residual using an independent ECCO v4r4 budget reconstruction (Supplementary Note 2 and Supplementary Figs. 8–S10) confirms that the dominant-term attribution at the gyre boundary is robust to the residual term."

- Lines 228-230:

"Equally important is the spatial structure: maxima in vEK∂S/∂y co-locate with the climatologically steep S(35)–S(34) gradient (Fig.2), implying that frontal migration projects most strongly onto the fixed 40–50°S band."

- As raised before, the claim that "maxima in vEK∂S/∂y co-locate with the climatologically steep S(35)–S(34) gradient" does not appear to be sufficiently supported, as I assume that

“vEK∂S/∂y” is not shown individually, anywhere in the manuscript. I suggest clarifying how this co-location is demonstrated, or alternatively moderating the wording of this statement.

The sentence now refers directly to the displayed quantity: “These advective maxima align with the poleward displacement of $\psi(0)$ and the ψ-trend dipole (Fig. 3), directly linking the budget signal to gyre migration.”

- Discussion:

- I suppose the authors should mention again in the Discussion that their Southern Ocean results are focused on the Pacific and Atlantic basins.

Agreed. The opening Discussion paragraph now states: “Dynamic ocean circulation changes substantially offset thermodynamic forcing from the intensifying water cycle, driving a wind-driven reorganization of subtropical gyre pathways that increases surface salinity poleward of ~35°S.”
A clarifying sentence directs readers to the Indian sector analysis: “The Indian sector departs from this picture, exhibiting distinct dynamical behavior arising from weaker and spatially less organized wind-stress-curl trends and frontal control by Agulhas Return Current retroflection geometry, and is therefore treated separately (Supplementary Note 1).”

Line 288:

“The 40–50°S salinification peak arises from the advective pathway.”
- I suggest adjusting to: “...from horizontal advective pathways”

Agreed. The revised sentence reads: “The 40–50°S salinification peak is therefore fundamentally a dynamical phenomenon, its spatial pattern and magnitude set by horizontal advection rather than by any intensification of the local water cycle.”

- Lines 303-304:

“In a warming Southern Ocean 25” — the link to reference “25” is missing here.

Corrected. The superscript citation now appears with proper formatting.

- Reference suggestion:

The authors may find it useful to consider the paper of Ge et al. (2023), which states that “wind-driven alterations in the geostrophic circulation cause SSS advection across zonal salinity gradients. These processes drive SSS changes that are opposite in sign to local FWF changes and complicate the regional SSS-FWF relationship in the long-term change since 1970.” (https://agupubs.onlinelibrary.wiley.com/doi/pdf/10.1029/2023JC020207).

We thank the reviewer for this suggestion. Ge et al. (2023) provides valuable independent support for the central argument of this paper and has been cited in two places: in the Discussion opening paragraph, where it supports the finding that wind-driven geostrophic circulation can drive SSS changes opposite in sign to local freshwater-flux anomalies; and in the Results, where horizontal advection is identified as the primary driver.

Response to Reviewer #1

We thank the reviewer for the careful follow-up and for recognizing the substantial revisions made in the previous round. The remaining comments identify three places where the manuscript needed a clearer distinction between fixed-Eulerian trends, front-following changes, and changes on density surfaces. We have revised the relevant text and added quantitative diagnostics to make these distinctions explicit. All three points are addressed in turn below. The reviewer's comments are shown in blue italic and our responses in black.

The authors have addressed the majority of my previous comments, and I sincerely appreciate their thorough efforts in revising the manuscript. However, there are still a few key points and physical interpretations that require further clarification before the manuscript is finalized.

Comments:

1. In their response and in the text (L150-153), the authors state: "A purely geometric isohaline shift would produce apparent deepening of isohaline contours without a corresponding positive salinity trend at fixed depths." (in response). I have to disagree, a purely geometric shift (like a poleward displacement of curved isohalines) can and will produce a salinity trend at a fixed Eulerian depth. As the sloping structure shifts laterally past a fixed geographic point, a deeper (or shallower) part of the water column is sampled, generating an apparent Eulerian trend without any true water-mass modification.

The reviewer raises an important point, and we have revised the relevant text accordingly. We agree that in a fixed Eulerian frame, vertical or lateral displacement of sloping isohalines can produce a salinity trend at fixed latitude and depth with no change in water-mass properties, and that fixed-depth trends alone therefore cannot distinguish this geometric (heave) effect from salinity change on density surfaces. We have revised the previous categorical wording by using a quantitative decomposition to separate the two contributions.

We decompose the fixed-depth salinity trend as, $(\partial S/\partial t)|_z \approx (\partial S/\partial t)|_\sigma - (\partial S/\partial z) \times (\partial z_\sigma/\partial t)$, where $(\partial S/\partial t)|_z$ is the Eulerian salinity trend at fixed depth, $(\partial S/\partial t)|_\sigma$ is the salinity trend on density surfaces (water-mass change), and $-(\partial S/\partial z) \times (\partial z_\sigma/\partial t)$ is the heave contribution from the local change in depth of density surfaces, which can arise from local vertical motion or from lateral displacement of sloping isopycnals past a fixed point.. The results are shown in new Supplementary Figs. 5 and 6.

In the upper 500 m of the 40–50°S band, both terms are positive in every sector (Supplementary Fig. 6a). The combined (PAC+ATL) trend (~0.014 decade^{-1}) comprises ~60% heave and ~40% salinity change on density surfaces; the balance is heave-dominated in the Pacific and density-surface-dominated in the Atlantic (~75%). The water-mass component is thus positive in all sectors, confirming that the subsurface trend is not a geometric artefact.

We emphasize that both contributions are dynamical consequences of the wind-driven gyre reorganization rather than thermodynamic responses to local freshwater forcing. The heave term reflects deepening of subtropical isopycnals at fixed depths, driven by intensified Ekman

pumping and by the poleward displacement of sloping isopycnals as the gyre expands — a direct kinematic signature of the same circulation change documented throughout the manuscript. The density-surface term reflects poleward advection of saltier subtropical water along isopycnals. The subsurface salinification therefore remains a coherent dynamical redistribution of the subtropical high-salinity layer, consistent with the mechanism we propose.

We note that the decomposition is best constrained in the upper 500 m, where the signal resides; the 0–2,000 m integral (Supplementary Fig. 6b) is dominated by near-zero, weakly stratified deep layers in which the two terms are individually large and partly cancel, and is shown only for completeness.

The Results and Discussion have been revised accordingly. The following paragraphs are added to the current revised manuscript.

Results, lines 158-182:

"Because the analysis is performed in an Eulerian frame, fixed-depth salinity trends combine salinity changes on density surfaces with the heave effect of vertically displaced isopycnals. We decompose the fixed-depth salinity trend as,

$$(\partial S/\partial t)|_z \approx (\partial S/\partial t)|_\sigma - (\partial S/\partial z) \times (\partial z_\sigma/\partial t)$$

where $(\partial S/\partial t)|_z$ is the Eulerian salinity trend at fixed depth, $(\partial S/\partial t)|_\sigma$ is the salinity trend on density surfaces (water-mass change), and $-(\partial S/\partial z) \times (\partial z_\sigma/\partial t)$ is the heave contribution from vertical displacement of density surfaces (Supplementary Figs. 5, 6). In the upper 500 m of the 40–50°S band, both components are positive in each basin, and the Pacific–Atlantic mean (~0.014 $decade^{-1}$) comprises ~60% heave and ~40% salinity change on density surfaces, with heave the larger contributor in the Pacific and water-mass change the larger contributor in the Atlantic (~75%). Both terms are dynamical. The heave component reflects a deepening of subtropical isopycnals at fixed locations, which can arise either from local downwelling driven by the poleward-shifting, downwelling-favorable wind-stress curl, or from the lateral displacement of sloping isopycnals as the subtropical gyre expands poleward. In both cases, a deeper and saltier portion of the water column is drawn to fixed depths, and both pathways are kinematic signatures of the wind-driven circulation change documented above. The density-surface component reflects poleward advection of saltier subtropical water masses along isopycnals, most pronounced in the Atlantic. This component is concentrated in the surface and shallow thermocline because the subtropical salinity maximum is itself an upper-ocean feature: poleward displacement of subtropical waters produces the largest salinity changes on shallow density surfaces, where the climatological contrast between subtropical and subantarctic waters is greatest, and the signal weakens with depth as the contrast diminishes. Neither component is a thermodynamic response to local freshwater flux. The subsurface salinification is therefore a coherent dynamical redistribution of the subtropical high-salinity layer rather than an Eulerian artefact of poleward-migrating isohalines."

Discussion an implications, lines 388-394:

"The signal is not surface-limited. Positive salinity trends penetrate to ~500 m at 40–50°S, where both wind-driven deepening of subtropical isopycnals (heave) and salinification of water masses on density surfaces contribute positively in each basin, with heave the larger contributor in the Pacific and water-mass change the larger contributor in the Atlantic. Both are dynamical

expressions of the gyre reorganization rather than a thermodynamic response to local freshwater forcing, so the subsurface salinification represents a coherent redistribution of the subtropical high-salinity layer."

2. The authors state that over 2004–2024, the meridional salinity gradient ∂S/∂y steepens in the STF–SAF corridor due to faster poleward migration of S(35) relative to S(34), and argue that this steepening progressively reduces this freshening tendency, leading to a positive trend contribution (salinification) from the -vEK ∂S/∂y term. There appears to be a fundamental algebraic and physical contradiction in this logic that needs to be resolved: if the front sharpens and the gradient steepens as described, the magnitude of ∂S/∂y increases. Mathematically, multiplying a larger positive gradient by a positive vEK and a negative sign must result in a larger negative value, which means enhanced freshening, not salinification.

We appreciate the reviewer raising this important point. We have revised the interpretation and added a new fixed-grid decomposition of the Ekman salinity tendency to diagnose the sign of this contribution directly (Supplementary Fig. 11).

We first clarify the quantities involved. The total instantaneous Ekman salinity tendency used in the mixed-layer budget is:

$$T_{EK} = -u_{EK}\,\partial S/\partial x - v_{EK}\,\partial S/\partial y,$$

where u_{EK} and v_{EK} are the zonal and meridional Ekman velocities, and $\partial S/\partial x$ and $\partial S/\partial y$ are the zonal and meridional salinity gradients. The corresponding budget contribution, C_{EK}, is obtained by cumulatively integrating T_{EK} and fitting a linear trend to the resulting time series (Methods, Eqs. 8-9). This full Ekman contribution, including both zonal and meridional components, is the quantity reported as 23% of the domain-mean trend share in Fig. 4c.

The reviewer's comment concerns the meridional component of this tendency:

$$T_{EK,y} = -\,v_{EK}\,\partial S/\partial y$$

With y defined positive northward. Across the STF–SAF corridor, salinity generally increases northward, so $\partial S/\partial y > 0$. Therefore, if v_{EK} remains northward, an increase in $\partial S/\partial y$ makes $-\,v_{EK}\,\partial S/\partial y$ more negative, corresponding to enhanced freshening rather than salinification. We have revised the text to make this sign relationship explicit.

To diagnose this quantitatively, we decomposed the 2004–2024 fixed-grid trend of the meridional Ekman tendency as:

$$\text{trend}(T_{EK,y}) = -\,[\text{trend}(v_{EK}) \times \overline{\partial S/\partial y}] - [\overline{v_{EK}} \times \text{trend}(\partial S/\partial y)] + R_{cov}$$

where overbars denote 2004–2024 means. The first term (velocity) is the contribution from changes in meridional Ekman transport. The second term (gradient) is the contribution from changes in the local fixed-grid meridional salinity gradient. The third term, $R_{cov} = \text{trend}\,[-v_{EK}'\,(\partial S/\partial y)'\,]$ is the covariance residual, with primes denoting departures from the monthly mean. This decomposition closes to numerical precision in both basins (Supplementary Fig. 11).

The fixed-grid trend in ∂S/∂y is positive in both the Pacific and Atlantic sectors of 40–50°S (Supplementary Fig. 11c), and the corresponding gradient term is negative in both basins. The sign of the meridional Ekman tendency trend is therefore determined by the balance among the velocity, gradient, and covariance contributions, not by the gradient term alone.

In the Pacific sector, the meridional Ekman tendency trend is positive, driven primarily by the velocity term: northward Ekman transport weakens over 2004–2024 as the westerly wind maximum shifts poleward, reducing the climatological freshening flux at fixed grid points. This velocity contribution outweighs the opposing gradient term. In the Atlantic sector, the meridional Ekman tendency trend is negative, indicating that the meridional Ekman component contributes to freshening there; the salinification in the Atlantic 40–50°S band is driven primarily by geostrophic advection and entrainment (Fig. 4b; Supplementary Fig. 10b,c).

We have accordingly revised the manuscript to interpret the Ekman contribution through the explicit velocity–gradient decomposition. The front-following description of frontal sharpening is retained only as part of the broader gyre-expansion interpretation, where it explains why fixed latitudes near 40–50°S are especially sensitive to poleward frontal displacement. As the sharped salinity front migrates poleward through this band, saltier subtropical water progressively replaces fresher subantarctic water at those fixed locations. This frontal-replacement mechanism operates through the total advective field and is distinct from the sign of the meridional Ekman tendency trend. The revised interpretation is consistent with the central conclusion that the 40–50°S salinification is dynamically linked to poleward gyre expansion and horizontal redistribution of subtropical high-salinity waters; horizontal advection remains the leading budget contribution, while surface freshwater forcing opposes the salinification over the poleward part of the band.

Related changes in the manuscript can be found in the following five places:

- lines 25-26 (Abstract): “this differential migration narrows the frontal corridor and sharpens the cross-frontal gradient, concentrating the poleward salt redistribution”.
- lines 262-270 (Results, MLS budget): “A decomposition of the 2004–2024 meridional Ekman tendency trend into velocity, gradient, and covariance contributions (Supplementary Fig. 11) shows that at 40–50°S the sign of the trend reflects the balance among these terms. In the Pacific, weakening northward Ekman transport associated with the poleward shift of the westerly wind maximum is the primary positive contribution, outweighing the opposing gradient term. In the Atlantic, both terms contribute to freshening; salinification there is driven primarily by geostrophic advection and entrainment (Supplementary Fig. 10b,c). The full Ekman contribution CEK, including both zonal and meridional components, remains positive at 40–50°S (Supplementary Fig. 10a).”
- lines 343-348 (Results, STF-centered salinification): “This compression sharpens the cross-frontal |∂S/∂y|, so that as the front migrates poleward through the 40–50°S band, fresher subantarctic surface water is progressively replaced by saltier subtropical water. The fixed 40–50°S band records a salinity trend of ~0.03 decade^{-1} (Fig.5c), approximately three times the SAZ-mean trend of ~0.01 decade^{-1}, with the difference

reflecting the concentrated salinity change within the narrow band traversed by the migrating front."

- lines 369-374 (Results, STF-centered salinification): "Poleward gyre expansion drives a faster poleward shift of S(35) than S(34), narrowing the S(35)–S(34) corridor and sharpening the cross-frontal salinity gradient. As this sharpened front traverses the 40–50°S band, fresher subantarctic water is progressively replaced by saltier subtropical water, producing the observed SSS trend peak."
- lines 416-420 (Discussion): "Second, S(35) advances faster than S(34), narrowing the frontal corridor and sharpening the cross-frontal gradient; as this sharpened front migrates poleward through 40–50°S, saltier subtropical water progressively replaces fresher subantarctic water, concentrating the advective salinity increase within the narrow band traversed by the front."

We have also revised the Fig. 5 caption, where the final sentence now reads: "The nearly threefold difference between frames reflects the concentrated salinity change at fixed latitudes as the sharpened front migrates poleward through the 40–50°S band."

3. I find the authors' justification regarding the frontal definitions contradictory. The text states that S(34) and S(35) are not used as constant frontal proxies, but then asserts that these exact values are assumed to be representative of the SAF and STF across the entire 20-year period. This is a contradiction in terms. If you define a moving front by a fixed salinity value, you are using a constant salinity proxy. Please revise the text to clearly acknowledge that you are tracking specific isohalines as proxies for the fronts, and clarify how you have verified that a broad-scale salinity trend hasn't artificially artificially shifted these isohalines away from the true dynamical fronts (i.e., the maximum gradients).

We thank the reviewer for this comment. We have revised the manuscript to make the frontal terminology more precise. The S(35) and S(34) contours are fixed salinity values whose latitudinal positions vary in time; we now refer to them explicitly as fixed-salinity isohaline proxies for the surface expressions of the STF and SAF, respectively.

Regarding whether a broad-scale salinity trend may have displaced these isohalines away from the frontal-gradient structure, three lines of evidence already presented in the manuscript support the use of these isohalines as frontal proxies over the study period.

First, the S(35) and S(34) isohalines migrate at markedly different rates (−0.46° and −0.18° decade^{-1}; Fig. 5b). A basin-wide salinity offset alone would not readily produce this 2.5-fold disparity, which instead points to differential frontal displacement driven by asymmetric gyre expansion.

Second, the S(35) migration rate agrees closely with the poleward shift of the gyre-edge streamfunction ψ(0), a diagnostic derived from the velocity field and independent of the salinity values used to define the isohalines. This agreement links the isohaline displacement to a circulation change rather than to a salinity offset alone.

Third, the SSS trends themselves are spatially concentrated and basin-dependent. In the Pacific, the 40–50°S band shows a significant positive trend (0.024 decade^{-1}, $p < 0.05$) while the 20–

35°S band does not (0.009 decade^{-1}, $p > 0.05$; Supplementary Fig. 4a), consistent with a trend centered on the frontal band rather than a basin-wide offset. In the Atlantic, both the 40–50°S and 20–35°S bands salinify, but the 40–50°S trend is stronger (0.047 versus 0.038 decade^{-1}; Supplementary Fig. 4b), further indicating that the trend structure is basin-dependent rather than spatially uniform.

Taken together, the differential migration, the agreement with a salinity-independent circulation metric, and the latitude-concentrated SSS trends support the interpretation that the tracked isohalines reflect coherent frontal-zone displacement over the study period.

We have added a new Methods subsection, "Frontal isohaline proxies" (lines 531-541), to make the proxy terminology and its supporting evidence explicit. The word "proxy" has also been applied consistently wherever S(35) and S(34) are introduced in the Results.

The new subsection reads:

"The latitudinal positions of the S(35) and S(34) isohalines are tracked as fixed-salinity proxies for the surface expressions of the STF and SAF, respectively. These are fixed salinity contours whose positions vary in time; their poleward migration rates serve as diagnostics of frontal displacement rather than as formal dynamical definitions of the fronts. The markedly different migration rates of S(35) and S(34), together with the close agreement between the S(35) migration and the independently diagnosed poleward shift of the gyre-edge streamfunction $\psi(0)$, support the interpretation of these isohalines as proxies for the surface expressions of the migrating frontal zones over the study period. The latitude-dependent and basin-dependent SSS trends (Supplementary Fig. 4) further support the interpretation that the observed isohaline shifts reflect frontal-zone displacement rather than a spatially uniform salinity offset."

Response to Reviewer #2

We thank Reviewer #2 for the careful final reading and for confirming that the major concerns are resolved. We note that most items below arose from formatting in the tracked-changes version and were already corrected in the clean manuscript. All has been verified in the current version. The reviewer's comments are shown in blue italics, and our responses are shown in black.

The revised manuscript satisfactorily addresses the major concerns raised in my previous review, especially the careful analysis of the mixed-layer salinity budget, discussion of the residual, and the attribution of salinification to horizontal advection. The added ECCO-based salinity budget analysis, clearer budget methodology, improved ENSO-SAM explanation, and more detailed treatment of the Indian sector and observational uncertainty strengthen the manuscript and support the main conclusions. A few very minor issues are noted below.

Minor comments
(line numbers refer to the tracked changes version of the text)
1. l. 70: xpanded -> expanded

Corrected.

2. l. 70–71: positive TREND in the Southern Annular Mode (SAM) TREND

Corrected.

3. l. 77: density-drivenN

Corrected.

4. standardize unit formatting, e.g. decade-1 vs. decade^{-1}

Confirmed. All instances now use decade^{-1} consistently throughout the manuscript and supplementary material.

5. l. 701- 55 – superscript

Corrected.

6. l. 767 – 56, 57 – superscript

Corrected.

7. l. 783 – 34 – superscript

Corrected.

8. l. 948. "Error! Reference source not found"?

Fixed.

9. l. 1288. "the Zenodo repository at xxx"

The Zenodo repository placeholder has been updated. The MATLAB code for all analyses and figures was provided as a zipped file with a readme.txt for the reviewers' assessment during the previous submission and will be deposited in a public Zenodo repository with a DOI upon publication.

Response to Reviewer #3

We thank Reviewer #3 for the positive assessment of the revised manuscript and for supporting publication. Both remaining suggestions, terminology and spatial framing, have been addressed, as detailed below. The reviewer's comments are shown in blue italics, and our responses are shown in black.

I appreciate the authors' thoughtful responses to my comments and the substantial revisions made throughout the manuscript. I found the review process highly constructive, and I believe the manuscript has significantly improved in response to the comments from all reviewers. I now support publication. I only have two remaining minor comments for the authors to consider.

(1) Minor terminology refinement

The concern regarding the contrast between "dynamic ocean processes" and "atmospheric forcing" has been satisfactorily addressed. I only note that the revised terminology ("thermohaline forcing") occasionally reads somewhat unconventional in this context, and the authors may wish to consider whether terms such as "buoyancy forcing" or "surface freshwater forcing" would provide slightly greater physical clarity. In particular, expressions such as "thermohaline freshwater forcing" (Line 31) feel somewhat awkward conceptually.

We agree that "thermohaline freshwater forcing" is awkward in this context, as the forcing discussed here refers specifically to the salinity effect of evaporation minus precipitation rather than to the combined thermal and haline contributions to density. We have replaced all instances of "thermohaline forcing," "thermohaline tendency," and "thermohaline freshwater forcing" with "surface freshwater forcing" or "surface freshwater tendency," as appropriate.

The Abstract now reads:

"...the dynamical signal must overcome an opposing **surface freshwater** tendency. Driven by poleward-shifting westerlies and a positive Southern Annular Mode trend, this circulation-driven salinification demonstrates that ocean dynamical processes can dominate **surface freshwater** forcing..."

The same change has been applied consistently throughout the Introduction, Results, and Discussion.

(2) Remaining concern regarding the description of salinification spatial structure

Concerning the authors' response to my comment associated with Lines 192–194 of the originally submitted manuscript, specifically their item (iii):

"Domain-mean SSS trends averaged over 40–50°S and 20–35°S have been computed. In the Pacific, the two bands show comparable trend magnitudes, consistent with the reviewer's observation; in the Atlantic, the 40–50°S band trend is stronger. These values have been noted in the text."

I was unable to identify where these values were incorporated into the revised manuscript. Since this diagnostic was introduced as supporting evidence for the revised interpretation, it would be helpful either to briefly report the values in the manuscript or to include the corresponding time series/trends in a supplementary figure.

I appreciate the authors' efforts to revise the wording and avoid implying an overly strong magnitude hierarchy between the 40–50°S and 20–35°S bands. However, I still find that the revised phrasing ("salinification maximum" vs. "secondary salinification") somewhat suggests a robust spatial hierarchy that is not always clearly evident from Fig. 1 and Fig. 4a, particularly given the pronounced longitudinal heterogeneity and the substantial subtropical salinification visible across broad portions of both the Pacific and Atlantic sectors.

More generally, I wonder whether the spatial patterns might be more robustly and simply described relative to the STF/front location itself, rather than through a stronger-versus-secondary hierarchy between fixed latitude bands. To my eye, the figures most consistently support a salinification maximum organized around the STF region and its displacement, whereas the relative magnitude contrast between 40–50°S and 20–35°S appears more longitudinally variable.

I also note that a frontal/STF-centered framing seems more directly aligned with the dynamical interpretation proposed throughout the manuscript (e.g., gyre expansion, STF displacement, frontal migration, and associated advection), and may therefore connect the spatial description more naturally to the underlying mechanism discussed by the authors.

We thank the reviewer for pointing this out. The fixed-band trend values were computed previously but not reported. They are now stated explicitly in the Results, with a new Supplementary Fig. 4 showing the time series and linear trends for both bands in each basin.

The domain-mean values are:

- Pacific, 40-50°S: 0.024 decade^{-1} ($p < 0.05$); 20–35°S: 0.009 decade^{-1} ($p > 0.05$)
- Atlantic, 40-50°S: 0.047 decade^{-1} ($p < 0.05$); 20–35°S: 0.038 decade^{-1} ($p < 0.05$)

These explicit values refine our earlier characterization: rather than the two Pacific bands being "comparable", the 40–50°S trend exceeds the 20–35°S trend in both basins, and the relative magnitude of salinification between the two bands is basin-dependent. In the Pacific, the 40–50°S trend is significant while the 20–35°S trend is not; in the Atlantic, both are significant, with the 40–50°S band the stronger of the two.

Following the reviewer's suggestion, we now frame the spatial pattern relative to the STF rather than as one latitude band consistently salinifying more than another. This STF-centered framing connects more naturally to the gyre-expansion mechanism. We revised the manuscript as follows:

(1) Results, lines 137-144: We added the fixed-band trend values directly to the manuscript to make the diagnostic visible. The added paragraph now reads:

"Domain-mean SSS trends confirm that the relative magnitude of salinification between fixed latitude bands varies by basin (Supplementary Fig. 4). In the Pacific, the 40–50°S trend exceeds the 20–35°S trend (0.024 versus 0.009 decade^{-1}), but only the former is statistically significant ($p < 0.05$). In the Atlantic, both bands show significant positive trends, with the 40–50°S band again the stronger of the two (0.047 versus 0.038 decade^{-1}; both $p < 0.05$). We therefore describe the salinification mainly in relation to the STF and its poleward displacement, because the contrast between the two bands varies in both strength and significance between basins."

(2) Mixed-layer salinity budget section, lines 255-258: We revised the description of the reconstructed budget trend to avoid implying a strict "maximum versus secondary" distinction. The sentence now reads:

"The budget-reconstructed MLS trend C_{TOT} (Fig. 4a) reproduces the dominant spatial features of the observed decadal SSS trend (Fig. 1), including salinification organized around the STF and adjacent frontal transition, additional subtropical salinification at 20-35°S, and patchy negative trends south of the SAF."

(3) Section heading, line 323: We revised the section title from "Why salinification peaks at 40-50°S" to "STF-centered salinification and fixed-band amplification."

(4) Mechanism section, lines 325-339: We revised the opening paragraph to frame the spatial pattern around the STF and frontal transition rather than as one latitude band consistently salinifying more than another. The paragraph now reads:

"The schematic (Fig.5a) outlines the advective pathway. Poleward shift of the subtropical gyres moves the gyre-edge separatrix $\psi(0)$ southward, carrying the high-SSS subtropical zone toward higher latitudes and displacing the STF-centered salinity transition. The fixed 40–50°S band intersects this frontal transition in much of the Pacific and Atlantic and therefore captures a strong salinity response where the meridional gradient is large. However, the relative magnitude of salinification between 40–50°S and subtropical latitudes varies by basin and longitude, so the salinification tracks the migrating STF rather than a fixed latitude band, and is strongest where the 40–50°S band intersects the front."

(5) Discussion, lines 383-385: We revised the summary statement to align the spatial description with the STF-centered mechanism. The sentence now reads:

"The salinification is organized most consistently around the STF and adjacent frontal transition, including the 40-50°S band where the meridional salinity gradient between S(35) and S(34) is strong."

Review of "Subtropical gyre expansion causes Southern Ocean salinification contrary to freshening predictions" by Lisan Yu and John M. Toole, submitted to Nature Communications (manuscript NCOMMS-25-91148).

This manuscript investigates the observed (2004–2024) increase in Southern Ocean surface salinity, which contrasts with the thermodynamic expectation of freshening under an intensifying hydrological cycle ("salty-gets-saltier, fresh-gets-fresher"). However, besides intensifying the hydrological cycle, anthropogenic global warming is also causing changes in climatological wind patterns. These changes are associated with poleward-shifting midlatitude westerlies in the Southern Hemisphere, and a positive trend of the Southern Annular Mode (SAM). In terms of the wind-driven oceanic circulation, this translates to an intensification and poleward shift of Southern Hemisphere subtropical gyres. The authors therefore link this counter-intuitive salinification to the southward migration of Southern Hemisphere subtropical gyres and the associated high-salinity zone, concluding that dynamic ocean circulation processes dominate over thermodynamic surface freshwater forcing (i.e., precipitation vs. evaporation) in determining the Southern Ocean's salinity trend pattern.

I find the paper interesting, timely, and relevant, especially in the context of understanding and considering the interplay among the several cascading effects of anthropogenic climate change. The analyses are strong, results are compelling, and findings are well presented through the figures. I support publication, provided that several core issues are addressed, primarily related to clarity, the faithful description of basin-specific results and aspects of mechanistic attribution.

While the paper convincingly demonstrates a hemispheric-scale salinification band co-occurring with poleward-shifting circulation features (e.g., S(35)/S(34) isohalines, ψ(0), the zero wind stress curl line, ADT contours), some descriptions appear to overgeneralize across the Pacific and Atlantic basins or do not fully reflect the patterns shown in the figures.

In particular, South Atlantic salinity trends exhibit distinct spatial and vertical structures that differ in important ways from their Pacific counterparts. Faithfully describing these basin-specific features — even if they are not the primary focus of the manuscript — would strengthen the paper and potentially enhance its impact, without altering the main conclusions.

In that context, I outline below a possible interpretation of the South Atlantic trends, supported by relevant studies, in case the authors wish to place these results in a broader dynamical framework.

The South Atlantic salinity trend pattern north of the STF — where surface salinification occurs within the "D-shaped" horizontal subtropical gyre domain (Fig. 1), and subsurface salinification follows the bowl-shaped vertical gyre structure (Fig. 2b, lower panel), bordered by freshening trends north of it (Fig. 2b, upper panel) — is consistent with a salinity accumulation in the upper South Atlantic, associated with a weakening of the Atlantic meridional overturning circulation (AMOC) (Zhu and Liu, 2020).

Similar to the processes leading to Southern Ocean salinification, as found in the present paper, salinity accumulation in the southwestern South Atlantic has been shown to be dominated by the convergence of horizontal transports (Pontes and Menviel, 2024). In line with this scenario, by considering the South Atlantic circulation combined response to changing wind and buoyancy forcings, the AMOC weakening is projected to be compensated by a stronger southwestern South Atlantic subtropical gyre (SASG) circulation, alongside increasing inflows from the Indian and Pacific Oceans (Marcello et al., 2023).

These interpretations align with the observed South Atlantic salinification pattern shown in Fig. 1, which is consistent with increased entrainment of salty Agulhas leakage waters into the South Atlantic. Because as the AMOC weakens and the SASG strengthens, this favors increased southward (while decreased northward) advection of salty waters from the Indian Ocean Agulhas leakage at the northern boundary of the SASG.

Comparable large-scale trend patterns have been reported in Marcello et al. (2023), who use an ensemble mean to investigate the Atlantic circulation response to external forcing (e.g., compare their Fig. 1 and Fig. S7 with Figs. 3a and S2b here). Their Fig. S6 further illustrates wind pattern changes consistent with the spin-up and poleward expansion of Southern Hemisphere subtropical gyres.

Importantly, such regional South Atlantic circulation dynamics may contribute to reinforcing the Southern Ocean salinification trend pattern identified in the present study, which has thus far been primarily linked to hemispheric-scale wind changes. These mechanisms further support the authors' broader conclusion that dynamic ocean processes can dominate over thermodynamic surface freshwater forcing in shaping regional salinity patterns. Furthermore, I believe their consideration aligns with the author's view of recognizing the interplay among the several cascading effects of modern anthropogenic global warming, as I mentioned.

Below, I provide specific comments that illustrate the general comments highlighted previously, mostly concerning clarity and the faithful alignment between text and figures. Should any of my concerns arise from misinterpretation, I would welcome clarification from the authors and look forward to their responses.

References mentioned:

Zhu, C., & Liu, Z. (2020). Weakening Atlantic overturning circulation causes South Atlantic salinity pile-up. *Nature Climate Change*, *10*(11), 998-1003. https://www.nature.com/articles/s41558-020-0897-7

Pontes, G. M., & Menviel, L. (2024). Weakening of the Atlantic Meridional Overturning Circulation driven by subarctic freshening since the mid-twentieth century. *Nature Geoscience*, *17*(12), 1291-1298. https://www.nature.com/articles/s41561-024-01568-1

Marcello, F., Tonelli, M., Ferrero, B., & Wainer, I. (2023). Projected Atlantic overturning slow-down is to be compensated by a strengthened South Atlantic subtropical gyre. *Communications Earth & Environment*, *4*(1), 92. https://www.nature.com/articles/s43247-023-00750-4

Specific comments:

- Lines 48-51:
"Satellite altimetry shows large positive sea surface height (SSH) trends in the western subtropical basins, particularly the South Pacific, with Argo potential density anomalies extending to at least 2000 m and accounting for more than half of the total trend 14,19,20,21."

The final clause of this sentence is potentially ambiguous. As written, it appears to state that Argo-derived potential density anomalies extending to ~2000 m account for more than half of the observed SSH trend. However, the physical link between deep density anomalies and SSH (e.g., via steric contribution) is implicit and not clearly stated. In addition, the meaning of 'accounting

for' and the reference 'total trend' (SSH vs. steric SSH) are unclear. A brief clarification would improve readability for the broad Nature Communications readership.

- Lines 61-65:
"Early syntheses, constrained by sparse pre-Argo hydrography, appeared consistent with this expectation, showing broad Southern Ocean freshening30,31. Yet recent high-resolution observations reveal a more complex picture, raising a fundamental question: can dynamic ocean circulation changes overwhelm the thermodynamic signature of an intensifying water cycle?""

The sentence 'Yet recent high-resolution observations reveal a more complex picture' could benefit from clarification. As written, it reads as referring to previously published observations, whereas the following paragraph introduces the authors' own observational evidence. Clarifying whether this statement refers to prior studies, the present work, or both would improve narrative continuity and avoid a momentary misreading by the reader.

- Lines 68-69:
"We show that poleward migration of the subtropical gyres drives this paradoxical salinification…"
For clarity, I suggest specifying that this refers to the poleward migration of "southern subtropical gyres", as the analyses focus on the Southern Hemisphere.

- Line 77:
"We also assess how ENSO and the SAM modulate the shift on interannual and decadal timescales. "
Similarly, I suggest specifying which 'shift' is being referred to here (e.g., the gyres' position shift).

- Lines 78-80:
"Our aim is to provide a falsifiable, mechanism-based account of midlatitude Southern Ocean salinity change that demonstrates how dynamic ocean processes can dominate over atmospheric forcing in determining regional salinity patterns"

I suggest replacing "atmospheric forcing" for "thermodynamic surface freshwater forcing" here, since it is conceptually misleading, because, actually, it is also "atmospheric forcing" (wind-driven circulation changes) that is causing the so-called "dynamic ocean processes"…

- Figure 2 - caption:
I recommend defining the abbreviations used in the panel titles, or writing them in full. 'PAC' and 'ATL' are not defined in the caption, and the meaning of 'SAL' is unclear (e.g., whether it refers to salinity in general or to the linear salinity trend).

- Lines 109-111:
"In the Pacific and Atlantic, the largest positive SSS trends coincide with the sharp SSS gradient between S(35) and S(34) near 40–50°S (the STF–SAF corridor)."

The statement that 'the largest positive SSS trends' coincide with the STF–SAF corridor appears appropriate for the Pacific basin, but seems less accurate for the South Atlantic. In this basin, the largest SSS trends occur predominantly northward of the STF, within South Atlantic subtropical gyre latitudes. It may therefore be more accurate to refer to 'large positive SSS trends', or to differentiate more explicitly between the Pacific and Atlantic basins in this sentence.

- Line 114:
"Equatorward of S(35), the salty subtropics show weaker, patchier increases;"

Again, this statement appears to apply to the South Pacific, but not to the South Atlantic, where equatorward of S(35) relatively strong SSS trends persist.

- Lines 114-115:
"Equatorward of S(35), the salty subtropics show weaker, patchier increases; poleward of S(34), trends diminish in the Atlantic but remain locally significant in the Pacific."

Based on the use of expressions such as 'patchier increases' and 'remain locally significant', I assume this sentence refers to the lower panels rather than the upper panels discussed earlier in the paragraph. If so, it would help to explicitly indicate this shift in panel reference.

However, when considering the lower panels, it is not clear that trends poleward of S(34) 'diminish in the Atlantic but remain locally significant in the Pacific'. In particular, the Pacific and Atlantic trend patterns poleward of S(34) appear broadly similar, especially in terms of trend magnitude. In terms of spatial distribution, the patterns are also quite similar, except for the layer between ~500–1000 m, where trends are more heterogeneous in the Atlantic, implying non-statistically significant trends over the broader layer between ~400–1100 m, according to the stippling.

Unless the authors intended this statement to refer to the upper panels, where trends do diminish south of ~55°S in the Atlantic while continuing to increase south of the SAF in the Pacific. However, in that case, the use of 'locally significant' would be confusing, as the upper panels do not display information on trend significance, whereas the lower panels do.

Clarifying which panels this sentence refers to, and revisiting the basin-to-basin contrast described here, would improve consistency between the text and Fig. 2.

- Lines 115-117:
"The climatologically fresh 40–50°S band thus experiences the largest salinification, a pattern fundamentally inconsistent with thermodynamic forcing alone."

Consistent with points raised above, the statement that the 40–50°S band experiences 'the largest salinification' does not appear fully consistent for the Atlantic basin, where the 30–40°S band shows salinification of comparable magnitude, if not larger. It may therefore be more accurate to state that the 40–50°S band experiences **large** salinification, or to qualify this statement more explicitly by basin.

- Lines 119-120:
"Depth–latitude trend sections (Fig.2, lower panels) show a vertically coherent salinification maximum centered near 40–50°S,"

Similarly, I suggest rephrasing the end of this sentence to "centered near 35–50°S", which provides a more faithful description of the trend patterns referred to here.

- Lines 119-127:
"Equatorward of *S*(35), trends weaken and a shallow freshening develops in the upper 100–200 m, while south of *S*(34) patchy subsurface salinification persists. Overlaid *S*(34)–*S*(37) isohalines show modest poleward and downward displacements of *S*(34) and *S*(35) along their poleward flanks, indicating increased subtropical influence across the STF into the STF-SAF corridor. The 40–50°S salinification is therefore not confined to the surface, but represents a redistribution of water masses throughout the upper ocean."

I find the description of the lower panels of Fig. 2 in this passage to be neither fully accurate nor internally consistent.

First, I do not believe that the most relevant results highlighted by these panels are being adequately emphasized in the text. Second, I do not think that the Pacific and Atlantic results north of the STF can be described together in a generalized way, as they differ substantially.

In particular, the statement “Equatorward of S(35), trends weaken” appears overly generalized. Trends do not uniformly weaken equatorward of S(35), as they sharply increase north of ~20°S in the Pacific, which still lies equatorward of S(35)/the STF. Moreover, in the Atlantic, trends actually strengthen immediately equatorward of S(35) and only weaken equatorward of ~35°S.

In addition, the statement that “a shallow freshening develops in the upper 100–200 m” is vague and potentially confusing. In the Pacific, only a very minor freshening appears in the upper ~50 m, confined to a relatively narrow meridional band (~21–27°S), whereas a much more pronounced and extensive freshening occurs between ~300–500 m, extending well northward of the STF—an outstanding feature that is not mentioned in the text. In contrast, in the Atlantic, freshening occurs primarily between ~100–250 m north of ~20°S, following a markedly different pattern from the Pacific, while between ~300–500 m north of the STF, modest salinification persists, in contrast to the large freshening feature observed in the Pacific over the same depth layer.

These differences complicate attributing “a shallow freshening in the upper 100–200 m” simultaneously to both basins.

Regarding the passage “Overlaid S(34)–S(37) isohalines show modest poleward and downward displacements of S(34) and S(35) along their poleward flanks”, several points require clarification. First, if the discussion focuses only on S(34) and S(35), it may be clearer to refer directly to these isohalines rather than introducing the broader S(34)–S(37) range. Second, S(34) is sparsely present in both panels (except for a short, shallow contour extending southward of the SAF) and instead shows behavior inconsistent with the stated poleward and downward displacements. Third, while S(35) shows an overall poleward and downward displacement in the Atlantic, in the Pacific the poleward displacement is mainly confined to the upper ~150 m, whereas downward displacement occurs primarily north of ~15°S.

A more detailed, basin-specific description would more faithfully reflect the patterns shown in Fig. 2. For example, in the Pacific, the S(34.5) and S(35) isohalines exhibit poleward displacements mainly in the upper ~300 m, while S(35) and S(35.5) show downward displacements north of ~20°S. In the Atlantic, north of the SAF, S(34.5)–S(36.5) display more systematic poleward and downward displacements.

Furthermore, I suggest aligning the text and figure nomenclature. The text refers to S(34)/S(35), while the figure highlights the STF/SAF using blue and green dashed lines. One option would be to relabel the dashed lines as S(34)/S(35), consistent with the terminology used in the text; alternatively, the text could be revised to refer to the STF/SAF instead. At present, it is actually unclear whether references to S(34)/S(35) correspond to the dashed STF/SAF lines or to the black and magenta isohaline contours. Clarifying and standardizing this distinction would help avoid confusion.

- Line 142: There appears to be a typographical or sentence break error at ‘reorganization In ADT’. I assume ‘In ADT’ is intended to start a new sentence.

- Line 145:
The phrase ‘poleward-displaced 20–25 dyne cm means in 2020-2024’ is unclear. Do the authors refer to poleward shifts of the 20–25 dyne cm dynamic height contours during 2020-2024 with respect to 2004-2008?

- Supplementary Figure S3 - caption:
There is a typo in “25-pointrunning”

- Lines 152-153:
“In each, opposite-signed trend anomalies straddle ψ(0)”
I suggest adjusting to “opposite-signed large-scale trend anomalies”, as this feature is not immediately apparent from a detailed visual inspection of Fig. 3a.

- Lines 192-194:
“The observed pattern shows a salinification belt near 40–50°S, weaker salinification from 20–35°S, and patchy freshening south of the SAF. The budget reconstruction reproduces the overall features (Fig.4a,b)”

I am somewhat confused by this passage. It is unclear whether both sentences refer to Fig. 4a,b, or whether the first sentence refers to the observed pattern shown in Fig. 1, while the second sentence intended to compare Fig. 4a,b against that pattern.

In either case, I find it difficult to clearly identify the features described in Fig. 4a,b. In particular, I do not clearly see a continuous “salinification belt near 40–50°S” that stands out relative to other salinity trend features in these panels. Likewise, the description of “weaker salinification from 20–35°S” is not obvious. For example, over 20–35°S and ~150°E–150°W, salinification appears relatively strong in Fig. 4a. Moreover, based on Fig. 4b, salinification over 20–35°S seems more spatially continuous than over 40–50°S.

I therefore recommend revisiting the description of these figures to ensure that it closely reflects the patterns actually shown in Fig. 4a,b, rather than a more schematic or expected representation of the results.

One possible way to assess whether salinification is indeed stronger over 40–50°S and weaker over 20–35°S would be to derive time series of mean salinity over these latitude bands. This could help clarify the relative behavior between regions and aid the description and interpretation of the results.

- Figures 3a and 4a,b:
The “60ºE” x-axis label should be “60ºW” in these figures. I noticed this error also occurs in Figures S4 and S6.

- Lines 196-210:
“Horizontal advection is the leading driver across the STF–SAF corridor, demonstrating that dynamic ocean circulation overwhelms thermodynamic forcing in this region. Ekman advection maximizes in a zonal band near 40–50°S, while geostrophic advection peaks along western boundary current extensions and frontal jets (e.g., downstream of the EAC extension and at the BMC) (Supplementary Fig.S6a-b). As in Fig.2 (upper panels), the meridional SSS gradient |¶S/¶y|

is steepest between S(35) and S(34). Consequently, the meridional Ekman term *v*EK¶S/¶y dominates **u**EK×ÑS, producing the strongest saline advection across the STF. These advective maxima align with the poleward displacement of ψ(0) and the ψ-trend dipole (Fig.3), linking the budget signal to gyre migration. Entrainment and FWF modulate but are secondary at 40–50°S (Supplementary Fig.S6c). Entrainment salinifies north of the STF and freshens south of the SAF, consistent with deeper mixing entraining saline ventilated-thermocline waters to the north and fresher Antarctic Intermediate Water (AAIW) -influenced waters 45 to the south (Fig.2, lower panels). FWF salinifies the interior subtropics and weakly freshens poleward (Supplementary Fig.S6d), as expected from the precipitation-evaporation flux, but its contribution is minor compared to advection at 40–50°S. Diffusion is small and spatially noisy (not shown).”

This is a long and very heavy paragraph that could use flow improvement and further clarification. My main comments below revolve around one core issue, that mechanistic attribution seems to be asserted more strongly than it is demonstrated or clearly explained.

- First, I suggest the authors start this paragraph by initially clarifying they’re discussing Fig. S6. Something along the lines “The linear trends of main budget terms are shown in Fig. S6”. This would help to first anchor the reader to understand that the upcoming discussion refers to results in this figure. Because when I read the first sentence, I automatically thought the authors were referring to Fig. 4c—considering that they were discussing Fig. 4a,b at the end of the previous paragraph.

- I believe the authors are allowed to use the simplified nomenclature “Fig. S6” instead of “Supplementary Fig. S6” — this would help simplify the paragraph a little, since the different panels of Fig. S6 are called individually later on.

- The use of “horizontal advection” in the first sentence is confusing, especially for non-experts, as Fig. S6 only shows Ekman and geostrophic advection as separate terms. I suggest clarifying whether ‘horizontal advection’ refers to the sum of Ekman and geostrophic advection, and to state this explicitly in the text.

Concerning this passage: “As in Fig.2 (upper panels), the meridional SSS gradient |¶S/¶y| is steepest between S(35) and S(34). Consequently, the meridional Ekman term *v*EK¶S/¶y dominates **u**EK×ÑS, producing the strongest saline advection across the STF. ”:

- Similarly, it is confusing to suddenly address “the meridional Ekman term” — which is not shown separately anywhere in the paper. This might induce non-expert readers, particularly, to go and look for this term somewhere in the figures/text. Alternatively, I suggest rephrasing it to “the meridional component of the Ekman advection term (not shown separately)”.

- As written, “As in Fig.2 (upper panels), the meridional SSS gradient”, it gives the impression that the authors are comparing Fig. S6 with Fig. 2, which is not the case. I would suggest rephrasing this passage to “Because the meridional SSS gradient |¶S/¶y| is steepest between S(35) and S(34), the meridional … . ”;

- However, the opposite seems to be the case—i.e., meridional SSS gradients being steepest between S(35)-S(34) potentially as a consequence (at least partially) of the Ekman advection term being dominated by its meridional component…

- Still, as the authors do not provide concrete evidence for this claim (i.e., for the link between steepest SSS gradients between S(35)-S(34) and the dominance of the meridional component in

the Ekman advection term), they should clarify this is their assumed hypothesis. This could be done by simply clarifying that “the (implied or suggested) dominance of the meridional component in the Ekman advection term” aligns with “strong saline advection across the STF” and “steepest SSS gradients between S(35)-S(34) (Fig. 2, upper panels)”.

- About “Entrainment and FWF modulate but are secondary at 40–50°S (Supplementary Fig.S6c).” — it is unclear what does entrainment modulate?! Also, I believe Fig. S6c, d should be referenced here, since FWF is also being addressed.

- About “Entrainment salinifies north of the STF and freshens south of the SAF, consistent with deeper mixing entraining saline ventilated-thermocline waters to the north and fresher Antarctic Intermediate Water (AAIW) -influenced waters 45 to the south (Fig.2, lower panels).”:

- I suggest Fig. S6c is then individually referenced here: “Entrainment salinifies north of the STF and freshens south of the SAF (Fig. S6c)”

- However, in my opinion, the authors are again overgeneralizing results for the Pacific and Atlantic basins, with a focus on the Pacific—thus neglecting important and differentiated results for the Atlantic basin. More specifically, if one looks at the Atlantic in Fig. S6c, entrainment actually salinifies predominantly *in between* the STF and SAF, and north of the STF to a much lesser degree; while it does not freshen immediately south of, or along, the SAF, as in the Pacific.

- “FWF salinifies the interior subtropics and weakly freshens poleward (Supplementary Fig.S6d)”. Again, when comparing the Pacific and Atlantic basins in Fig. S6d, opposed patterns are observed over subtropical latitudes, with the Pacific showing salinification mainly over the South Pacific Subtropical Gyre center (analogous to “interior subtropics”), and the Atlantic in turn showing freshening over the corresponding region, and salinification along its boundary currents instead. Additionally, there is basically no freshening south of the STF in the Atlantic domain. At least it seems not to be statistically significant. I further suggest the authors include stippling in Fig. S6 to indicate where linear trends are statistically significant. In this case, stippling could be used only over regions where trends are not significant instead, not to overwhelm the figure aesthetics.

- Lines 212-213:
“The dominant-term map (Fig.4b) identifies, at each grid point, the process giving the largest contribution to the observed SSS tendency trend.”

- “The dominant-term map (Fig.4b)” — the correct reference here is Fig. 4c.

- The phrase ‘SSS tendency trend’ is somewhat confusing, as ‘tendency’ already implies a rate of change. It may be clearer to refer simply to the ‘observed SSS trend’, or to clarify whether this refers to the trend of the SSS tendency itself (which would correspond to a second derivative in time, and does not appear to be the intended meaning here).

- Lines 213-215:
“It confirms that horizontal advection, primarily Ekman, dominates along the STF, with geostrophic dominance confined to boundary currents and frontal jets. Poleward of the SAF, Ekman remains prevalent, with localized patches where entrainment or FWF lead. Over the interior subtropics (~20–35°S), FWF and entrainment co-lead, while advection contributes in localized patches.”

- Concerning the first sentence, when considering both the Pacific and Atlantic basins, I would say that horizontal advection (both Ekman and geostrophic) dominates along/around the STF, with Ekman advection predominating only over the Pacific domain. I would also say that geostrophic advection seems to dominate along the SAF, mainly in the Pacific, but also in the Atlantic, to a lesser extent.

- Regarding the second sentence ("Poleward of the SAF, Ekman remains prevalent, with localized patches where entrainment or FWF lead."), I would include geostrophic advection to "entrainment or FWF", as it appears poleward of the SAF maybe even to a higher extent than FWF, for example.

- For the third/last sentence, I would just replace "advection" with "horizontal advection", for consistency with the terminology used elsewhere.

- Lines 223-226:
"Importantly, horizontal advection (Ekman plus geostrophic) accounts for more than one-third of the domain-mean trend, establishing dynamic ocean circulation as the primary driver of Southern Ocean salinification and relegating surface freshwater forcing to a secondary role 7, 46."

- I would add here that, besides 'horizontal advection (Ekman plus geostrophic) accounting for more than one-third of the domain-mean trend' (Fig. 4d), it clearly dominates south of ~35ºS in both the Pacific and Atlantic basins (as according to Fig. 4c)—this regional dominance being therefore the main feature "establishing dynamic ocean circulation as the primary driver of Southern Ocean salinification…", rather than the full domain-mean contribution alone.

- Lines 228-230:
"Equally important is the spatial structure: maxima in vEK∂S/∂y co-locate with the climatologically steep S(35)–S(34) gradient (Fig.2), implying that frontal migration projects most strongly onto the fixed 40–50°S band."

- As raised before, the claim that "maxima in vEK∂S/∂y co-locate with the climatologically steep S(35)–S(34) gradient" does not appear to be sufficiently supported, as I assume that "vEK∂S/∂y" is not shown individually, anywhere in the manuscript. I suggest clarifying how this co-location is demonstrated, or alternatively moderating the wording of this statement.

- Discussion:
- I suppose the authors should mention again in the Discussion that their Southern Ocean results are focused on the Pacific and Atlantic basins.

- Line 288:
"The 40–50°S salinification peak arises from the advective pathway."
- I suggest adjusting to: "...from horizontal advective pathways"

- Lines 303-304:
"In a warming Southern Ocean 25" — the link to reference "25" is missing here.

- Reference suggestion:
The authors may find it useful to consider the paper of Ge et al. (2023), which states that "wind-driven alterations in the geostrophic circulation cause SSS advection across zonal salinity gradients. These processes drive SSS changes that are opposite in sign to local FWF changes and complicate the regional SSS-FWF relationship in the long-term change since 1970." (https://agupubs.onlinelibrary.wiley.com/doi/pdf/10.1029/2023JC020207).
